# Gaining comprehensive biological insight into the transcriptome by performing a broad-spectrum RNA-seq analysis

**DOI:** 10.1038/s41467-017-00050-4

**Published:** 2017-07-05

**Authors:** Sayed Mohammad Ebrahim Sahraeian, Marghoob Mohiyuddin, Robert Sebra, Hagen Tilgner, Pegah T. Afshar, Kin Fai Au, Narges Bani Asadi, Mark B. Gerstein, Wing Hung Wong, Michael P. Snyder, Eric Schadt, Hugo Y. K. Lam

**Affiliations:** 1 Roche Sequencing Solutions, Belmont, CA 94002 USA; 2 Department of Genetics and Genomic Sciences, Icahn School of Medicine at Mount Sinai, New York, NY 10029 USA; 3 Department of Genetics, Stanford University School of Medicine, Stanford, CA 94305 USA; 4 Department of Electrical Engineering, Stanford University, Stanford, CA 94305 USA; 5 Department of Internal Medicine, University of Iowa, Iowa City, IA 52242 USA; 6 Computational Biology and Bioinformatics, Yale University, New Haven, CT 06520 USA; 7 Statistics; Health Research and Policy, Stanford University, Stanford, CA 94305 USA

## Abstract

RNA-sequencing (RNA-seq) is an essential technique for transcriptome studies, hundreds of analysis tools have been developed since it was debuted. Although recent efforts have attempted to assess the latest available tools, they have not evaluated the analysis workflows comprehensively to unleash the power within RNA-seq. Here we conduct an extensive study analysing a broad spectrum of RNA-seq workflows. Surpassing the expression analysis scope, our work also includes assessment of RNA variant-calling, RNA editing and RNA fusion detection techniques. Specifically, we examine both short- and long-read RNA-seq technologies, 39 analysis tools resulting in ~120 combinations, and ~490 analyses involving 15 samples with a variety of germline, cancer and stem cell data sets. We report the performance and propose a comprehensive RNA-seq analysis protocol, named RNACocktail, along with a computational pipeline achieving high accuracy. Validation on different samples reveals that our proposed protocol could help researchers extract more biologically relevant predictions by broad analysis of the transcriptome.

## Abstract (web summary)

RNA-seq is widely used for transcriptome analysis. Here, the authors analyse a wide spectrum of RNA-seq workflows and present a comprehensive analysis protocol named RNACocktail as well as a computational pipeline leveraging the widely used tools for accurate RNA-seq analysis.

## Introduction

The popularity of high-throughput next-generation sequencing (NGS) ushered a new era in transcriptome analysis with RNA-seq. A widespread application of RNA-seq requires workflows tuned to the sequencing technologies involved, sample types, desired analysis as well as the availability of genomic and computational resources. Depending on the workflow used, the accuracy, speed, and cost of analysis can vary significantly. Thus, it is crucial to study the tradeoffs involved at different steps of an RNA-seq analysis to get the best accuracy subject to the cost and performance constraints. Furthermore, figuring out the optimal workflow is even more challenging since, in general, the best overall approaches may have sub-optimal performance for a specific data set in terms of a specific measure, which necessitates a comprehensive analysis of workflows using a wide variety of data sets.

Several efforts have tried to compare the performance of different RNA-seq analysis tools^1–9^. However, these studies have mostly focused on a single RNA-seq analysis step, or their workflow analyses^3, 4^ were limited to one or two steps such as alignment and quantification. Thus, a comprehensive and systematic analysis of the RNA-seq data from different perspectives can contribute significantly toward extraction of maximal insights from RNA-seq data.

To address the limitations of previous studies, we propose a comprehensive RNA-seq protocol in which we thoroughly investigated all the major steps of an RNA-seq analysis and evaluated combinations of algorithms across different steps in terms of accuracy, efficiency, and consistency.

Along with the investigated protocol, we propose the RNACocktail pipeline achieving high accuracy. We further validate the proposed pipeline in detecting biologically relevant differentially expressed genes on different samples, as well as clinically important transcripts. Our analysis reveals the significance of the proposed pipeline in gaining biological insights concerning the transcriptome. The computational pipeline is open-sourced and available at http://bioinform.github.io/rnacocktail/.

## Results

### Data sets

For a comprehensive evaluation, we used diverse types of RNA-seq data in our analysis. Supplementary Table 1 summarizes the data sets used in this study. These data sets include 15 Illumina and Pacific Biosciences (PacBio) data sets from normal human sample NA12878^10^, human MCF-7 breast cancer cell^11^, H1 human embryonic stem cell (hESC)^12^, and the Sequencing Quality Control Consortium (SEQC) data set^8^.

### RNA-seq analysis protocol

**Fig. 1:**
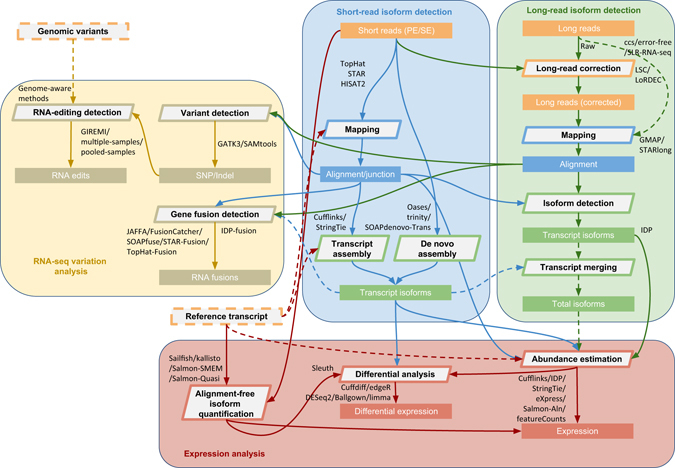
The RNACocktail analysis protocol. RNACocktail is a comprehensive protocol of RNA-seq data analysis. The figure summarizes the widely used approaches for the key steps over the broad spectrum of RNA-seq analysis and also succinctly captures the possible workflows one can use to analyse RNA-seq data

Figure 1 illustrates our proposed protocol for comprehensive analysis of RNA-Seq data that uses state-of-the-art approaches in each step (tool names and versions used are listed in Supplementary Table 2). In the following results, we will also elaborate each step in detail.

### Isoform detection using short reads

Identifying the set of expressed transcripts is typically the first step in an RNA-seq analysis. This generally involves aligning the reads to an appropriate reference followed by constructing the transcripts from the read alignments. Either a genomic reference^13–17^ or a transcriptomic reference^18^ can be used. While a genomic reference enables the detection of novel transcripts, it also requires the spliced alignment of reads that is computationally expensive. In contrast, read alignment against a transcriptomic reference is easier but does not allow the detection of novel transcripts. If no reliable reference exists for the species, de novo transcript assembly can be used to identify the transcripts^19–24^.

### Reference-based transcript identification

**Fig. 2:**
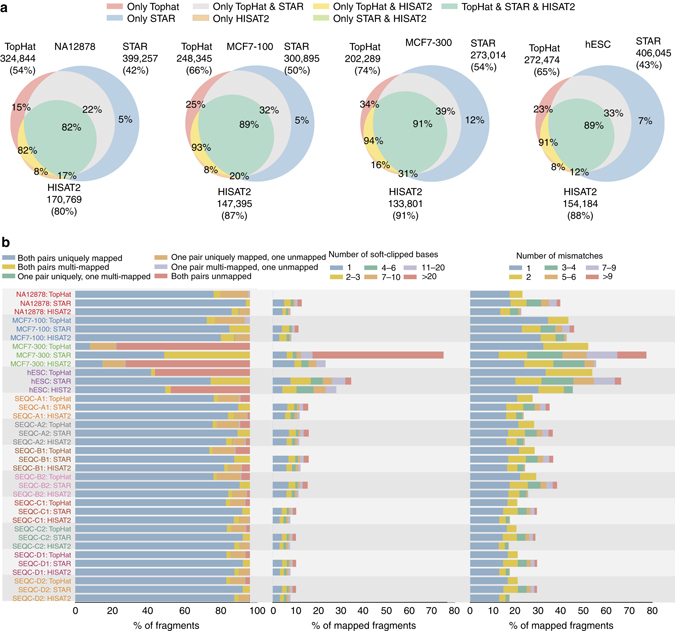
Performance of different alignment schemes. **a** Overlap between the detected splice junctions by different schemes and their validation rate on reliable junctions in dbEST database^74^. A reliable EST junction set consists of junctions supported by at least two ESTs. The sizes of the circles reflect the number of junctions called by each scheme. For each tool, the number of junctions called and the validation rates (in parentheses) are shown. Validation rates for each subset of junctions are also shown on the Venn diagram. **b** Read mapping analysis: distribution of mapping status of sequenced fragments (*left*) (for NA12878, MCF7, and SEQC samples, mapping status for paired-end reads are shown, while for hESC, the distribution reflects percentage of uniquely mapped (*blue*), multi-mapped (*orange*), and unmapped (*red*) single-end reads), distribution of number of soft-clipped bases in mapped fragments (*middle*), distribution of the number of mismatches in mapped fragments (*right*)

*Alignment and junction prediction* Spliced alignment of RNA-seq reads to a genomic reference splices the reads across the exon–intron boundaries. Here we assessed the performance of TopHat^13^, STAR^14^ (using 2-pass option), and HISAT2^15^, the most widely used and efficient spliced aligners on the short-read Illumina hESC, NA12878, SEQC, and 100 and 300-bp MCF7 data sets (Fig. 2; Supplementary Figs. 1–3).

HISAT2 consistently had the largest junction validation rate on all samples, although it had fewer total validated calls than TopHat or STAR (Fig. 2a; Supplementary Figs. 1–3). STAR consistently had the highest fraction of uniquely mapped read pairs, especially on MCF7-300, presumably due to increased read length (Fig. 2b). STAR either mapped or discarded both paired-ends and avoided mapping single ends, unlike TopHat and HISAT2. On the other hand, STAR also yielded lower-quality alignments with more soft-clipped and mismatched bases (Fig. 2b). TopHat does not allow for truncating the reads (Fig. 2b). While these results confirm previous findings^1^, results from the longer read sample (MCF7-300) and the single-ended read sample (hESC) strongly illustrate STAR’s higher tolerance for accepting mismatches and soft-clipping to align more reads in comparison to TopHat and HISAT2 that leave a large fraction of reads unmapped (Fig. 2b). On average, HISAT2 was 2.5 and ~100× faster than STAR and TopHat, respectively (Supplementary Table 3).

*Alignment-based transcriptome assembly*. After spliced alignment, the set of expressed transcripts can be identified using transcriptome assembly. Here we focused on two widely used alignment-based transcriptome discovery tools, namely, Cufflinks^16^ and StringTie^17^. As input to these assemblers, we used all three aligners discussed above. For both assemblers, the Ensembl reference transcriptome annotation^25^ was provided as the guide.

In addition to short-read isoform prediction approaches, our earlier work, the isoform detection and prediction (IDP) (Isoform Detection and Prediction) isoform prediction tool^12^, was also studied. IDP uses a hybrid approach that employs short-read alignment to assist long-read isoform detection. IDP was evaluated with the long-read alignments from GMAP^26^ and STARlong, and short-read alignments from TopHat, STAR, and HISAT2. To analyse the improvements, if any, gained by merging isoforms predicted by long-read and short-read assemblers, we also evaluated the performance of the union of transcripts from both short reads and IDP. Additionally, the long-read-only isoform predictions of Iso-Seq^27^ algorithm, the default PacBio transcriptomics pipeline, were also obtained for the same MCF-7 sample^11^ and computed for the NA12878 sample and included in the analysis.

We measured accuracy by comparing the predicted isoforms against the reference transcriptome annotation in GENCODE v19^28^. Isoforms missing in the reference were considered false positives (FPs).

**Fig. 3:**
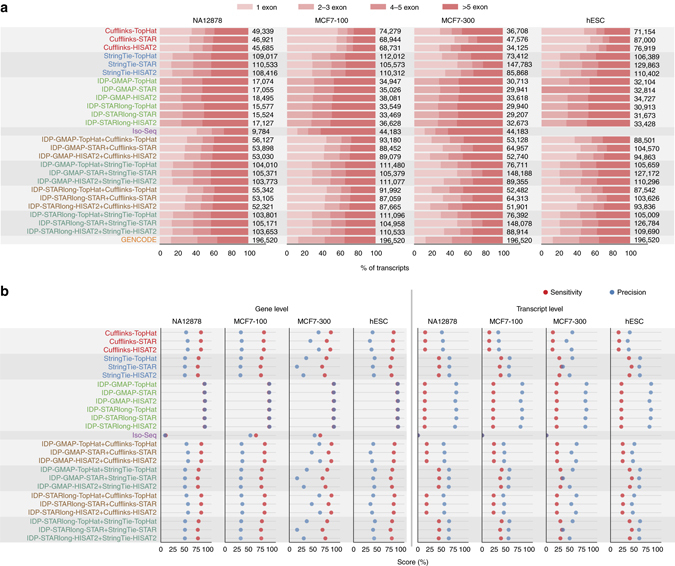
Performance of different transcriptome reconstruction schemes. **a** Distribution of number of exons per transcripts for different transcriptome reconstruction algorithms. Labels reflect the assembler, the long-read aligner (for IDP), and the short-read aligner used, respectively, with “-” separation. **b** Sensitivity and precision of different transcriptome reconstruction approaches at gene and transcript levels. The GENCODE reference transcriptome annotation is used as the truth set. The evaluations on a more recent update of MCF7 sample using the Iso-Seq pipeline resulted in a similar performance with only slight improvement. The union approaches that combined predictions from short reads and long reads (shown with a “+” in the label) slightly improved the performance of short-read isoform prediction schemes

Cufflinks and StringTie reported many single-exon transcripts (Fig. 3a; Supplementary Figs. 4 and 5), which were mostly FPs (Supplementary Fig. 6). StringTie predicted 50–200% more transcripts than Cufflinks. IDP reported the fewest exons consistently across different samples, as it does not report single-exon genes by design. On the multi-exon transcripts, though, it had, on average, similar numbers of calls to Cufflinks (Fig. 3a; Supplementary Fig. 5). Moreover, IDP’s exon count distribution better resembled that of GENCODE especially for multi-exon transcripts (Fig. 3a). On average, nearly 94% of Iso-Seq algorithm’s single exon transcripts and 77% of its multi-exon transcripts were missing from GENCODE. This may reflect the tendency of the Iso-Seq method towards less accurate assembly despite higher sensitivity in detecting novel isoforms.

For the MCF7-300 sample, STAR predicted more calls than other aligners (Fig. 3a; Supplementary Fig. 5), possibly due to its capability in handling longer reads. Using the GMAP and HISAT2 long-read and short-read aligners for IDP resulted in more isoforms.

Unlike short-read assemblers, IDP tended to detect multiple isoforms per gene (Supplementary Fig. 7). Compared with Cufflinks, StringTie, on average, predicted 50 times more genes with more than five isoforms per gene. StringTie’s output also best matched the distribution of number of isoforms per gene observed in GENCODE (Supplementary Fig. 7).

For gene level assessment, IDP achieved the best precision and sensitivity across all samples (Fig. 3b; Supplementary Figs. 8 and 9). Additionally, Cufflinks was more sensitive and precise than StringTie. On the MCF7-300 sample, there was more variation between the performance of the aligners, where TopHat and HISAT2 outperformed STAR. Iso-Seq algorithm had the least sensitivity while its precision was in between IDP and short-read approaches.

IDP outperformed all other techniques by more than 20% in transcript level precision (Fig. 3b). However, as its predictions were limited to most accurate multi-exons, it had less sensitivity than StringTie but higher sensitivity than Cufflinks. Among the short-read assemblers, StringTie had, on average, 11% better transcript-level precision and 25% better transcript-level sensitivity than Cufflinks (Fig. 3b; Supplementary Figs. 8 and 9). Similar behavior was observed for intron-chain level accuracy (Supplementary Figs. 10 and 11). Iso-Seq had close-to-zero precision and recall at transcript level, because of the high error rate in the reconstructed transcripts. For the less-error-sensitive measure of intron-chain level accuracy, Iso-Seq still yielded the least precise and sensitive predictions. For StringTie and IDP, the genes predicted with more introns were more likely to represent novel isoforms, which was consistent with previous studies using long reads^29, 30^ (Supplementary Fig. 12). On average, less than 46% of FP calls by Cufflinks, StringTie, and IDP, and less than 15% of FP calls by Iso-Seq approach could be validated by other schemes (with different assembly approaches) (Supplementary Fig. 13).

Performance assessment of different techniques in predicting 3681 novel isoforms present in GENCODE v19 but missing in the Ensembl annotation revealed that StringTie recovered the most novel isoforms (on average, 2.5× and 6.5× that of Cufflinks and IDP, respectively) (Supplementary Fig. 14). For IDP, using GMAP long-read alignment recovered 15% more novel isoforms than STAR. When the latest GENCODE release v25 was used, the FP rate improved by less than 1% (Supplementary Fig. 15).

StringTie was the fastest tool and finished assembly ~60× and ~50× faster than Cufflinks and IDP (when the inputs were error-corrected and aligned), respectively (Supplementary Table 4).

We observed that, unlike previous studies^2^, in more challenging examples like MCF7-300, STAR reported a much higher number of transcripts (mostly single exons) but with a high FP rate (Fig. 3a; Supplementary Figs. 4 and 5).

### De novo transcript assembly

When lacking a reference genome or transcriptome, de novo assembly of reads can be used to construct the transcripts. Here we analysed the three widely used de novo transcript assembly tools Trinity^19^, Oases^20^, and SOAPdenovo-Trans^21^. As suggested in ref. ^19^, for practical memory requirements, we used the reads normalized according to depth of sequencing coverage as input to all methods. Due to better memory usage, we analysed SOAPdenovo-Trans on the whole MCF7-300 and NA12878 samples as well (SOAPdenovo-Trans-ALL).

**Fig. 4:**
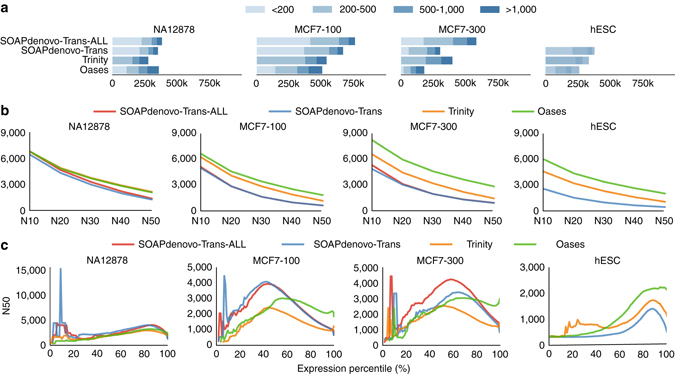
Performance of different de novo transcriptome assembly techniques. **a** Distribution of transcript length. **b** N10-N50 values. (Nx is the contig length for which at least x% of the assembled transcript nucleotides were found in contigs that were at least of Nx length.) **c** ExN50 value at different expression percentiles. For ExN50, the N50 statistic was computed at percentile x, for only the top most highly expressed transcripts that represent x% of the total normalized expression data. Expression values were measured using eXpress

Trinity tended to predict longer isoforms and more genes and transcripts, while many are split transcripts (Fig. 4a; Supplementary Figs. 16 and 17). Oases consistently yielded the highest N10 through N50 values for all samples (Fig. 4b; Supplementary Fig. 18) indicating its superiority in detecting long isoforms. Next, ExN50 was used to measure N50 for the top most highly expressed transcripts at different levels (Fig. 4c; Supplementary Fig. 19). SOAPdenovo-Trans had a peak at highly expressed genes (small x percentiles), indicating its strong tendency to detect highly expressed isoforms. On the other hand, Oases achieved its peak in the far right of the plot, i.e., after including most of the genes, indicating its effectiveness in capturing low-expression genes. SOAPdenovo-Trans had, on average, 3% higher alignment quality (percent identity to reference) than Trinity and Oases (Supplementary Fig. 20).

Comparing the reconstructed transcript with the reference annotation revealed that SOAPdenovo-Trans and Trinity had highest intron level precision and sensitivity, respectively (Supplementary Fig. 21a). For intron-chain level accuracy, Oases and Trinity outperform SOAPdenovo-Trans (Supplementary Fig. 21b). With lower memory and computation requirements, SOAPdenovo-Trans yielded the most efficient performance regardless of read normalization (Supplementary Table 5).

### Isoform detection using long reads

The median length of human transcripts (in GENCODE v19 annotation) is 783 bp, much longer than what current NGS technologies can deliver. However, long-read sequencing platforms can easily generate reads that completely span most transcripts. On the hESC sample, for example, the median length of the raw PacBio subreads is 1164 bp, which is long enough to cover most transcripts (64%). Thus, long-read technologies can facilitate exact isoform discovery without needing exon–exon junction prediction or assembly.

### Long-read error correction

Despite easier transcriptome reconstruction, long TGS reads usually have a relatively high error rate that hinders their direct application for RNA-seq analysis. Several tools aim to reduce these error rates including LSC^31^, proovread^32^, LoRDEC^33^, and PBcR^34, 35^. Here we analysed the hybrid long-read error correction tools LSC and LoRDEC.

LSC was ~100× slower than LoRDEC (Supplementary Table 6). Using LSC and LoRDEC on MCF7 data led to a 6.8% and 4.6% respective increase in the number of mapped reads compared to the raw long reads. However, reads corrected by LSC were of lower quality when compared to LoRDEC. Since LoRDEC had better accuracy and speed, it was the preferred error correction tool for downstream analysis. For NA12878, since the long reads were circular-consensus sequences (CCS) no correction was needed. New technologies such as SLR-RNA-seq^30^ can provide synthetic long reads at low error rates through assembly.

### Long-read isoform detection

Given the error-corrected or error-free long-read sequences, full-length isoforms can be directly predicted^12, 36^. However, to improve the prediction, IDP’s hybrid approach can be used to employ the alignment and junctions predicted from short reads in long-read isoform detection. We used GMAP^26^ and STARlong long-read alignments as inputs to IDP. On average, GMAP aligned 28% more reads than STARlong (Supplementary Table 7). As an alternative to IDP, the long-read-only isoform predictions from PacBio’s Iso-Seq pipeline on MCF7 sample were also assessed.

On different samples, long-read-based techniques IDP and Iso-Seq predicted many novel isoforms or known reference transcripts that were not detected by any short-read-based technique (Supplementary Fig. 22). Statistical analysis on the set of the transcripts that were only predicted by long-read or short-read techniques revealed significantly different length distributions. Transcripts predicted only by IDP had a wide range of lengths (up to 10,000 bp) while most of those predicted only by Iso-Seq algorithm were of lengths between 1,000 and 4,000 bp.

In terms of speed, STARlong was 68× faster than GMAP (Supplementary Table 8), while IDP took around 170 CPU hours to process each sample.

### Transcript quantification

*Alignment-based transcript quantification*. Traditional expression analysis aligns reads to the reference genome or transcriptome followed by an estimation of transcript abundances. If the goal is to measure the abundances of novel isoforms in addition to the known ones, then transcriptome assemblers like Cufflinks and StringTie can be employed. When a transcriptomic reference is available, reads can be directly aligned to it followed by an abundance estimation step using tools like RSEM^37^ and eXpress^18^.

*Alignment-free transcript quantification.* Alignment-free quantification approaches assign reads directly to transcripts, which is computationally cheaper than spliced alignment. Several such approaches have been proposed, such as Sailfish^38^, Salmon^39^, quasi-mapping^40^, and kallisto^41^ that aim at resolving which isoform could have generated each read, or finding the partial alignment of reads to the transcriptome.

Here we compared the performance of the genome-alignment-based tools, StringTie and Cufflinks (using different aligners), transcriptome-alignment-based tools, eXpress and Salmon-Aln, the alignment-free tools kallisto, Sailfish (with quasi-mapping), Salmon-SMEM, and Salmon-Quasi, and the long-read-based technique IDP (using different short-read and long-read aligners).

**Fig. 5:**
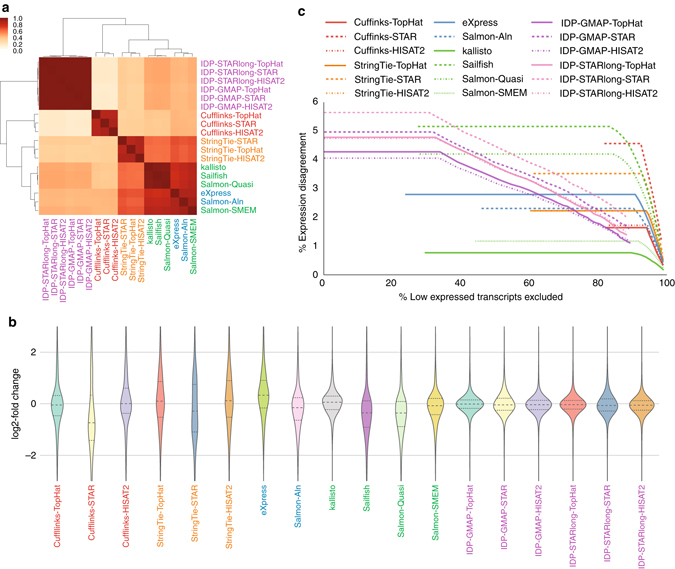
Performance of transcript abundance estimators. **a** Clustering of different schemes based on the Spearman rank correlation of their log expressions on NA12878. **b** Distribution of log2-fold change of expressions between MCF7-100 and MCF7-300 samples. For each method, dashed line represents the mean of the distribution and the dotted lines represents the quartiles. **c** Percentage of expression disagreement between MCF7-100 and MCF7-300 samples when low-expressed transcripts are discarded with different thresholds

Clustering different quantification approaches based on the Spearman rank correlation between their log-scaled expression values suggested that schemes with similar approaches clustered well together (Fig. 5a; Supplementary Figs. 23 and 24). The alignment-free tools also clustered closer to StringTie than Cufflinks. Salmon-SMEM consistently had results similar to transcriptome-alignment-based techniques. Given the much faster speed, this put Salmon-SMEM ahead of eXpress and Salmon-Aln. Combinations involving IDP also clustered together with less similarity to other combinations, especially the ones involving Cufflinks (Fig. 5a).

The two alignment-free tools kallisto and Salmon-SMEM had the most consistent predictions across MCF7-100 and MCF7-300 samples among the short-read-based techniques, which was consistent with results in ref. ^5^ (Figs. 5b, c). Similar observations were made when comparing replicate samples in the SEQC database (Supplementary Figs. 25–27) reflecting that alignment-free tools yielded the least sample-specific and read length bias in their abundance estimation. IDP showed high consistency across both MCF7-100 and MCF7-300 short-read data sets (Fig. 5b), especially when under-expressed genes were excluded (Fig. 5c). HISAT2 seemed most effective in predicting consistent results when used as the short-read aligner (Fig. 5c).

In general, the alignment-free tools were very efficient (Supplementary Table 9), while StringTie with efficient aligners like HISAT2 was the most efficient alignment-based approach (an order of magnitude slower than alignment-free tools).

Previous studies have shown that the quantification approach used has a more prominent role in the accuracy of abundance estimation than the choice of aligner^4, 5^. Although confirming this characteristic (Fig. 5a), our results contrast different aligners more clearly (Fig. 5c) depicting the superiority of HISAT2 and TopHat over STAR on challenging samples.

### Differential expression

Identifying the set of differentially expressed genes across different samples and conditions is an important goal in many RNA-seq studies. Multiple approaches exist to accurately detect differentially expressed genes, which include count-based techniques like DESeq2^42^, limma^43^, and edgeR^44^, assembly-based techniques like Cuffdiff^45^ and Ballgown^46^, or sleuth^47^ that perform differential analysis on alignment-free quantifications.

Here we analysed the performance of these schemes. Count-based techniques were evaluated when coupled with different alignment-based, alignment-free, or transcriptome reconstruction tools. Ballgown was coupled with StringTie or Cufflinks using different aligners. Cuffdiff was used along with Cufflinks. Sleuth was compared with quantifications from kallisto, Sailfish, or Salmon.

**Fig. 6:**
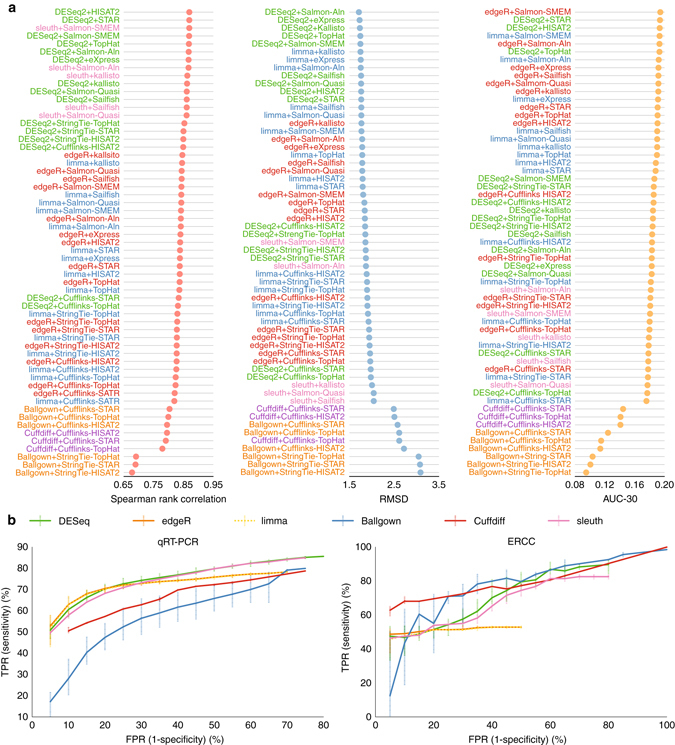
Performance of differential gene expressions analysis tools on SEQC-A vs. SEQC-B samples. **a** Spearman rank correlation, root-mean-score-deviation (RMSD), and AUC-30 scores for qPCR measured genes. Spearman rank correlation and RMSD scores are measured between the log2-fold change of the qRT-PCR and RNA-seq tools. AUC-30 score represents the area under the ROC curve up to the false positive rate of 30%. **b** ROC analysis of qRT-PCR measured genes (*left*) and ERCC (*right*) genes. For each differential analysis tool the plot reflects average performance when different alignment-based and alignment-free tools are used for abundance estimation and error bar shows the maximum and minimum variations. Results for each tool combination are shown in Supplementary Figs. 30 and 35

First, the tools were compared in detecting the set of differentially expressed genes from the 1001 genes in the SEQC samples (SEQC-A vs. SEQC-B, and SEQC-C vs. SEQC-D) with known expression changes measured by quantitative PCR with reverse transcription (qRT-PCR) (Fig. 6; Supplementary Figs. 28–34). On average, DESeq2 outperformed other techniques with different choices of quantification schemes, while sleuth, edgeR and limma had slightly lower performance, which confirms the results in ref. ^7^. Cuffdiff and Ballgown were consistently less accurate than raw-count-based techniques for all accuracy measures. Salmon-SMEM, Salmon-Aln, kallisto, and eXpress made the most accurate combination with raw-count-based schemes. In terms of the area under the ROC curve up to the FP rate of 30% (AUC-30) measure, edgeR outperformed other techniques and increased its superiority if the true positive log2-fold change cutoff was increased more than 0.5 (Supplementary Figs. 32–34).

As another accuracy measure, different schemes were compared in predicting the expression variations in 92 External RNA Control Consortium (ERCC) spike-in genes in the SEQC data set (Fig. 6b; Supplementary Figs. 29, 35–38). In terms of the Spearman rank correlation measure, edgeR and limma were significantly outperfromed by other schemes. For both Spearman rank correlation and RMSD measures, DESeq2 still yielded the best performance, while sleuth outperformed edgeR and limma. On AUC-30 measure, however, Cufflinks with Ballgown outperformed other techniques. On the ERCC genes, in general, using the assembled transcripts from StringTie and Cufflinks as the input annotation to count-based methods improved the accuracy (Fig. 6b; Supplementary Figs. 37 and 38).

The count-based tools were more efficient than the assembly-based approaches especially when used on transcriptome-alignment or alignment-free approaches (Supplementary Table 10). Being four to five times slower than Ballgown, Cuffdiff was the slowest tool.

Unlike other studies^7^, our work not only compared various differential analysis tools, but also studied the impact of different alignment-based and alignment-free approaches on the accuracy of differential analysis. Overall, alignment-free techniques such as Salmon or kallisto were observed to be capable of delivering high-quality predictions.

### RNA-seq variation analysis

In addition to expression level information, RNA-seq data can be used to identify important genomic and transcriptomic variations.

### Variant calling

Detecting genomic and transcriptomic variants is critical in understanding the regulatory and disease-associated variants that may affect gene expression. Approaches commonly used for genomic variant calling, such as SAMtools mpileup^48^ and GATK’s HaplotypeCaller^49^, can be applied to RNA-seq data. To account for RNA-seq data, GATK applies RNA-specific filtering steps for handling RNA splicing. Here the performance of the GATK best practices workflow for RNA-seq data was compared against SAMtools, using alignments computed by TopHat, STAR, HISAT2, or RASER^50^. RASER is an aligner specifically designed for accurate variant calling by aiming at reducing the false-positive rate in mapping of RNA-seq reads.

**Fig. 7:**
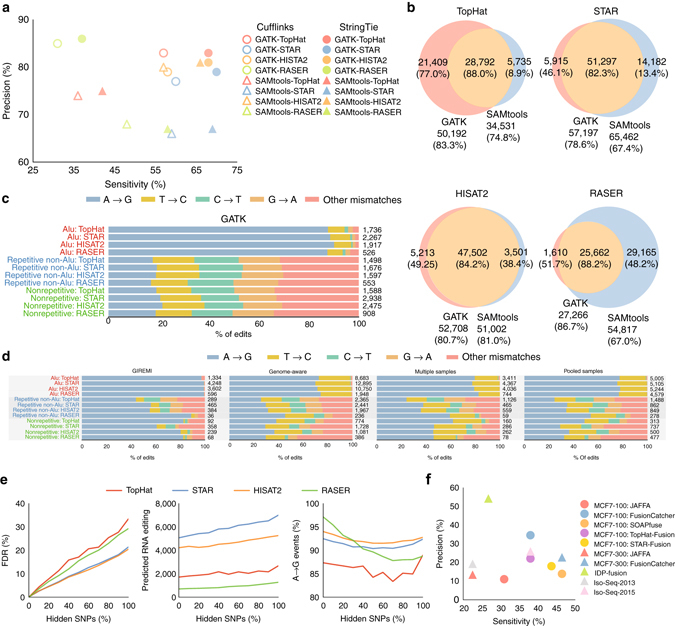
Performance of different variant calling (**a**–**c**) RNA editing (**d**, **e**) and RNA fusion (**f**) detection approaches. **a** Accuracy of detecting NA12878 high-confidence calls in NIST gold standard. The analysis is restricted to the expressed exons identified by Cufflinks or StringTie. **b** Overlap between variants predicted by GATK and SAMtools. **c** Distribution of the predicted mismatch types by GATK that are missed in NIST HC calls (in StringTie’s expressed exons) in different genomic regions. **d** Distribution of RNA editing events detected in different genomic regions for NA12878. For multiple-samples scheme, final editing sites include the rare variants in NA12878 that are supported by at least 3 out of 12 short-read samples in our analysis. For pooled-samples scheme, final editing sites include the rare variants in NA12878 that are supported by at least 20 reads in the pooled alignment. **e** Measurement of RNA editing detection accuracy when some portion of SNPs are hidden from GIREMI. FDR represent proportion of predicted edits that are among high-confidence genomic variants in NA12878. **f** Performance of different RNA fusion detection schemes on MCF-7 sample

The accuracy was assessed on NA12878 using the NIST high-confidence (HC) calls^51^ as the gold standard (Fig. 7a). We restricted the gold standard to the expressed regions since only a subset of reference transcripts in the high-confidence regions would be expressed.

Unlike TopHat and STAR, when HISAT2 alignments were used, GATK and SAMtools had similar performance, suggesting that GATK’s more complex approach could be avoided if an accurate aligner was used (Fig. 7a). With RASER, GATK’s precision increased by 19%, while the sensitivity was reduced by 21%, possibly due to strong filtering used in GATK. Since RASER was designed for fewer false-positive calls, it yielded the most precise calls but was the least sensitive when used in combination with GATK. On the other hand, STAR had the highest sensitivity, but least precision. Calls made only by GATK were always more precise than SAMtools private calls (Fig. 7b). As further confirmation of variant-calling accuracy, 93–97% of TP calls had genotypes consistent with the NIST HC predictions (Supplementary Fig. 39).

Approximately 95% of GATK’s FP calls, which were missed in NIST HC variant set, were A-to-G or T-to-C variants, the dominant RNA-editing substitutions seen in non-strand-specific sequences in Alu repeat regions of the genome^52, 53^ (Fig. 7c). On non-Alu regions, around 35% of the calls were A-to-G or T-to-C mismatches and another 33% of the FP calls were C-to-T or G-to-A known as the other (much less) pervasive RNA-editing event^54^. This further reflects the accuracy of variants called using GATK, especially in the Alu repeat regions of the genome. The same analysis for SAMtools revealed that when HISAT2 or RASER was used to align, a significant proportion of FPs followed the canonical distribution of RNA-editing substitution events (Supplementary Fig. 40).

STAR had higher number of common variants across two short-read MCF7 samples and SEQC replicates, but slightly lower validation rate against the dbSNP database (Supplementary Fig. 41). On average, TopHat and HISAT2 respectively had 10% and 7% more validation rate on the private calls.

In general, GATK and samtools have similar execution times over different samples (Supplementary Table 11).

### RNA-editing detection

RNA editing is the post-transcriptional modification of RNA sequences that can impact the functional regulation of the sequences and their expression levels^52, 53^. Several approaches have been proposed to detect RNA-editing events using RNA-seq data^52, 53, 55^. The most common approach is to identify the RNA variations that are different from the matched genomic sequences in the DNA molecule^52^. This approach (genome-aware) requires the availability of both RNA and DNA sequences of the underlying sample. Additionally, careful analysis of read mapping and variant calling is critical to effectively discriminate the RNA edits from sequencing errors^52^. GIREMI^53^ is a genome-independent approach that can predict RNA edits for a single RNA-seq data set using allelic linkage between SNVs. Two other alternative genome-independent approaches^55^ employ multiple RNA-seq data sets to increase the confidence of finding individual sites. In one approach (multiple samples), RNA edits are identified as a set of rare variants that occurs in multiple samples. In another approach (pooled samples), the RNA variants are called on the pooled alignments of all samples and RNA edits are identified assuming they occur more frequently than rare SNPs. Here, these approaches were compared when different aligners were used with GATK.

First, adenosine to inosine (A-to-I) editing was the dominant event type by different schemes (Fig. 7d; Supplementary Figs. 42 and 43). In Alu repeats, GIREMI yields 99% A-to-G edits when HISAT2, STAR, or RASER was used. As expected, RASER, despite its lower sensitivity, was more specific in detecting A-to-G edits in all regions. The genome-aware, multiple-samples, and pooled-samples methods were capable of detecting more editing sites but high prevalence of T-to-C mismatches was observed.

The percentage of A-to-G and T-to-C edits vs. increasing minimum RNA-editing levels are compared in Supplementary Fig. 44. TopHat in combination with GIREMI outperformed other techniques only for high editing levels, while RASER had consistent superiority over other alignment techniques for different editing levels in both GIREMI and genome-aware schemes.

Next, the false discovery rate (FDR) of GIREMI was compared on NA12878 when a varying proportion of the NIST high-confidence genomic variants were hidden from GIREMI (Fig. 7e). FDR was then defined as the proportion of reported RNA editing among high-confidence genomic variants in NA12878. STAR and HISAT2 had lower FDRs while they predicted more edits with higher rate of A-to-G mismatches. RASER showed higher sensitivity to the existence of genomic variants in the input SNV set.

In Alu repeats, all aligners get a higher rate of A-to-G edits with more supporting samples/reads, while in other regions this effect is less prominent especially for TopHat and STAR (Supplementary Figs. 45 and 46).

Given the genomic and transcriptomic variants, the genome-aware approach is ~10× faster than GIREMI, while the multiple-samples and pooled-samples methods are more computationally expensive since they need analysis on multiple data sets. (Supplementary Table 12).

### RNA fusion detection

Another important application of RNA-seq is to detect fusion genes, which are abnormal genes produced by the concatenation of two separate genes arising from chromosomal translocations, or trans-splicing events^6^. Fusion genes play a critical role in investigating causes and development of various cancer types^56^. RNA-seq has been shown as a valuable source for detecting fusion genes^6^. Several tools have been proposed to identify fusion from transcriptomic data such as JAFFA^57^, STAR-Fusion^14^, TopHat-Fusion^58^, FusionCatcher^59^, and SOAPfuse^60^. In addition to these short-read-based techniques, IDP-fusion^61^, and Iso-Seq^27^ methods can analyse long-read RNA-seq data to identify fused genes.

Here we assessed these approaches in detecting the 71 validated gene fusions in the MCF-7 breast cancer cell-line^61^. Iso-Seq algorithm predictions were obtained from ref. ^11^. Among the short-read-based techniques, FusionCatcher yielded most sensitive and precise predictions, and SOAPfuse also showed higher sensitivity, which was consistent with results in ref. ^6^. In addition, we found that the long-read-based approach IDP fusion provided the highest precision (Fig. 7f).

STAR-Fusion is the fastest approach (more than 10× faster than other methods), while FusionCatcher and TopHat-Fusions have higher computation demands (Supplementary Table 13).

### Run-time analysis

The runtimes of different algorithms across different steps, are shown in Supplementary Tables 3–6 and 8–13. The overall runtimes of different approaches are also summarized in Supplementary Figs. 47 and 48. Alignment-free approaches were the fastest while the StringTie-HISAT2 combination, which was an order of magnitude slower, was the fastest alignment-based alternative. The Tuxedo protocol (Cufflinks-TopHat) and long-read-based approaches were two orders of magnitude slower than StringTie-HISAT2.

### A high-accuracy pipeline

**Fig. 8:**
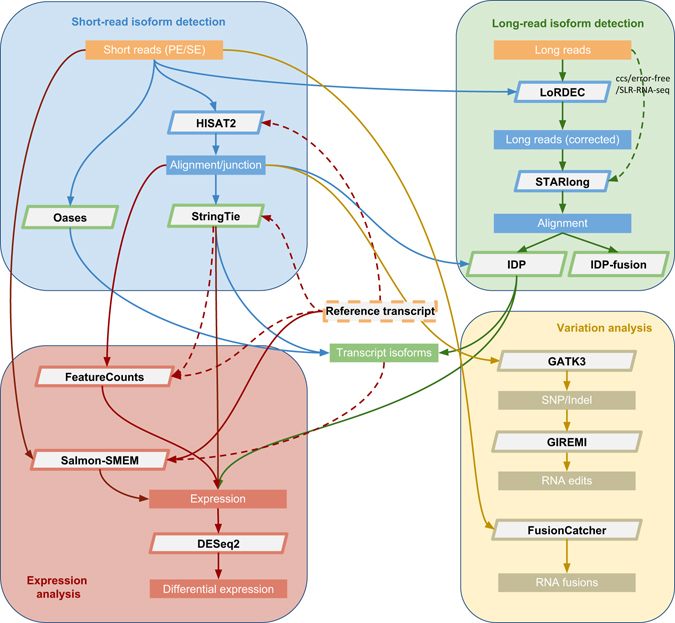
The current RNACocktail computational pipeline. The pipeline is composed of high-accuracy tools in each step for general-purpose RNA-seq analysis

Although no single tool was the best under all conditions, based on the overall performance of the analysed RNA-seq analysis tools, we propose the RNACocktail pipeline, composed of high accuracy tools in each step, for general-purpose RNA-seq analysis (Fig. 8). As shown through the previous comprehensive analysis, the currently widely used Tuxedo protocol^62^ of Cufflinks-TopHat was usually outperformed by other unconventional alignment-based (e.g., StringTie-HISAT2) or alignment-free (e.g., Salmon-SMEM) approaches for multiple accuracy metrics as well as computational cost.

To demonstrate the performance enhancement in our proposed pipeline, the set of overexpressed genes in the MCF7 and hESC samples relative to the normal NA12878 samples were identified using both our pipeline and the Tuxedo approach. For the top 10 overexpressed genes, functional enrichment analysis was conducted using the ToppGene Suite^63^ (Supplementary Table 14 and Supplementary Data 1–3). On MCF7-100 and MCF7-300 samples, Cufflinks-TopHat prediction sets were not enriched in any MCF7 or breast cancer-related gene expression study, while StringTie-HISAT2’s and Salmon-SMEM’s top overexpressed genes were highly enriched in many MCF7 and breast cancer cell line-related gene sets (Supplementary Data 1 and 2). Similar observations were made for the hESC sample, where StringTie-HISAT2’s and Salmon-SMEM’s top overexpressed genes were highly enriched in several human embryonic stem cell-related gene sets while the gene sets in which top Cufflinks-TopHat predictions were enriched were mostly unrelated to the hESC sample (Supplementary Data 3).

In addition to being supported by extensive assessment across multiple RNA-seq steps, the proposed pipeline is also more comprehensive than other pipelines such as Galaxy^64^ and Grape^65^. These pipelines are limited to a few RNA-seq steps and miss other key steps such as de novo assembly, variant calling, RNA-editing detection, and long-reads RNA-seq analysis^64^, or have ignored recently developed tools in the pipeline^65^.

## Discussion

As demonstrated through our comprehensive analysis of different steps of RNA-seq analysis, the choice of tools and computational approaches had a large impact on the accuracy and runtime of the analysis. HISAT2 yielded the fastest and the most precise spliced alignment, although less sensitive than STAR. StringTie outperformed Cufflinks both in speed and accuracy in most settings. While missing some single-exon isoforms, long-read approaches like IDP and Iso-Seq can identify many novel multi-exon transcripts missed by the short-read techniques. Oases, in general, outperformed other de novo assemblers and can offer unbiased isoform predictions. Alignment-free tools like Salmon-SMEM and kallisto yielded the most consistent and accurate quantifications, and, thus, can serve as the most accurate yet computationally cheap solutions if isoform discovery is not important. DESeq2 and edgeR provided the most accurate differential analysis especially when coupled with alignment-free techniques. GATK was an accurate variant caller for RNA-seq in combination with different alignment tools, while using HISAT2 alignments can make SAMtools predictions as good. GIREMI yielded accurate genome-independent prediction of RNA-editing sites, especially when used on HISAT2 or STAR aligners. Finally, long-read methods like IDP-fusion can precisely predict RNA fusion events, while short-read schemes such as FusionCatcher or SOAPfuse offer higher sensitivity. In general, the best overall approaches may have sub-optimal performance for a specific data set with respect to a specific measure. For instance, while HISAT2 and StringTie have higher overall accuracy and speed for alignment and transcriptome reconstruction steps, the combination of STAR and StringTie has higher sensitivity on MCF7-300 (Fig. 3a). Similarly, for differential analysis of SEQC-C vs. SEQC-D samples on ERCC genes, DESeq2 + StringTie + STAR had higher Spearman rank correlation than DESeq2 + StringTie + HISAT2 (Supplementary Fig. 38).

Detailed analysis of the set of highly overexpressed genes in hESC and MCF7 samples revealed that more recent techniques are much better than the standard Tuxedo protocol. For instance, considering the 89 genes listed as the stemness signature^66^, which are the set of upregulated genes common to six human embryonic stem cell lines, StringTie-HISAT2 and Salmon-SMEM approaches respectively had 6 and 4 out of 10 of their top hESC genes appearing in this list (with respective Bonferroni-corrected *p* values of 3.67×10^−8^and 1.32×10^−6^) while Cufflinks-TopHat approach had none of its top 10 genes in this list. The six top genes found by StringTie-HISAT2 in this list were *TDGF1*, *CRABP1*, *SFRP2*, *GJA1*, *GAL*, and *LIN28A* with functional roles in various embryonic development activities (Supplementary Table 15). Similarly, on the MCF7 sample, the StringTie-HISAT2 approach predicted important breast cancer-related genes including *TFF1*, *AGR2*, *TFF3*, *SERPINA3*, *SLC7A2*, *DSCAM-AS1*, *SEMA3C*, *KRT19*, and *KRT8* among its top 10 upregulated genes (Supplementary Table 16).

Although some long-read technologies may have a higher experimental cost, they can yield better predictions as their reads can easily span multi-exon isoforms. For instance, among the genes predicted only using long reads, there were 3, 4, and 20 genes, respectively in NA12878, MCF7, and hESC samples, which fell in the highly polymorphic human major histocompatibility complex (MHC) genomic region on chromosome 6. MHC encodes the human leukocyte antigens (HLA) genes and many other genes with important roles in the regulation of the immune system as well as in some fundamental cellular processes and known to be associated with more than 100 diseases, including common ones such as diabetes, rheumatoid arthritis, psoriasis, asthma, and various autoimmune disorders^67^. *HLA-DQA1* and *HLA-DPA1* genes, for instance, provide instructions for making proteins that are present on the surface of certain immune system cells, and were only detected by IDP, respectively, in hESC and NA12878 samples (Supplementary Figs. 49 and 50). *AGER* is also a non-HLA gene in MHC region that is a member of the immunoglobulin superfamily of receptors^68^ identified by IDP in the MCF7 sample (Supplementary Fig. 51). In all these cases, several long reads fully span the given isoforms along multiple exons. To see the potential of long reads in detecting very long isoforms with many exons, IDP’s private predictions for a 12-exon 4491-bp isoform in *MLH3* gene (on NA12878) and a 19-exon 4784-bp isoform in *MYO9A* gene (on MCF) were explored (Supplementary Figs. 52 and 53). IDP successfully predicted these isoforms by taking advantage of several long reads spanning the transcripts.

To conclude, our comprehensive assessment with detailed investigation at each analysis step not only clearly outlines the current state of the RNA-seq analysis and highlights algorithm issues that warrant the attention of researchers, but also leads to a broad-spectrum analysis protocol that can enable researchers to unleash the full power of RNA-seq. We envision that our approach will facilitate researchers in gaining better and more comprehensive biological insights from their transcriptomic data, as exemplified by the results of our pipeline, which is only one possible instantiation of the comprehensive protocol.

## Methods

### Data sets

Supplementary Table 1 summarizes the data sets used in this study. We describe below the details of the individual data sets.

### MCF-7

MCF-7 is one of the most commonly used breast cancer cell lines. We used both short-read and long-read data for this sample in our analyses.

*Illumina MiSeq/HiSeq sequencing*. Prior to sequencing, the MCF7 total RNA was assessed for fragmentation and quality using Agilent Bioanalysis and Qubit, respectively. RNA-seq libraries were prepared after ribosomal depletion, using Epicentre RiboZero commercial reagents. Following cDNA preparation, Covaris shearing was conducted to an insert size of ~600 bp as assessed by Agilent Bioanalysis using standard Illumina adapters and PCR cycle conditions for sequencing on the Illumina MiSeq instrument. 2 × 300 bp paired-end sequencing was then conducted across two Illumina MiSeq flow cell lanes using version 3 commercial kits to assure the longest read length possible. An additional Illumina HiSeq lane was completed on the Illumina HiSeq 2500 with 2 × 100 bp paired-end reads totaling ~127 M additional reads with ~83% over QV30. All data were then merged for analysis.

*PacBio sequencing*. The PacBio sequences for MCF-7 were obtained from the PacBio’s 2013 release of the MCF7 transcriptome data (http://www.pacb.com/blog/data-release-human-mcf-7-transcriptome/).

### H1-ESC

Embryonic stem cell (ESC) lines offer a great opportunity to understand human development and disease. In this study, we used the H1-ESC cell line, which is a well-studied sample and was part of the ENCODE project.

*Illumina sequencing*. The paired-end short reads (101 bp) were generated from human embryonic stem cells (H1 cell line) on the Illumina HiSeq 1000 platform. Six replicates were collected and pooled for the analysis. The paired information was then ignored and 205,044,801 single-end reads were used.

*PacBio sequencing.* The PacBio sequences for H1-ESC were obtained from the data used in the original IDP paper^12^. It can be found at Gene Expression Omnibus (GEO) database, http://www.ncbi.nlm.nih.gov/geo (accession no. GSE51861). For the PacBio raw sequences, it will be provided upon contacting Kin Fai Au (kinfai-au@uiowa.edu).

### NA12878

HapMap normal human sample NA12878 is a very well-studied sample used for assessing both germline DNA and RNA analysis. Since high-confidence genomic variants have been made available for this sample, it offered a great opportunity for assessing RNA-seq variant-calling. PacBio and Illumina sequences were obtained from an earlier study^10^. It can be found in the NCBI Sequence Read Archive (accession no. SRP036136). Further data are available at http://stanford.edu/~htilgner/2014_PNAS_paper/utahTrio.index.html. The data set includes 115.4 million 101-bp paired-end reads from an Illumina Hi-Seq 2000 sequencer and 715,902 CCS long reads from a Pacific Biosciences real-time sequencer RTII. The mean length of the long reads was 1,188 bp with a maximum of 6 kbp.

### SEQC

We considered four sets of two replicates each from the Sequencing Quality Control Consortium^8^, one set (composed of replicates SEQC-A1 and SEQC-A2) corresponding to the Universal Human Reference RNA (UHRR), another (composed of replicates SEQC-B1 and SEQC-B2) corresponding to the Human Brain Reference RNA, and two others (composed of replicates SEQC-C1, SEQC-C2, and replicates SEQC-D1, SEQC-D2) were created by mixing the well-characterized samples A and B in 3:1 and 1:3 ratios. These samples also have 92 spiked-in RNA controls from the ERCC to assist in evaluation of differential expression. Each of the replicate was sequenced using Illumina Hiseq 2000 to generate, on average, 110 million paired-end reads of 101-bp length each. For this study, we chose sequences from the ILM2 site, which is one of the six sites providing the Illumina sequences.

### Transcript annotation

The Ensembl v73^25^ annotation was used as the guiding reference transcriptome annotation for alignment, transcriptome reconstruction, and quantification tasks. The more comprehensive annotation, GENCODE v19^28^, was used as the reference transcriptome annotation for evaluation of the predictions. Since GENCODE is more comprehensive and includes many transcripts missing in Ensembl, we can measure the performance of different tools in predicting novel isoforms that they have not seen during prediction.

### RNA-seq analysis tools

The list of RNA-seq tools, their versions, and the command line options used in the analysis are listed in Supplementary Table 2. All tools except for de novo assembly tools were run on a dual-hexcore X5675 Intel Xeon node with 96 GB memory. The de novo assemblers were run on an AWS R3.8xlarge instance (16 virtual processors with 244 GB memory) due to larger memory requirements.

### Junction prediction accuracy measure

The list of junctions in the database for expressed sequence tags (dbEST) (dated 12 October 2015) was obtained and used for assessment of the splicing junctions predicted by different alignment tools. For a more precise comparison, only the reliable set of junctions that were supported by at least two EST entries was considered in the evaluations. For SEQC samples, the predictions were validated on reliable junctions called in the SEQC database^8^ across multiple platforms. In the SEQC project, three platforms (Illumina, Roche 454, and SOLiD) were examined in the project at 12 different sites. Reliable SEQC junction set consists of junctions supported by at least two different platforms or by Illumina sequencers at all sites.

### Read mapping evaluation

The number of mapping instances of each sequencing read was detected using the NH tag in the alignment file. The number of soft-clipped bases was obtained from the alignment CIGAR string. The number of mismatches was detected using the NM tag.

### Transcriptome reconstruction evaluation

The cuffcompare tool in the Cufflinks package was used to evaluate the predicted transcripts against the GENCODE reference annotation. Cuffcompare measures the accuracy at the base, exon, intron, intron-chain, transcript, and gene (loci) levels. It was also used to find the set of merged transcripts obtained from IDP and Cufflinks or StringTie. To assess the performance of different techniques in predicting novel isoforms, we collected the set of reference multi-exon transcripts in GENCODE that were missing in the Ensembl reference annotation, which was used during isoform detection. To find such a set we used cuffcompare to compare GENCODE against Ensembl and any isoform which was reported by either of “u”, “i”, or “j” tags in the output “.tracking” file was considered as a novel isoform in GENCODE. This set included 3681 isoforms across 2201 genes with an average 6.3 exons per isoform. Different assemblers were then assessed in predicting these isoforms using cuffcompare and the number of predictions that matched (tagged “=”) or were contained (tagged as “c”) in any of the the novel isoforms were reported. To identify what extent of FP calls (with respect to GENCODE v19) are still assumed to be FP in the latest GENCODE release v25, we compared different techniques in predicting 9221 novel isoforms present in GENCODE v25 but missing in the ENCODE v19 annotation (Supplementary Fig. 15).

### De novo transcriptome assembly evaluation

Trimmomatic^69^ was used to discard low-quality reads and trim poor-quality bases before running de novo assembly. Trinity and Oases ran out of memory even on a machine with 244 GB memory. Therefore, Trinity’s in-silico read normalization was employed to reduce memory and computational requirements for all methods. Since SOAPdenovo-Trans successfully reconstructed the transcriptome on the non-normalized sequencing data for NA12878 and MCF7 paired-end sequencing samples, we also report its full results (called SOAPdenovo-Trans-ALL). The perl script TrinityStats.pl in the Trinity package was used to measure the number of predicted transcripts and isoforms, as well as contig N10 to N50 values. The Nx contig length statistic reflects that at least x% of the assembled transcript nucleotides were found in contigs that were at least of Nx length. To incorporate expression levels in the evaluation of assembly quality, the ExN50 measure was used as an alternative to the Nx statistic. For ExN50, the N50 statistic was computed for only the top most highly expressed transcripts that represent x% of the total normalized expression data. The position of the maximum ExN50 value over different expression percentiles represented how well the assembler detected long isoforms even if the expression value was low. Quantification is conducted using eXpress and kallisto. The perl scripts abundance_estimates_to_matrix.pl and contig_ExN50_statistic.pl were also used to extract the expression based ExN50 measure. Expression values for the assembled transcripts were measured using eXpress or kallisto quantification tools. GMAP was used to align the assembled transcripts against the reference genome and to measure the percentage identity of the aligned transcripts for isoforms of at least 200 bp.

### Long-read error correction evaluation

To measure the performance of error corrected reads, the corrected reads were mapped to the reference transcriptome and analysed using the ectool-analysis.sh script in the LoRDEC package to compute the accuracy and Gain of the error correction. Gain is defined as (TP − FP)/(TP + FN), which measures how well the tool removes errors without introducing new ones^33^. The edit distance was also measured using the NM tag in the alignment file. Percentage edit distance was measured across all the alignments, while mean edit distance was computed for each read and averaged over all reads. For hESC, LSC’s error correction did not finish after more than 2 months of running on 24 cores, but observations were similar to MCF7 on the corrected portion of the data (Supplementary Table 5).

### Transcript abundance estimation evaluation

Transcript abundances were measured in transcripts per million for all schemes. As suggested in ref. 5, we rescaled the abundances by the median expression value of the housekeeping genes^70^. Count-based techniques DESeq2, limma, and edgeR were evaluated when coupled with TopHat, STAR, and HISAT2 alignments while their features were counted by featureCounts^71^ using either the reference transcript or merged assembled transcript. To measure the expression disagreement between the two replicates in SEQC samples and between the two short-read MCF-7 samples, at different threshold values *t*, genes expressed in both replicates at cutoff *t* (i.e., expression values in both replicates are larger than *t*) are considered as disagreeing across replicates, if the absolute log2-fold change is larger than 1. To compute the log2-fold change, a pseudocount of 0.5 is added to the expression values.

### Differential expression analysis evaluation

Tablemaker was used to provide the transcriptome predictions of Cufflinks to Ballgown^46^. To prepare the abundance estimation inputs to the count-based techniques DESeq2, edgeR, and limma, featureCounts was used with input alignments from TopHat, STAR, or HISAT2, and a guide GTF file from Ensembl annotation, or assembled Cufflinks or StringTie merged GTFs. Cuffmerge or StringTie’s merge was used to merge transcript assemblies by different techniques. The set of 1001 qRT-PCR measured and 92 ERCC genes were used as the evaluation gold set of differential expression. The Spearman rank correlation and root-mean-score-deviation (RMSD) between the predicted and known log2-fold changes were measured. The target genes with no prediction by a method were assumed to be negative calls, with log2-fold change of 0. In addition, the area under the ROC curve up to the false positive rate of 30% was also measured. For ROC analysis on qRT-PCR genes, as in ref. ^7^, all the genes with known absolute log2-fold change of more than 0.5 were assumed as the target differentially expressed genes.

### Variant calling evaluation

NIST HC calls^51^ on NA12878 were used as the gold standard genomic variants set. Accuracy was measured on all variants called in the NIST HC regions that overlap the exons in Ensembl reference annotation, in the NIST HC regions that overlap (expressed) exons identified using Cufflinks and in the NIST HC regions that overlap (expressed) exons identified using StringTie. Varsim^72^ was used to compare the predicted and known variants in a given region.

### RNA-editing evaluation

As previously mentioned, NIST genomic variants were used in the genome-aware approach. The publicly available SNPs in dbSNP database (build 138)^73^ was also used to compute the mutual information in GIREMI. To identify the mismatch type of a variant or RNA edit, as in ref. ^52^, the strands of the RNA-seq reads were extracted using the reference-annotated transcriptome as follows. For each read, the strand was extracted based on the strand of genes they were mapped to, excluding the regions with bidirectional transcription. Ensembl, GENCODE, RefSeq, UCSC, and Vega databases were used to gather a comprehensive set of transcript annotation. As in ref. ^52^, the strand annotation was extended to 1 kb upstream and downstream regions of each gene. Editing levels of RNA edits were measured as the proportion of transcripts being edited at a given position. To measure the FDR of GIREMI, varying proportions (0–100%) of the NIST HC genomic variants were hidden from GIREMI and the proportion of reported RNA editing that were in the hidden set is reported. FDR values were averaged on five independent randomized input hidden sets. For multiple-samples approach, RNA variants were identified separately for each of the samples using GATK, and the rare RNA edits where selected by excluding variants in dbSNP database. The final editing sites then include the rare variants in each sample that are supported by at least 3 out of 12 short-read samples in our analysis. For pooled-samples approach, alignments are computed separately for each of the samples, variants are called on the pooled-sample alignment, and the rare RNA edits were selected by excluding variants in dbSNP database. The final editing sites then include the rare variants that are supported by at least 20 reads in the pooled alignment.

### RNA fusion evaluation

As the target gold set, a set of 71 validated gene fusions in the MCF-7 breast cancer cell line, collected in ref. ^61^ based on seven experimental publications, and validated by either PCR or Sanger sequencing, was used. Predictions of Iso-Seq algorithm were obtained from ref. ^11^. IDP-fusion predictions were obtained from ref. ^61^.

### Top differentially expressed genes analysis

To extract the set of overexpressed genes in the MCF7 and hESC samples relative to the normal NA12878 samples, the log2-fold change between the expression values (plus a pseudocount of 0.0001) across the two samples was computed and sorted. Top 10 genes in the list were then used in ToppGene suite^63^ to identify the list of enriched expression analysis studies. Studies with Bonferroni-corrected *p* values <0.05 were reported.

### Isoforms detected only using long or short reads

The set of genes identified only by IDP or Iso-Seq algorithms was detected by comparing the predicted transcripts across all methods using cuffcompare. Transcripts, which had predictions from either of IDP-based or Iso-Seq approaches and none of the short-read-based techniques, were then extracted. Similarly, transcripts identified only by short-read-based approaches and none of the long reads were extracted. Wilcoxon rank-sum test was used on these two sets to measure the statistical significance of transcript length distributions being different for these sets. Additionally, to illustrate the capabilities of long-read technologies, we selected five transcripts that are only found using IDP. The visualization included GMAP alignment of long reads along with the IDP prediction, GENCODE annotation, common SNPs (SNPs that have a minor allele frequency of at least 1% and are mapped to a single location in the reference genome in dbSNP build 146)^73^, and the interspersed repeats and low-complexity DNA sequences.

HISEQ and MISEQ are trademarks of Illumina. All other product names and trademarks are the property of their respective owners.

### Code availability

The computational pipeline is open-sourced and available at http://bioinform.github.io/rnacocktail/.

### Data availability

The RNA-seq data that support the findings of this study have been deposited in the following public repositories. Illumina and PacBio data for NA12878 are available in the NCBI Sequence Read Archive (SRA) with accession number SRP036136. The Illumina HiSeq and MiSeq sequences for MCF-7 as well as the Illumina sequences for H1-ESC sample have been deposited in the NCBI SRA with accession number SRP103629. The PacBio sequences for MCF-7 were obtained from the PacBio’s 2013 release of the MCF7 transcriptome data, available in ref. ^11^. The PacBio sequences for H1-ESC are available in NCBI GEO database with accession number GSE51861. All SEQC data sets are available through NCBI GEO accession number GSE47792.

## Electronic supplementary material

Supplementary Information

Supplementary Data 1

Supplementary Data 2

Supplementary Data 3
