# Supplementary material for: Gaining comprehensive biological insight into the transcriptome by performing a broad-spectrum RNA-seq analysis
Source: Nat Commun. 2017 Jul 5;8:59. doi: 10.1038/s41467-017-00050-4 (PMC5498581; doi:10.1038/s41467-017-00050-4)
Supplement: Supplementary file 1 — Supplementary Information [file 41467_2017_50_MOESM1_ESM.pdf]

Supplementary Figures

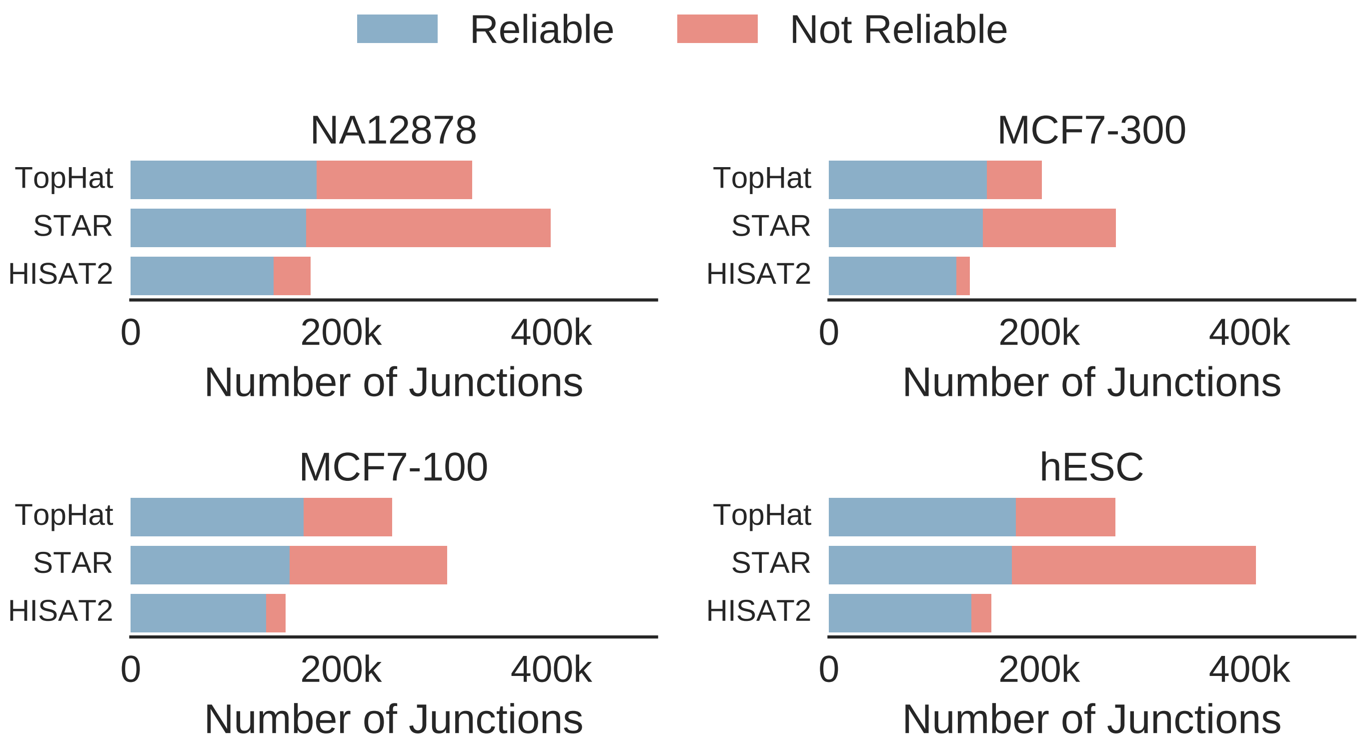

Supplementary Figure 1 | Number of splicing junctions called by each alignment scheme and its validation rate based on dbEST database.

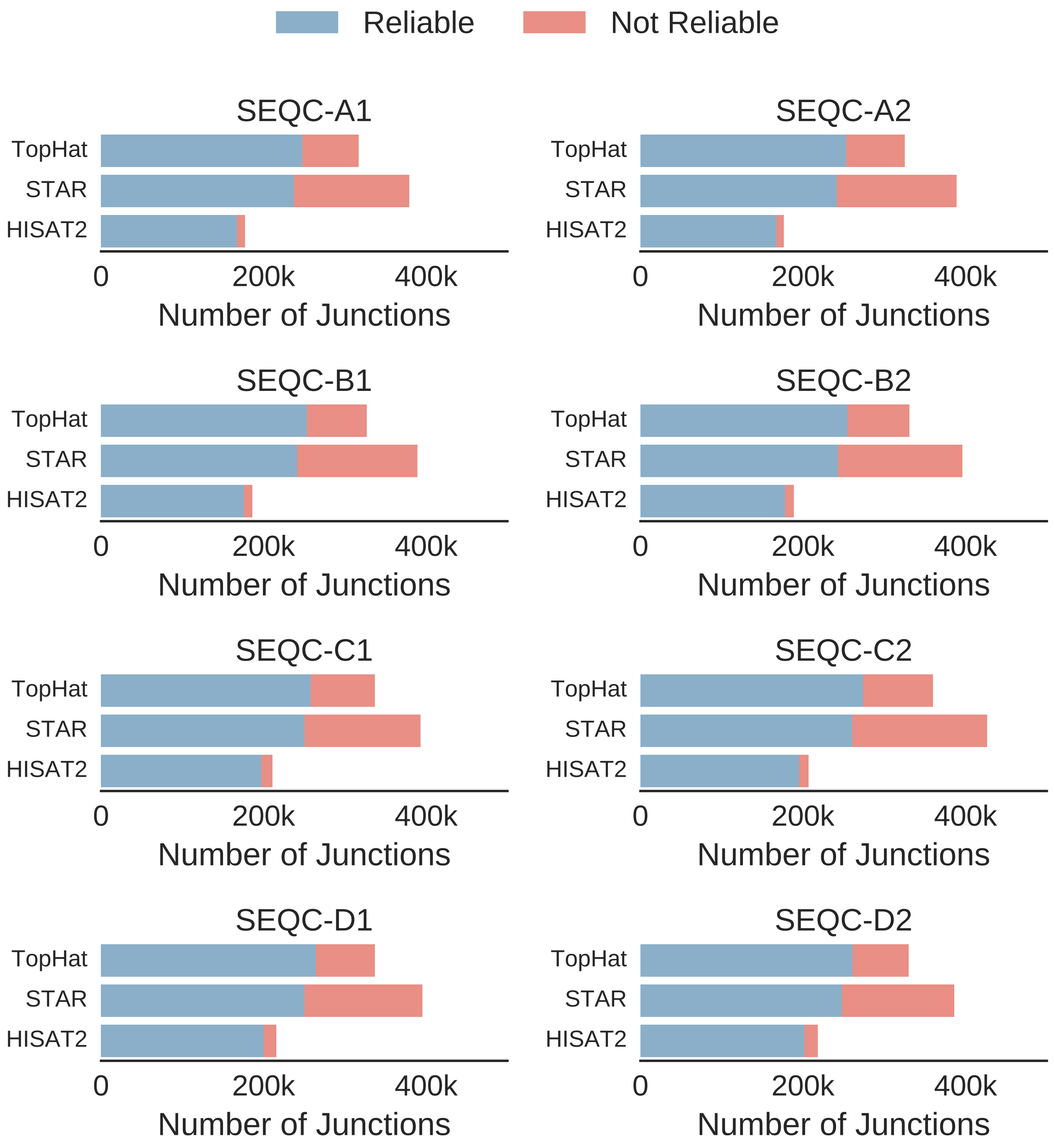

Supplementary Figure 2 | Number of splicing junctions called by each alignment scheme in the SEQC samples and its validation rate based on reliable junctions called in the SEQC database across multiple platforms. Reliable SEQC junction set consists of junctions supported by at least two different platforms or by Illumina sequencers at all sites.

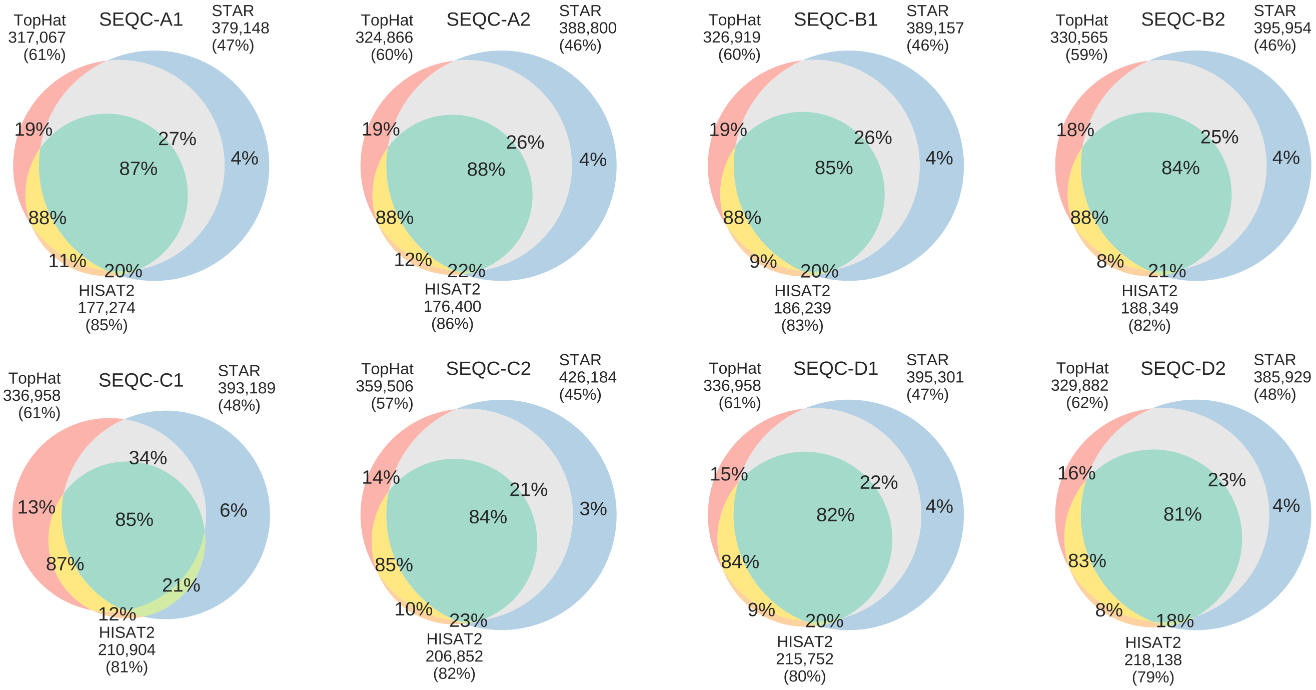

Supplementary Figure 3 | Performance of different alignment schemes in detecting splicing junctions on SEQC samples and their validation rate on reliable junctions called in the SEQC database across multiple platforms. Reliable SEQC junction set consists of junctions supported by at least two different platforms or by Illumina sequencers at all sites. The sizes of the circles reflect the number of junctions called by each scheme. For each tool, the number of junctions called and the validation rates (in parentheses) are shown. Validation rates for each subset of junctions are also shown on the Venn diagram.

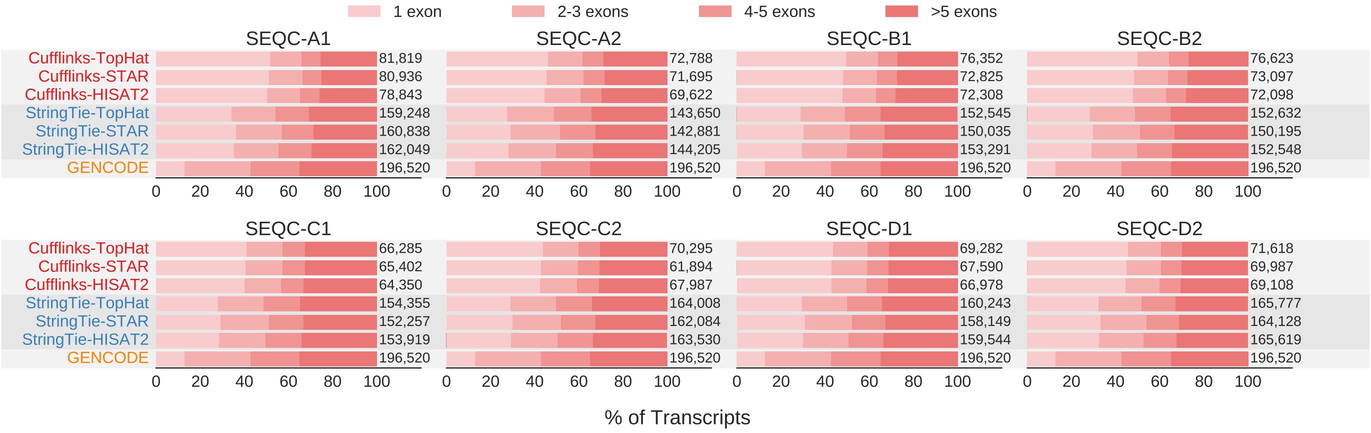

Supplementary Figure 4 | Distribution of number of exons per transcripts for different transcriptome reconstruction algorithms on SEQC samples.

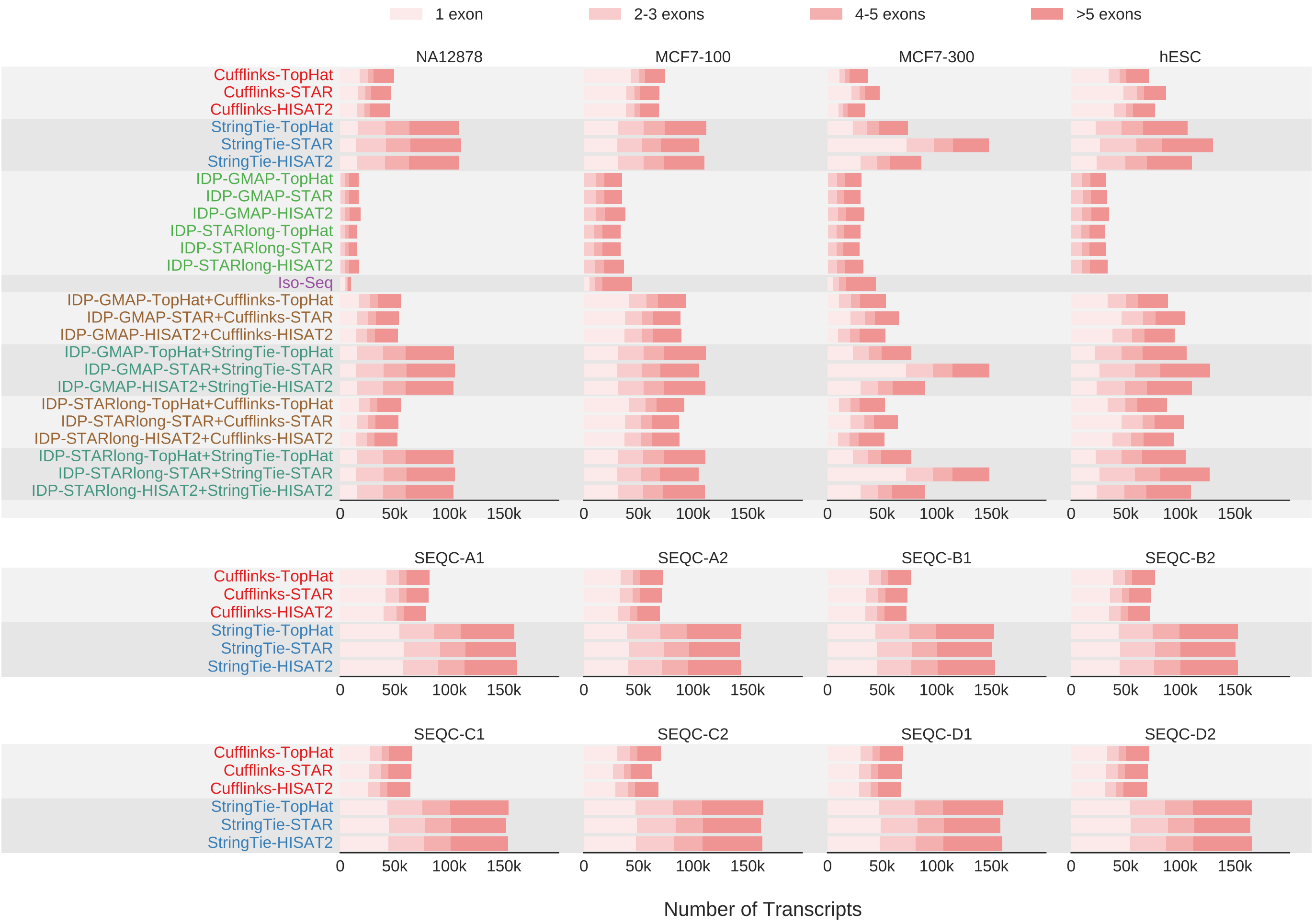

Supplementary Figure 5 | Distribution of number of exons per transcripts for different transcriptome reconstruction algorithms. Labels reflect the assembler, the long-read aligner (for IDP), and the short-read aligner used, respectively, with "-" separation. The union approaches that combined predictions from short reads and long reads (shown with a "+" in the label) slightly improved the performance of short-read isoform prediction schemes.

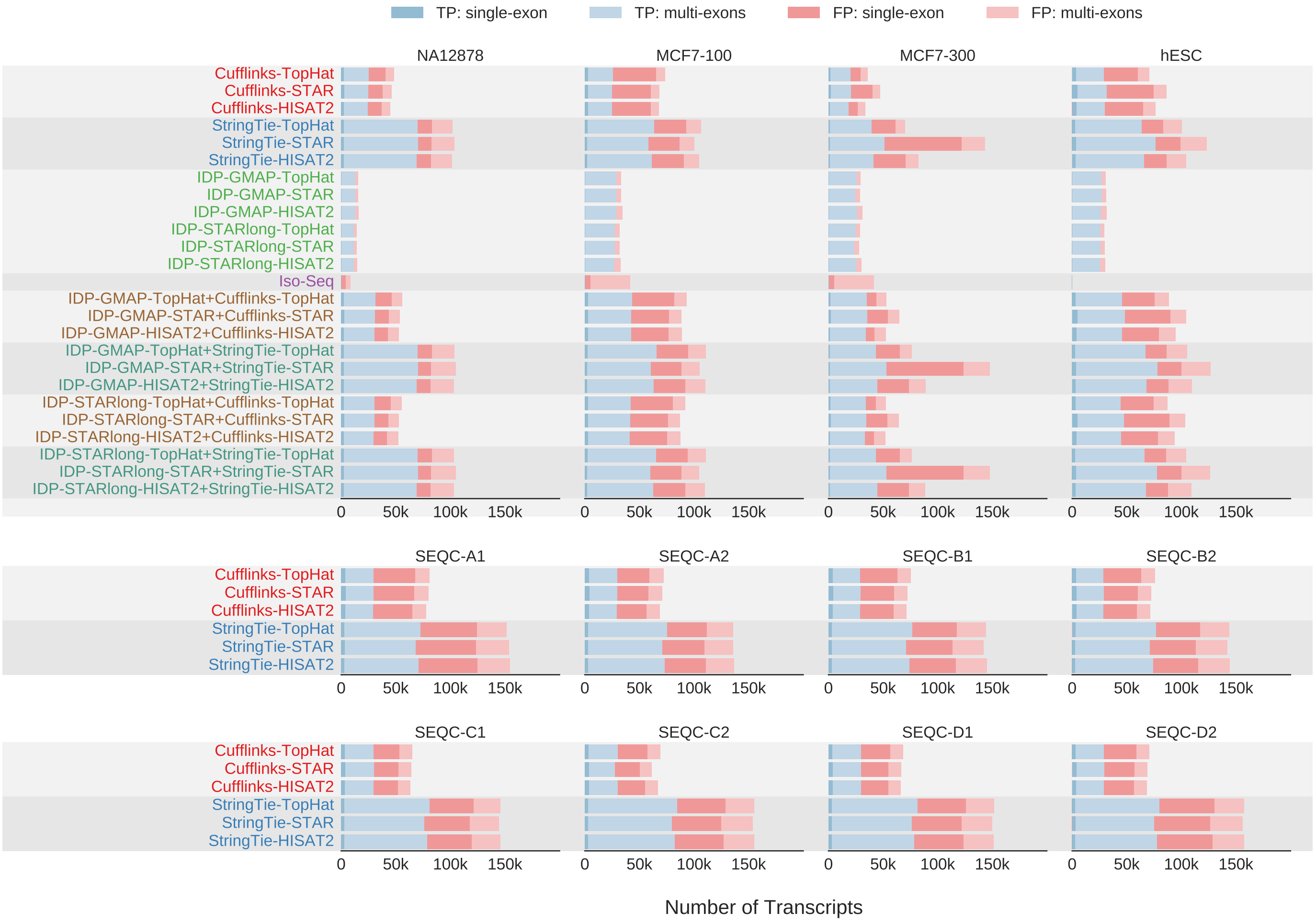

Supplementary Figure 6 | Distribution of TP and FP reported transcripts based on the number of exons for each transcriptome reconstruction algorithm. Labels reflect the assembler, the long-read aligner (for IDP), and the short-read aligner used, respectively, with "-" separation. The union approaches that combined predictions from short reads and long reads (shown with a "+" in the label) slightly improved the performance of short-read isoform prediction schemes.

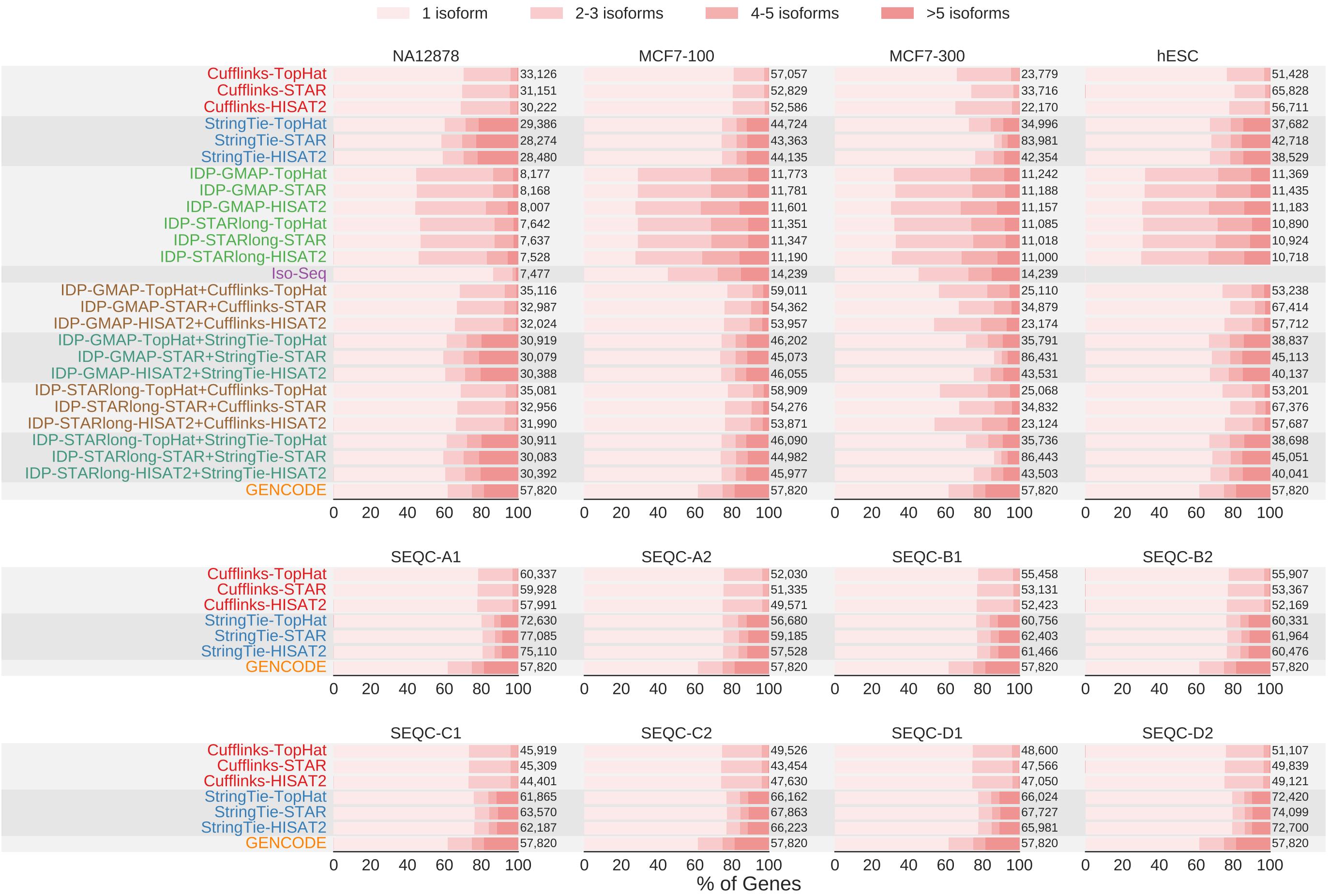

Supplementary Figure 7 | Normalized distribution of number of isoforms per gene for different transcriptome reconstruction algorithms as well as the genes in GENCODE. Labels reflect the assembler, the long-read aligner (for IDP), and the short-read aligner used, respectively, with "-" separation. The union approaches that combined predictions from short reads and long reads (shown with a "+" in the label) slightly improved the performance of short-read isoform prediction schemes.

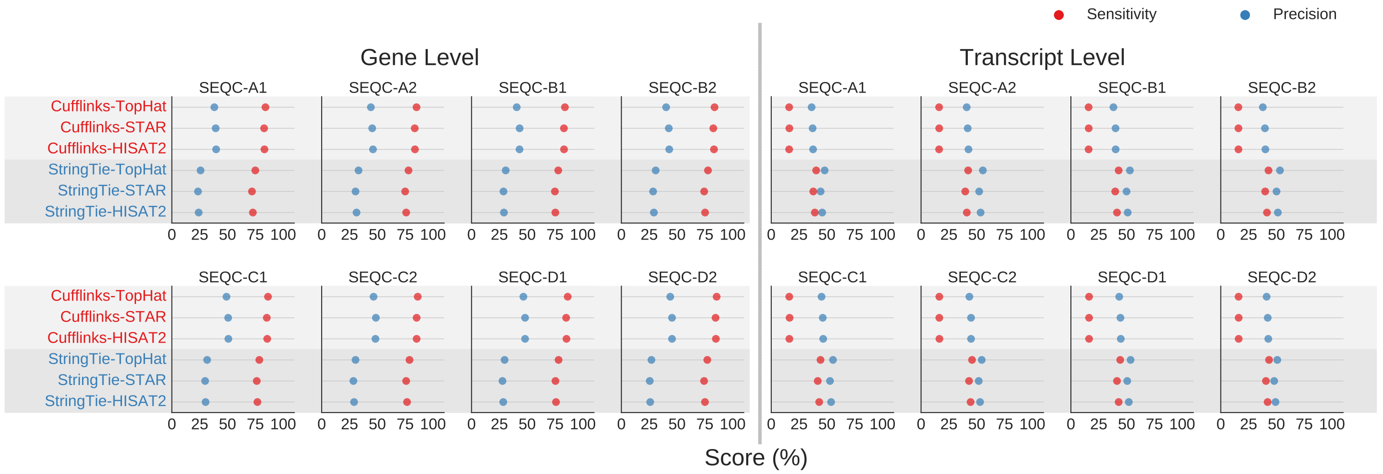

Supplementary Figure 8 | Sensitivity and precision of different transcriptome reconstruction approaches at gene and transcript level for SEQC samples. The GENCODE reference transcriptome annotation is used as the true set.

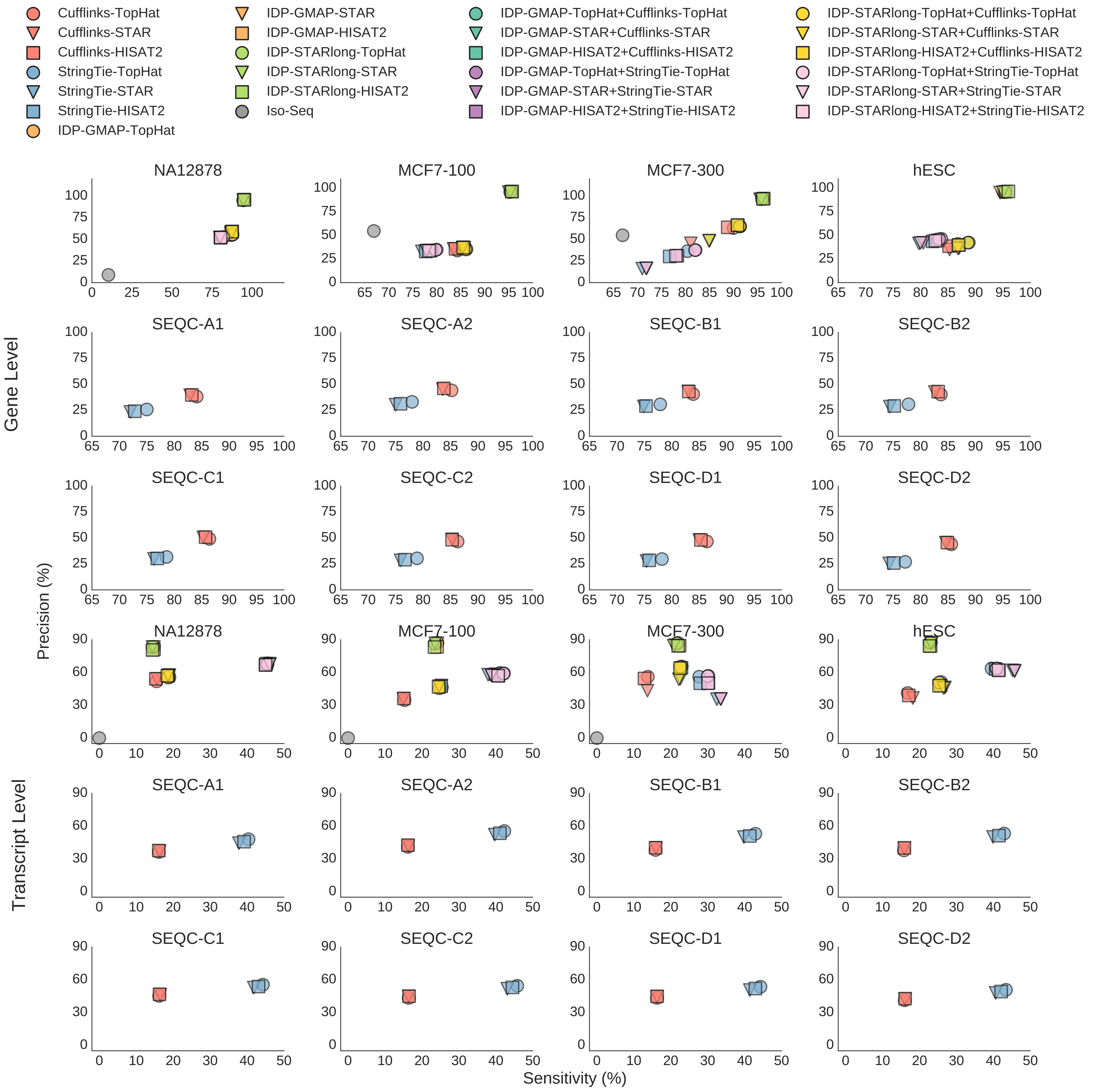

Supplementary Figure 9 | Sensitivity and precision of different transcriptome reconstruction approaches at gene and transcript level. The GENCODE reference transcriptome annotation is used as the true set.

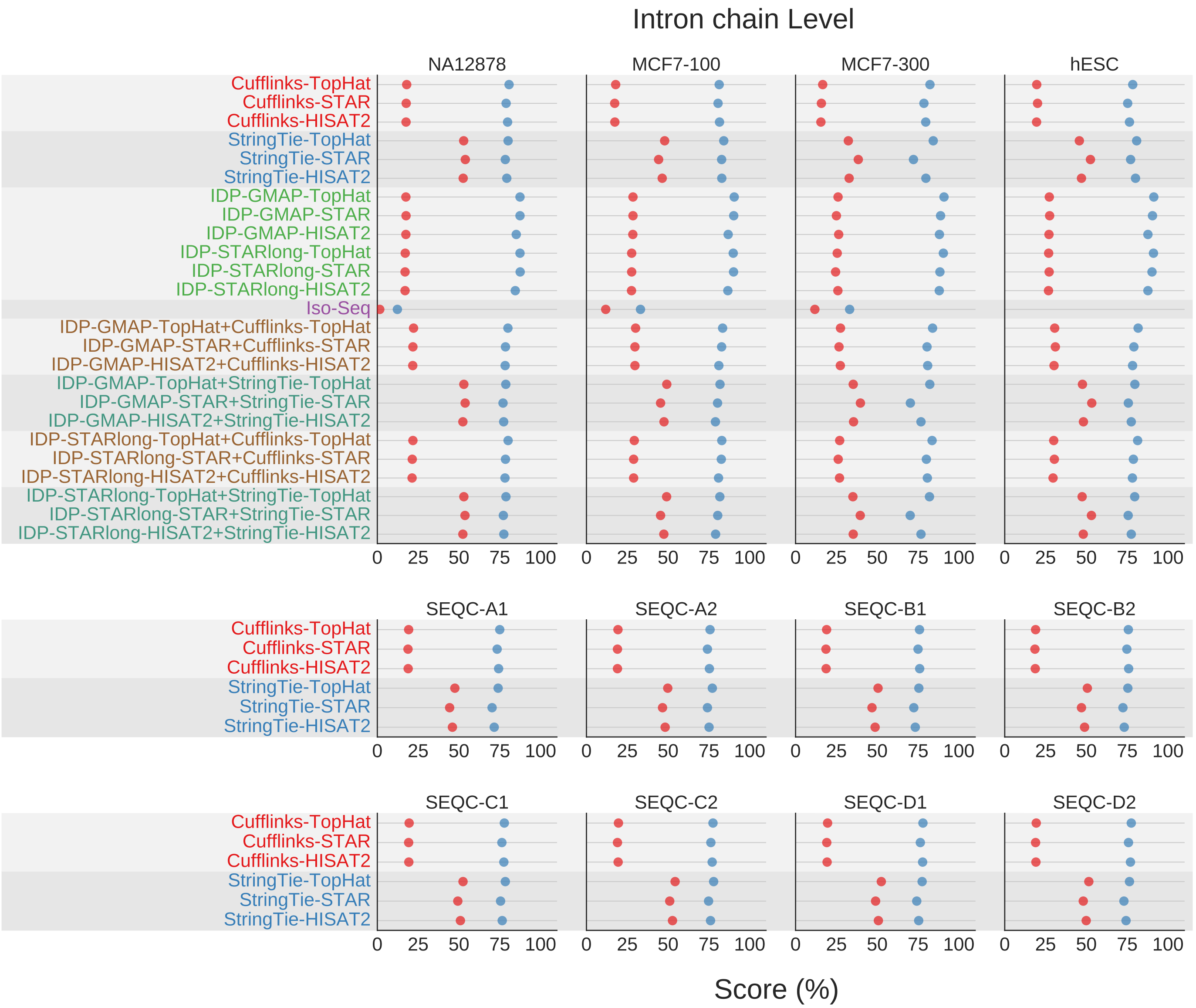

Supplementary Figure 10 | Sensitivity and precision of different transcriptome reconstruction approaches at intron-chain level. The GENCODE reference transcriptome annotation is used as the true set.

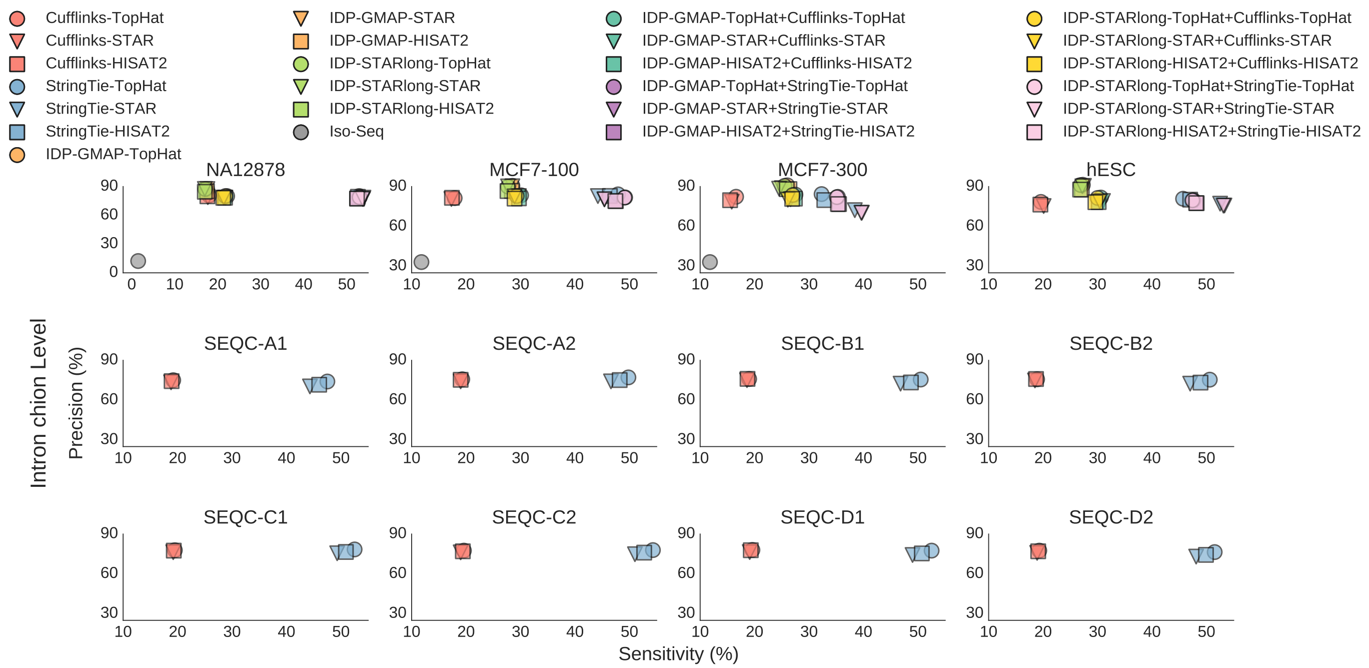

Supplementary Figure 11 | Sensitivity and precision of different transcriptome reconstruction approaches at intron-chain level. The GENCODE reference transcriptome annotation is used as the true set.

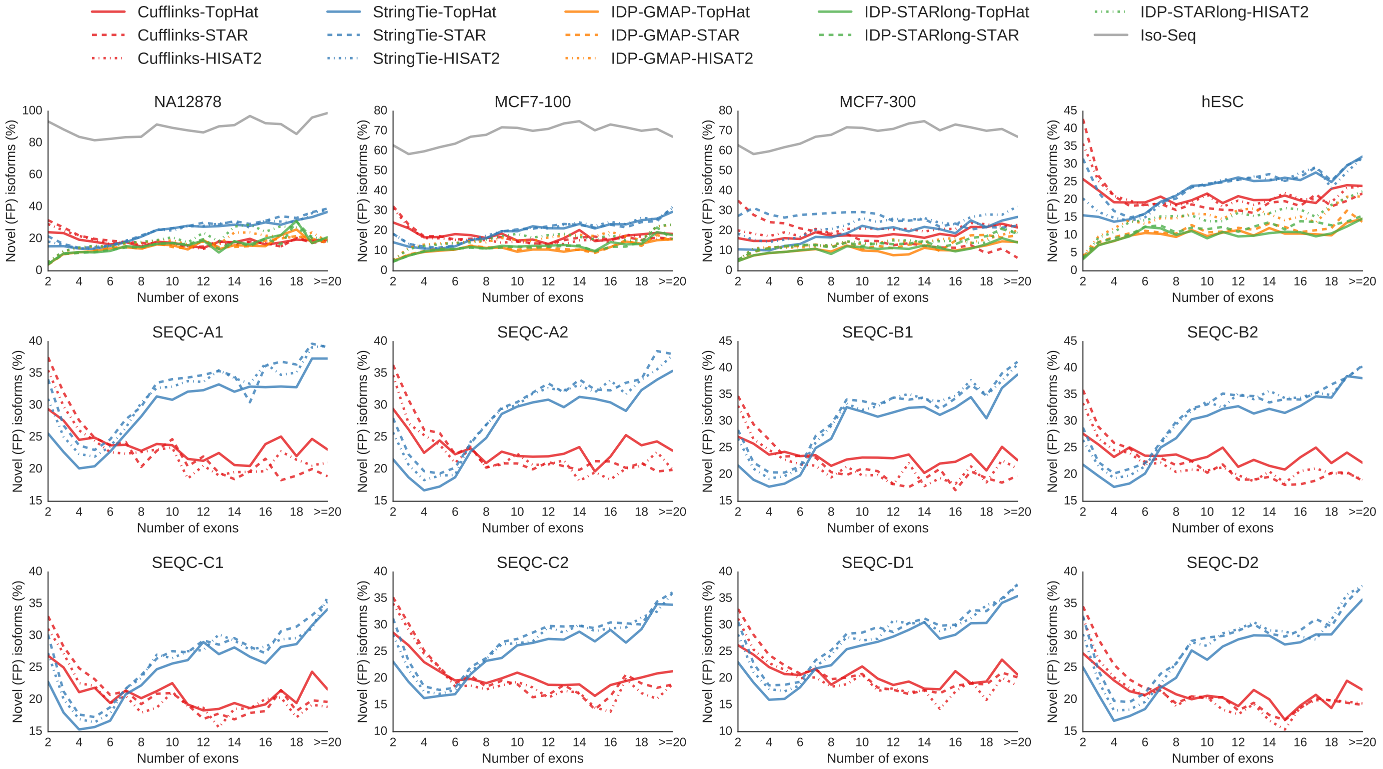

Supplementary Figure 12 | Fraction of transcripts predicted by different algorithms which are novel with respect to the GENCODE annotation versus number of introns in the transcript.

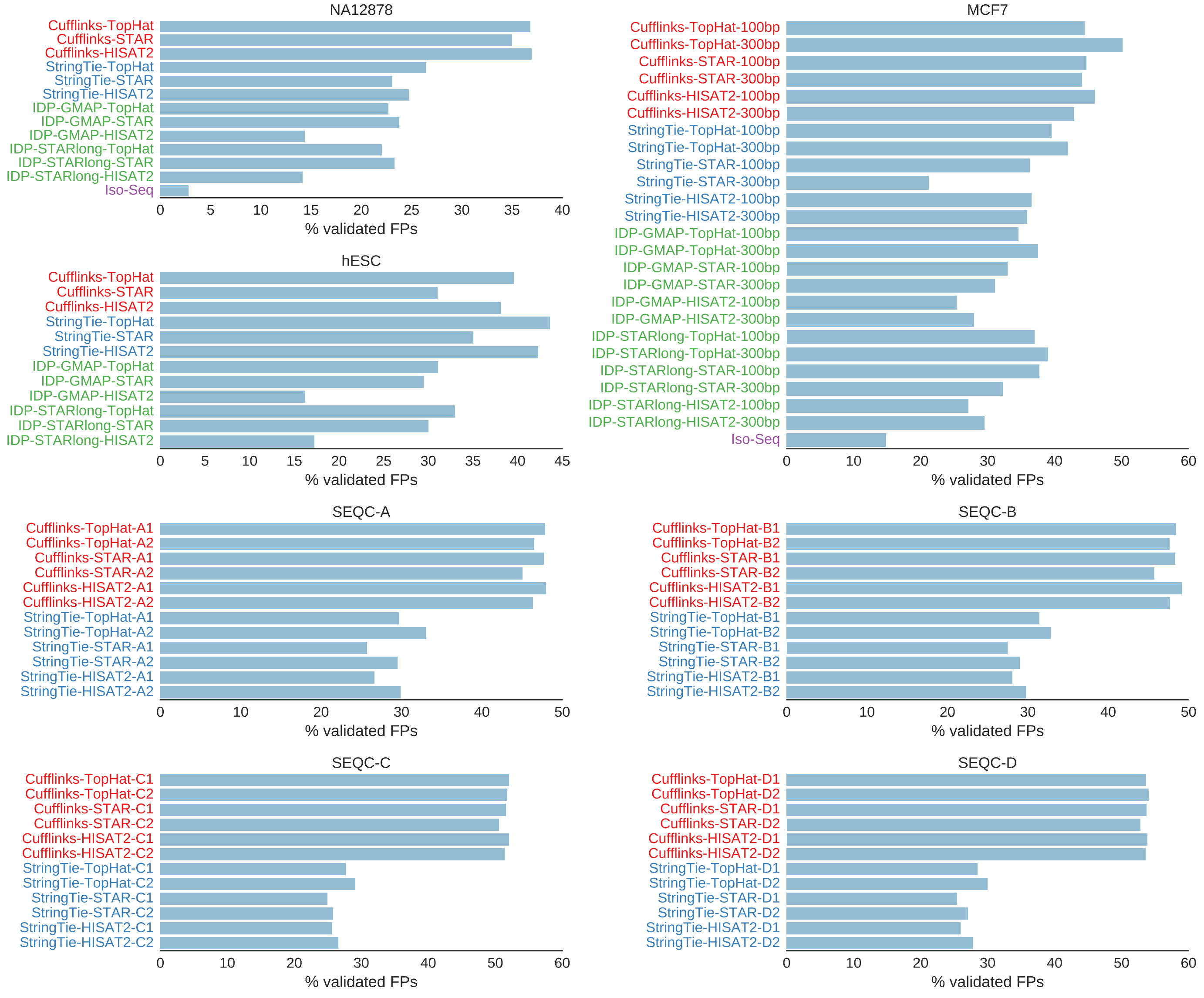

Supplementary Figure 13 | Fraction of FP transcripts predicted by different algorithms that can be validated by calls from other short- or long-read-based techniques with different assembly approaches.

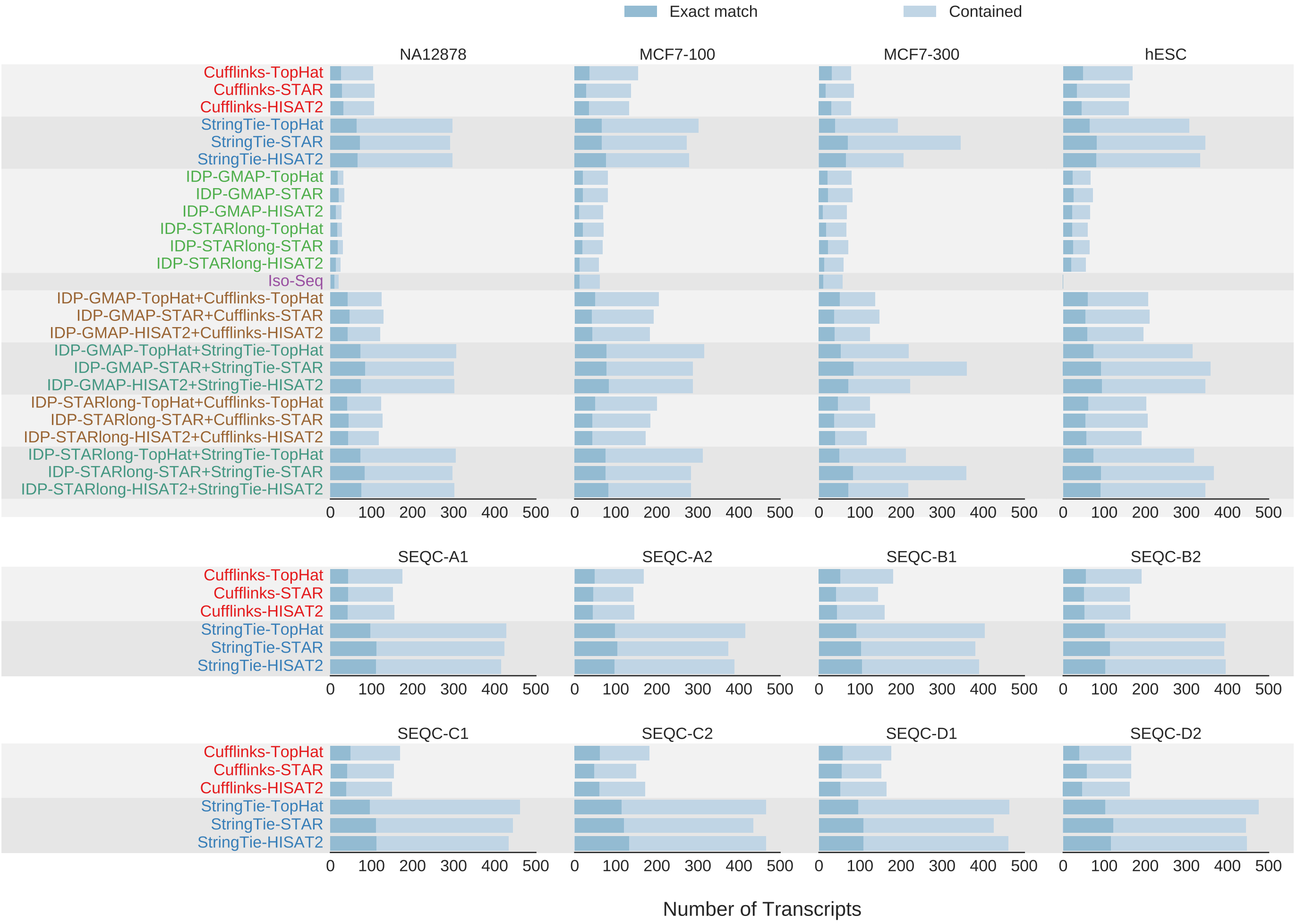

Supplementary Figure 14 | Performance of different transcriptome reconstruction algorithms in detecting novel isoforms. Novel isoforms are defined as the set of reference multi-exon transcripts in GENCODE (v19) that are missing in the reference annotation that methods were aware of during the isoform detection (Ensembl). Labels reflect the assembler, the long-read aligner (for IDP), and the short-read aligner used, respectively, with “-” separation.

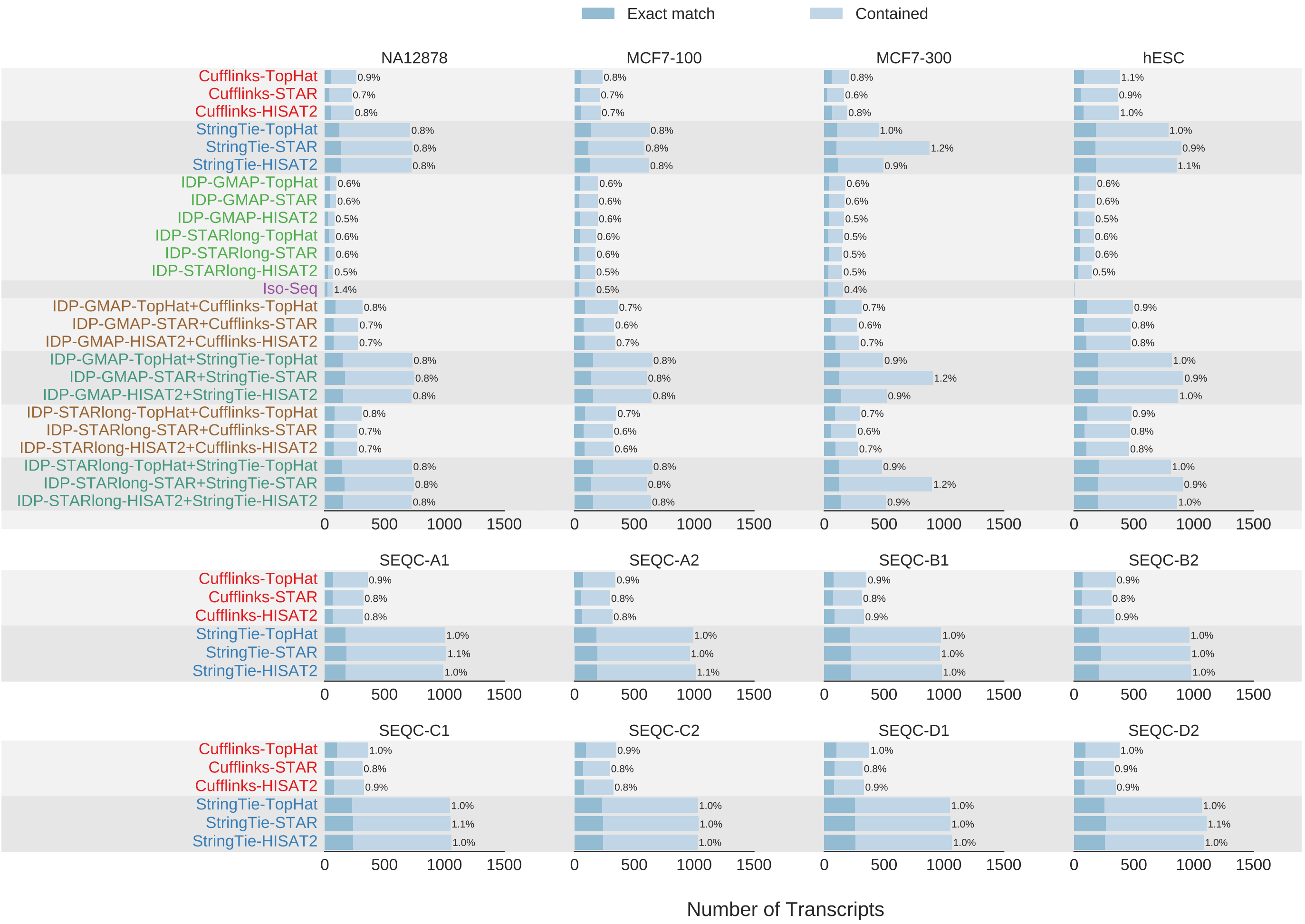

Supplementary Figure 15 | Performance of different transcriptome reconstruction algorithms in detecting multi-exon transcripts in GENCODE (v25) which are missing in GENCODE (v19), and thus was assumed as FP in the predictions. Labels reflect the assembler, the long-read aligner (for IDP), and the short-read aligner used, respectively, with "-" separation. Numbers reflect the percentage of such novel predictions among all predicted multi-exon transcripts

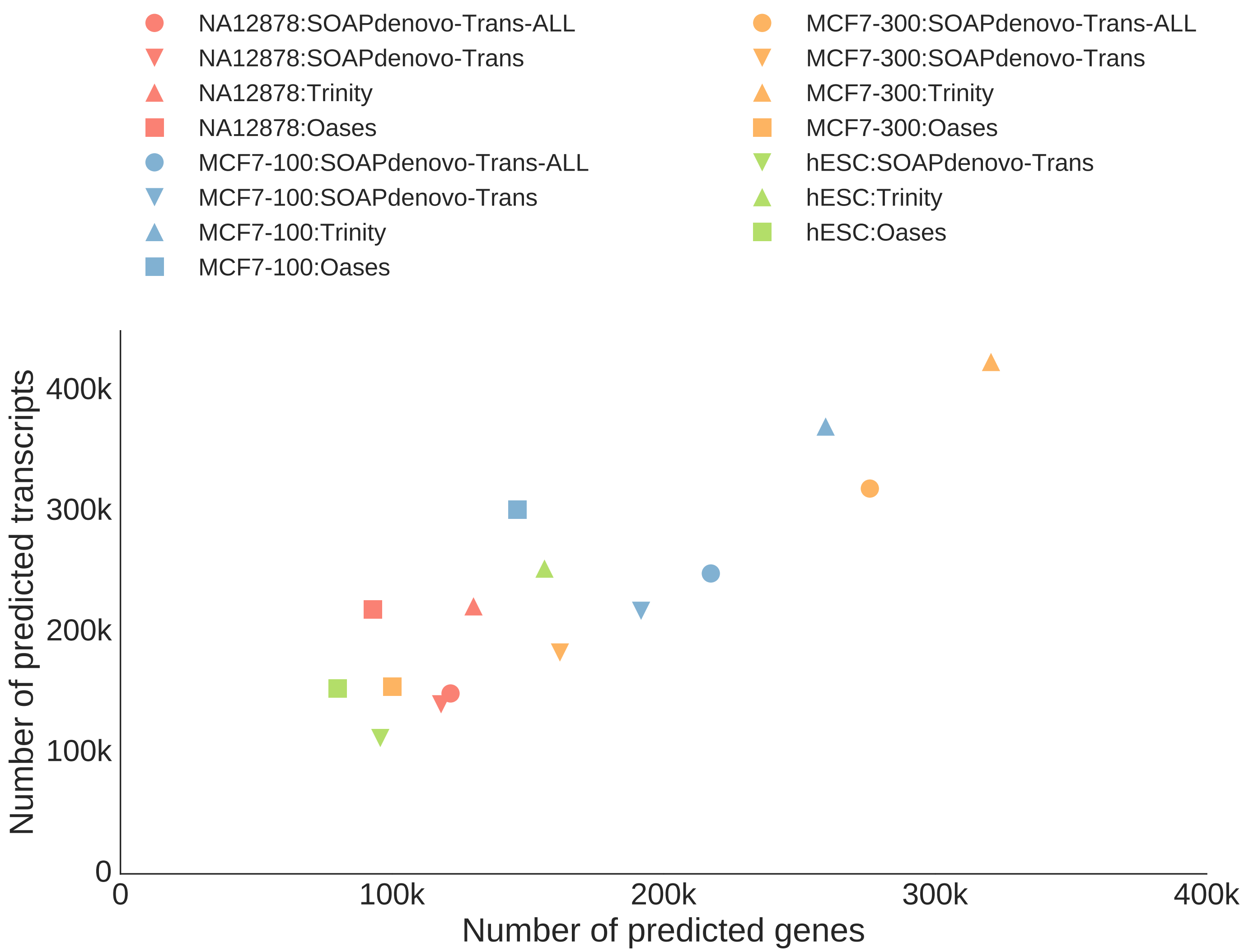

Supplementary Figure 16 | Number of genes and transcripts predicted by different *de novo* transcriptome assembly techniques. Analysis is restricted to isoforms of length > 300bp.

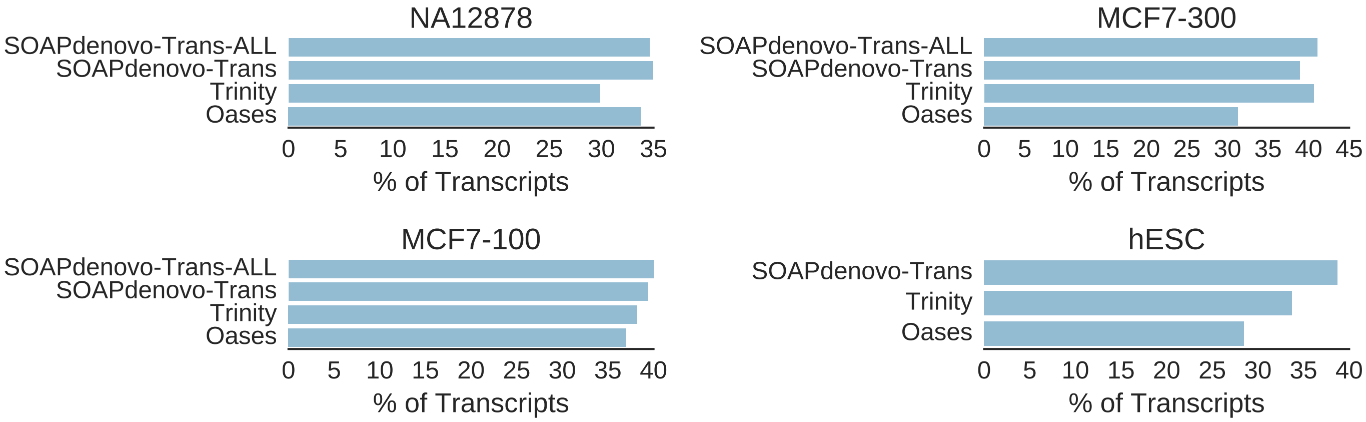

Supplementary Figure 17 | Percentage of the assembled transcripts that are fully contained in a reference transcript which covers multiple assembled transcripts. These assembled transcripts are potentially false calls formed by splitting the transcripts because of missing coverage.

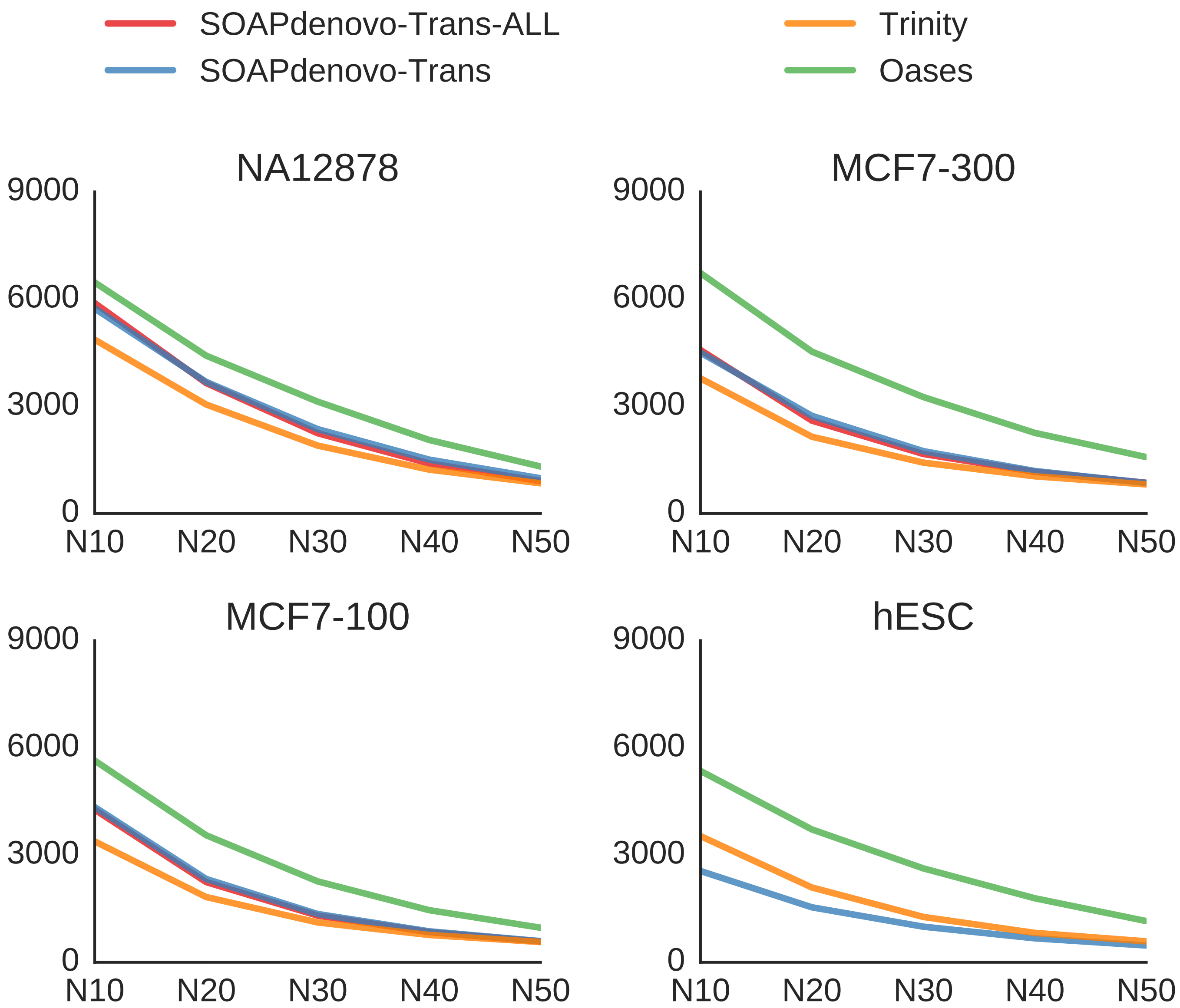

Supplementary Figure 18 | N10-N50 values for different *de novo* transcriptome assembly techniques measured for single longest isoform per gene. (Nx is the contig length for which at least x% of the assembled transcript nucleotides were found in contigs that were at least of Nx length.)

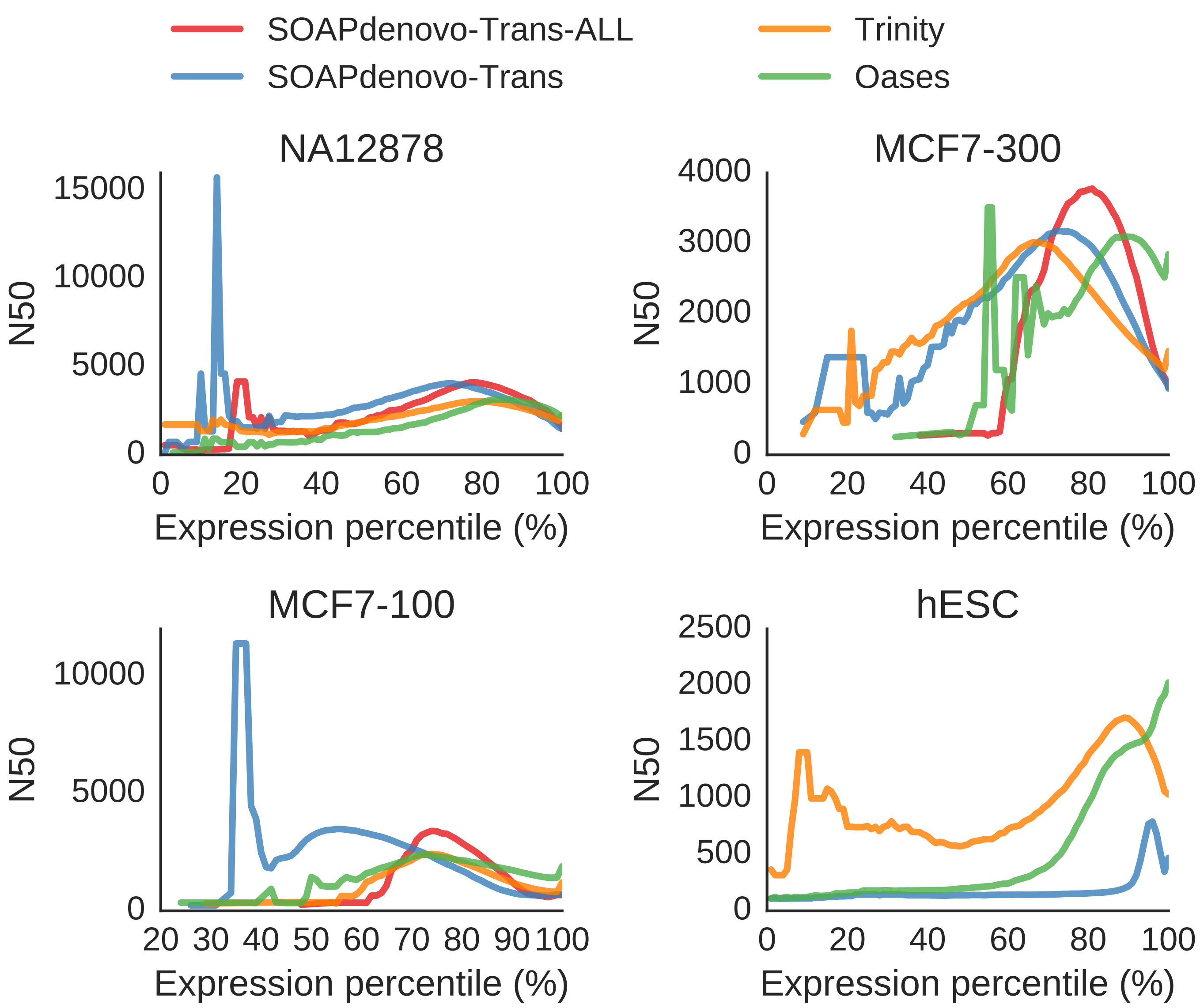

Supplementary Figure 19 | ExN50 value at different expression percentiles. For ExN50, the N50 statistic is computed at percentile x, for only the top most highly expressed transcripts that represent x% of the total normalized expression data. Expression values are measured using kallisto.

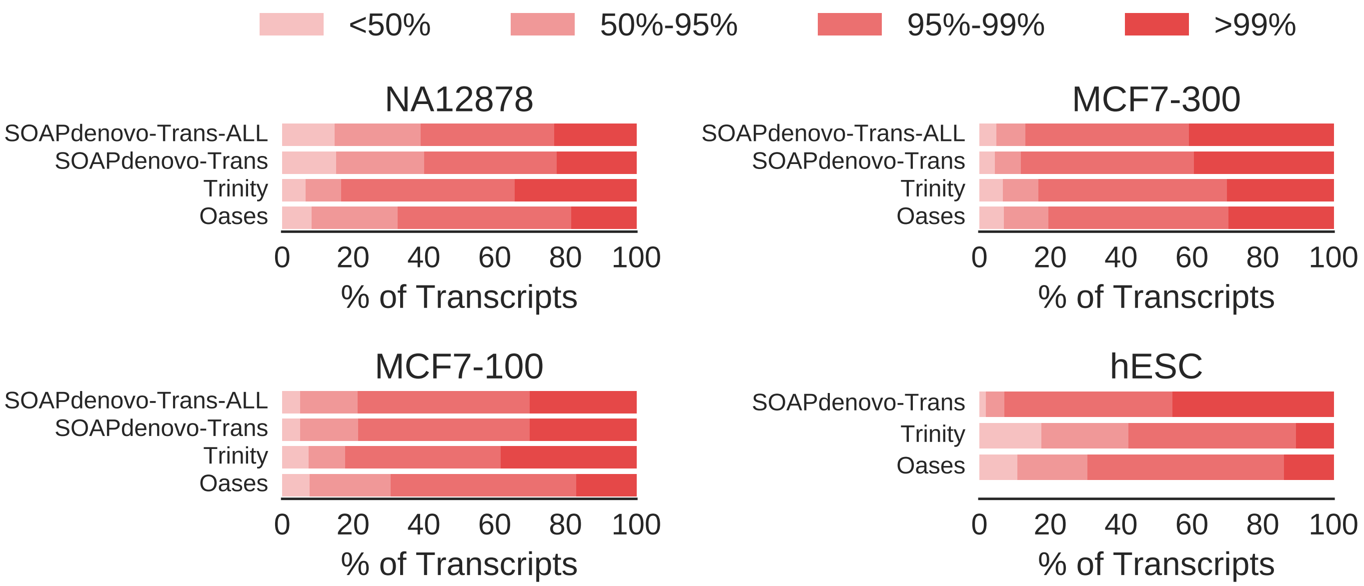

Supplementary Figure 20 | Alignment identity of the predicted isoforms (of length > 300bp) for different *de novo* transcriptome assemblers when aligned to the reference genome using GMAP.

a

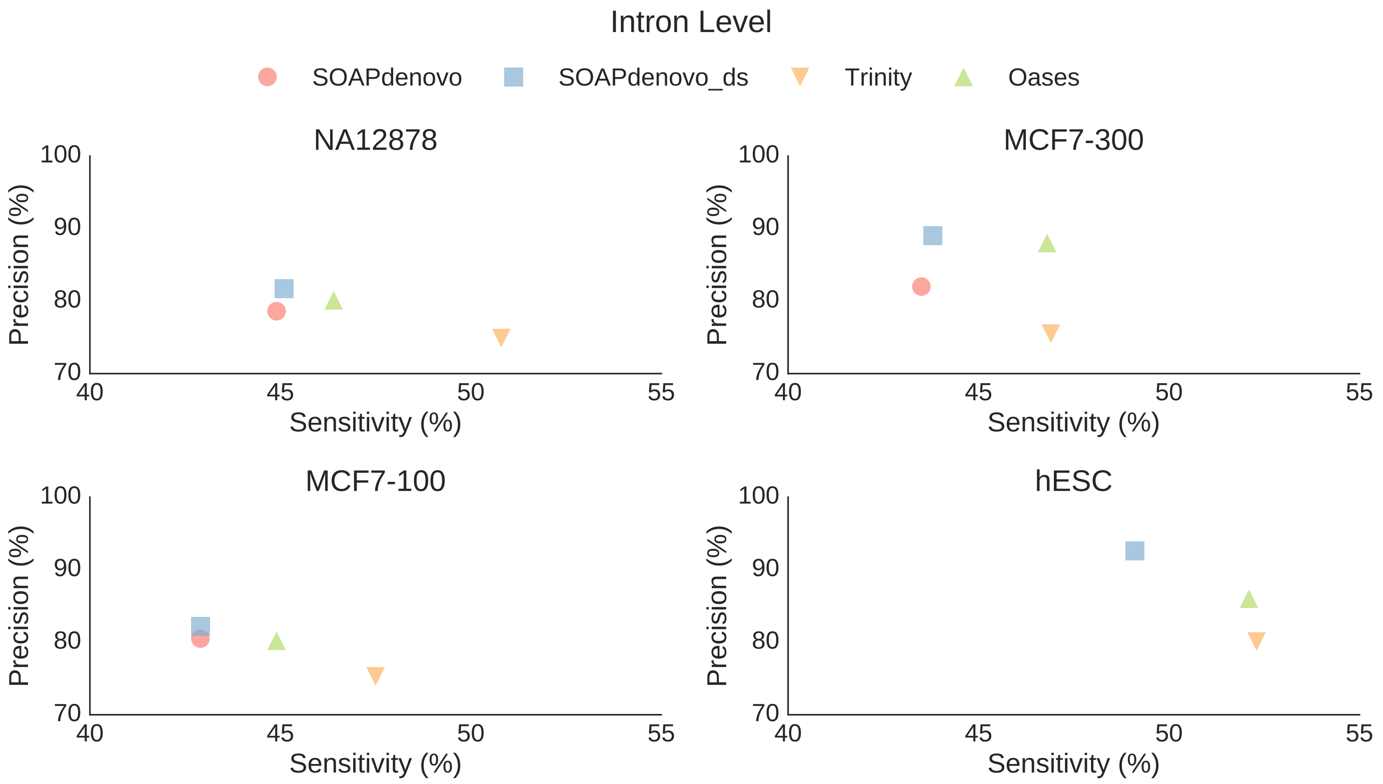

b

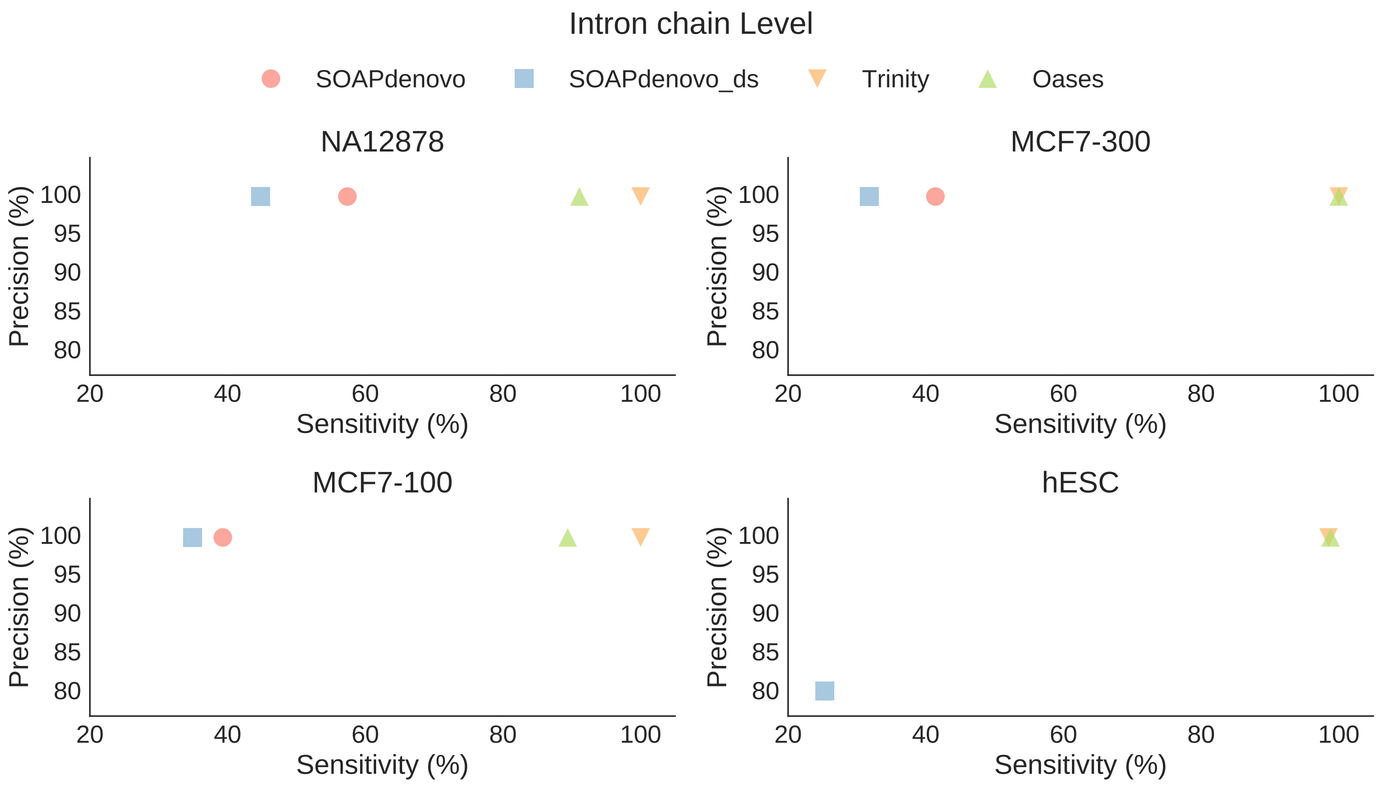

Supplementary Figure 21 | Sensitivity and precision of different *de novo* transcriptome assemblers at (a) intron and (b) intron-chain levels. The GENCODE reference transcriptome annotation is used as the true set.

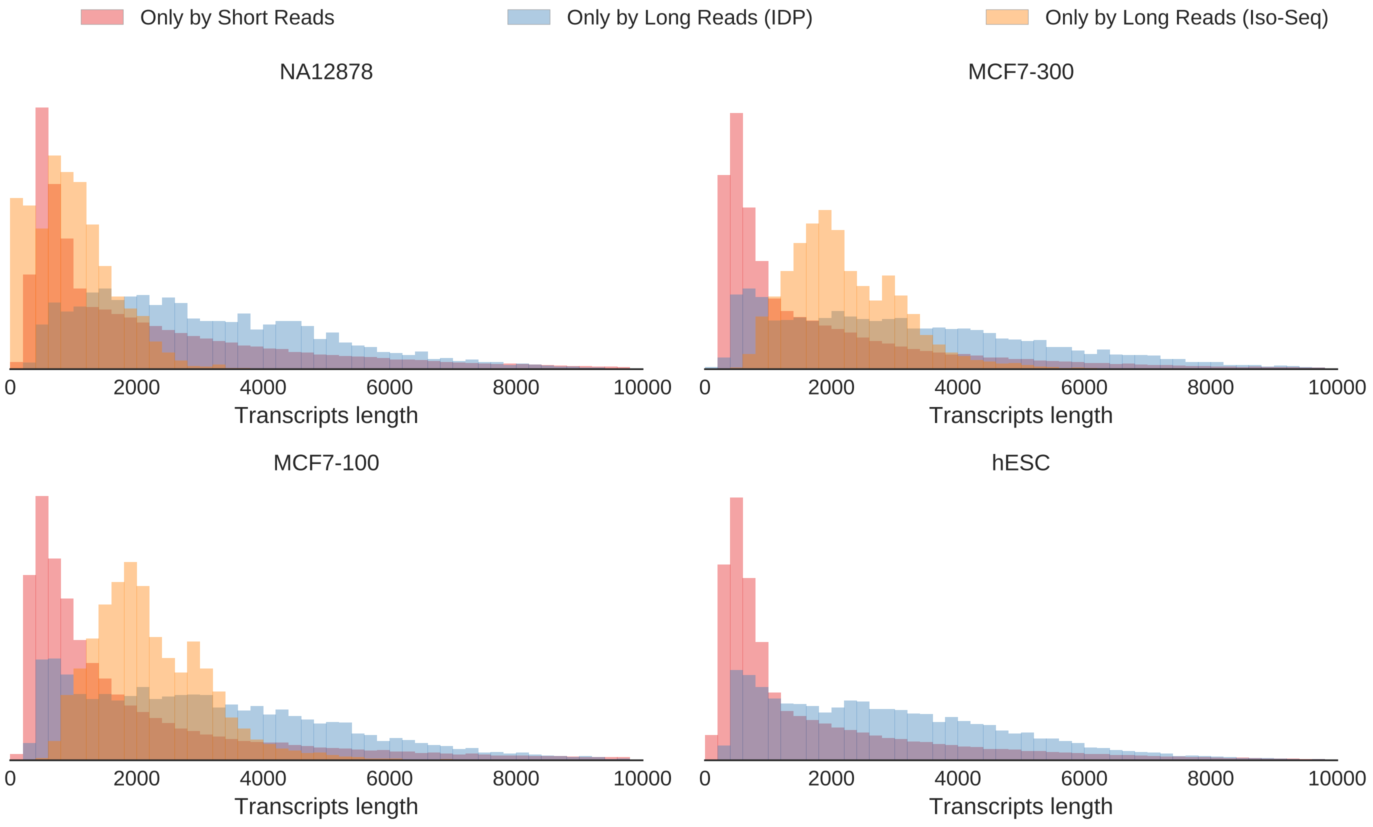

Supplementary Figure 22 | Distribution of transcripts length identified only by short-read-based techniques or identified only by long-read-based techniques. Ranksum test on private calls by long- or short-read techniques reveals significantly different distributions (p-val=0).

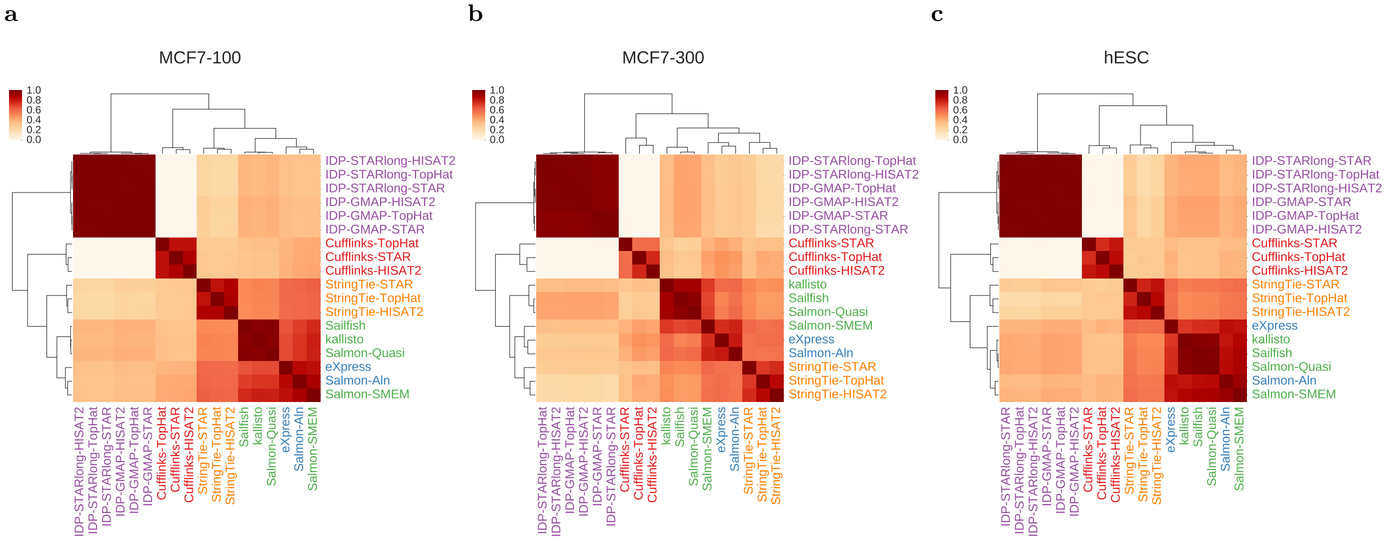

Supplementary Figure 23 | Clustering of different transcript abundance estimation schemes based on the Spearman rank correlation of their log expressions on (a) MCF7-100, (b) MCF7-300, (c) hESC.

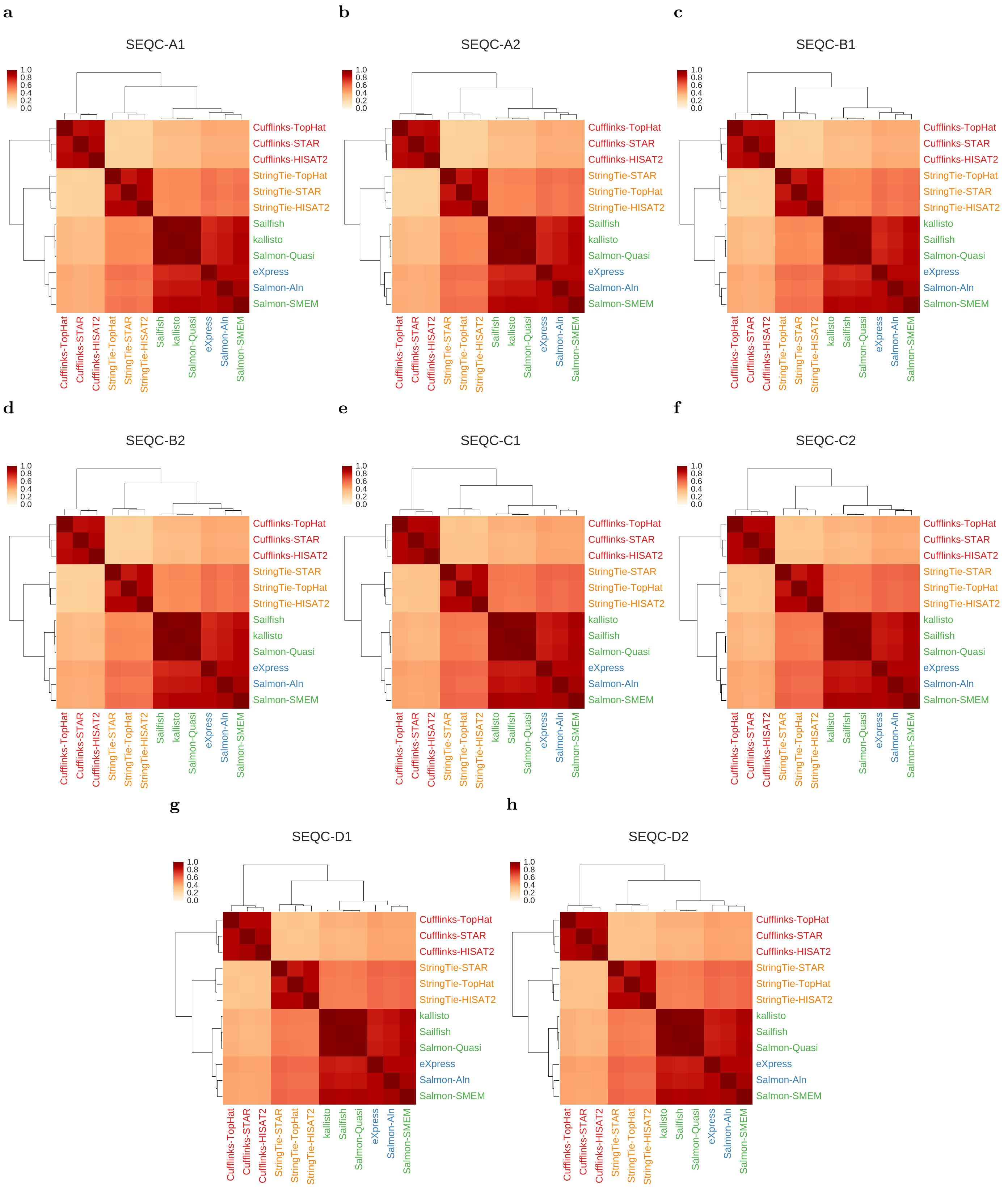

Supplementary Figure 24 | Clustering of different transcript abundance estimation schemes based on the Spearman rank correlation of their log expressions on (a) SEQC-A1, (b) SEQC-A2, (c) SEQC-B1, (d) SEQC-B2, (e) SEQC-C1, (f) SEQC-C2, (g) SEQC-D1, (h) SEQC-D2.

a

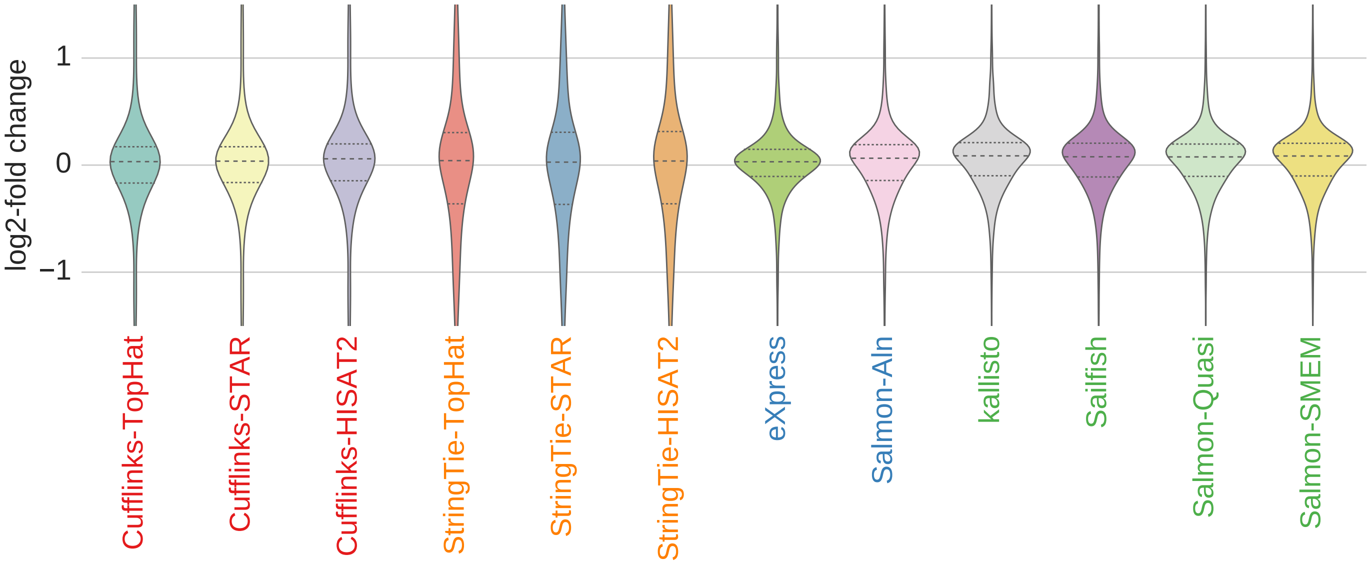

b

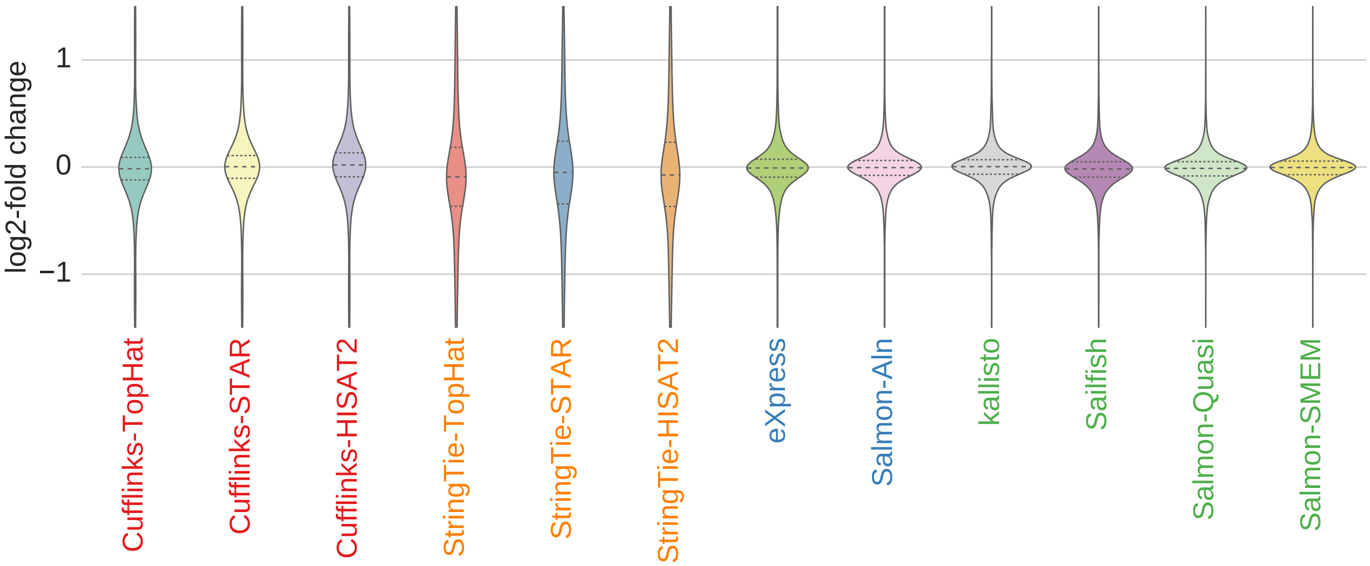

Supplementary Figure 25 | Distribution of log2-fold change of expressions between samples in (a) SEQC-A1 and SEQC-A2, (b) SEQC-B1 and SEQC-B2 samples for different transcript abundance estimation algorithms. For each method dashed line represents the mean of the distribution and the dotted lines represents the quartiles.

a

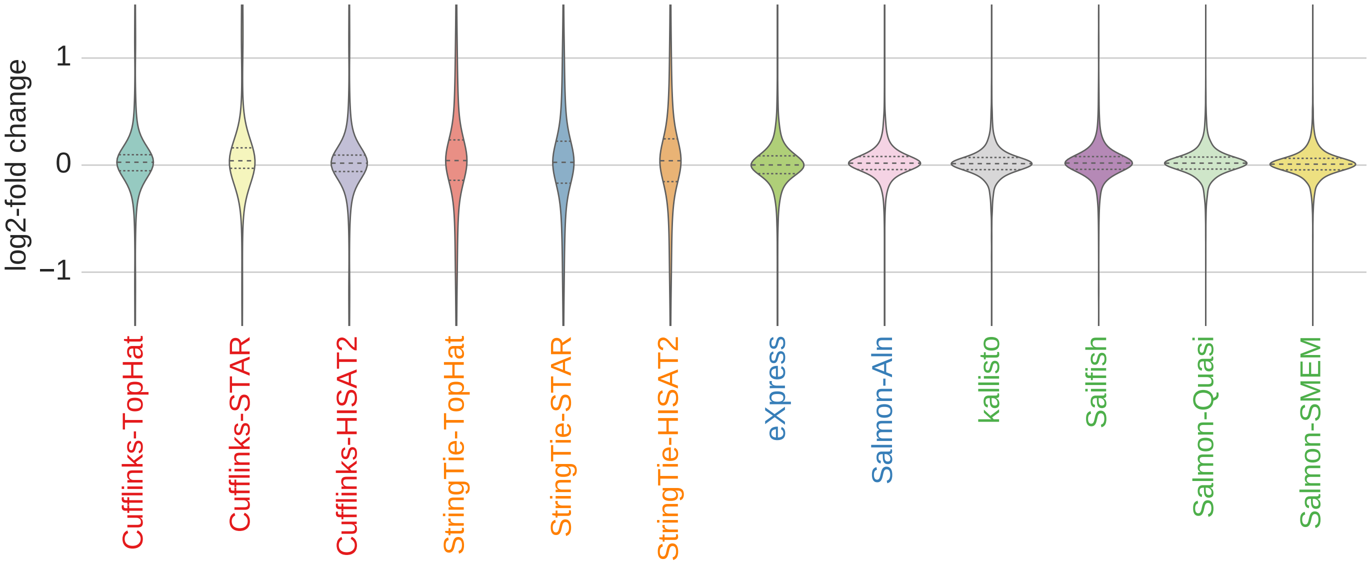

b

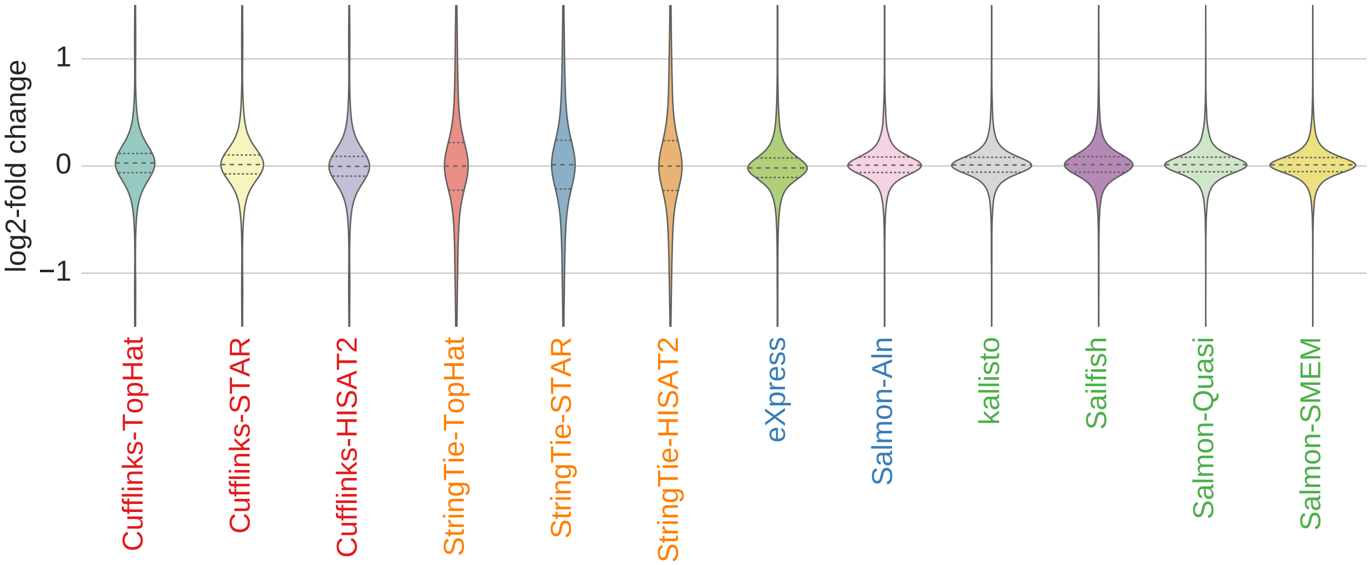

Supplementary Figure 26 | Distribution of log2-fold change of expressions between samples in (a) SEQC-C1 and SEQC-C2, (b) SEQC-D1 and SEQC-D2 samples for different transcript abundance estimation algorithms. For each method dashed line represents the mean of the distribution and the dotted lines represents the quartiles.

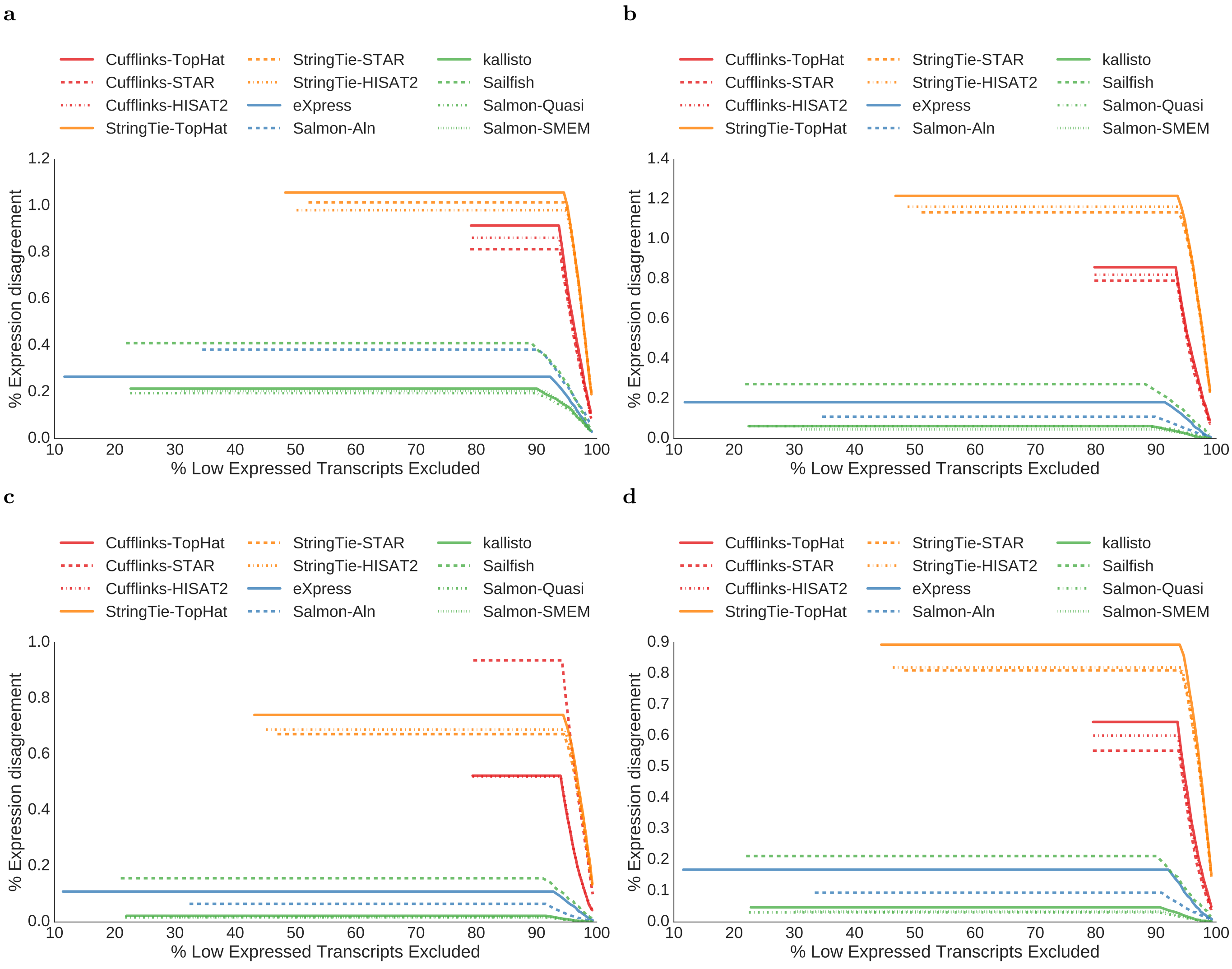

Supplementary Figure 27 | Percentage of expression disagreement between (a) SEQC-A1 and SEQC-A2, (b) SEQC-B1 and SEQC-B2, (c) SEQC-C1 and SEQC-C2, (d) SEQC-D1 and SEQC-D2 samples when low expressed transcripts are discarded with different thresholds.

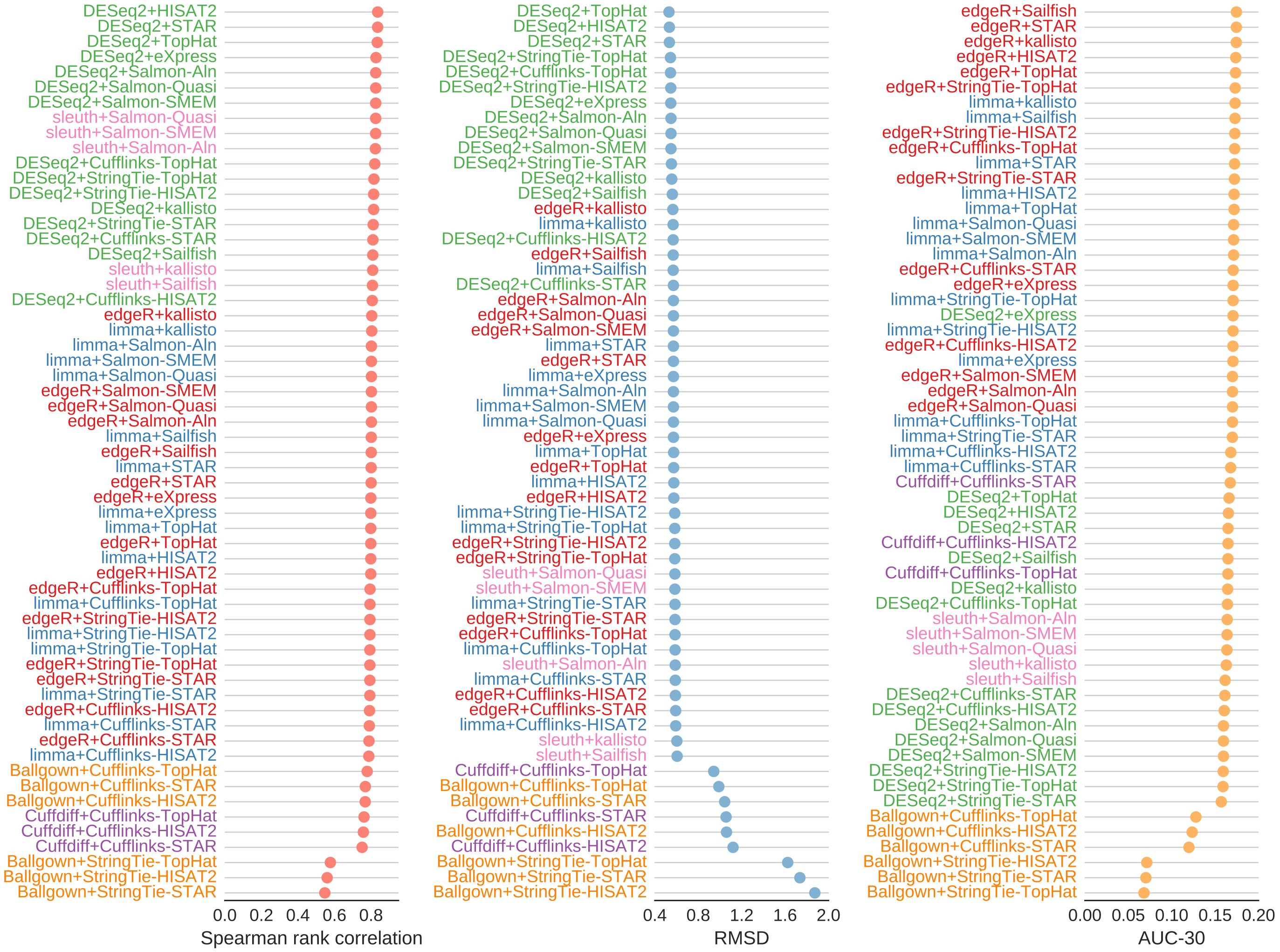

Supplementary Figure 28 | Spearman rank correlation, root-mean-score-deviation (RMSD), and AUC-30 scores for differential analysis of qPCR measured genes on SEQC-C vs. SEQC-D samples. Spearman rank correlation and RMSD scores are measured between the log2-fold change of the qRT-PCR and RNA-seq tools. AUC-30 score represents the area under the ROC curve up to the false positive rate of 30%.

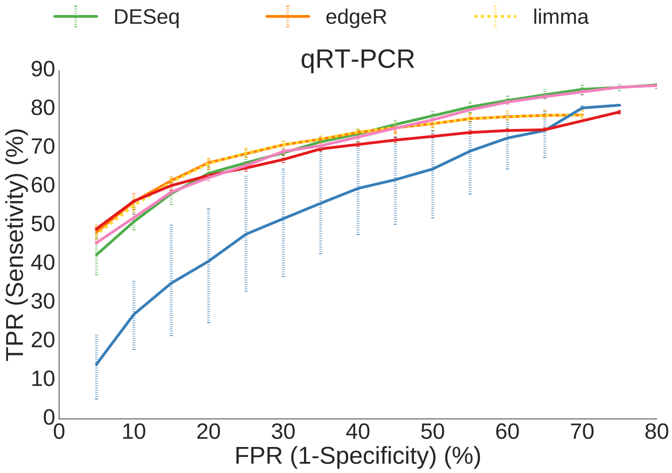

Supplementary Figure 29 | ROC analysis of qRT-PCR measured genes (left) and ERCC (right) genes for SEQC-C vs. SEQC-D differential analysis. For each differential analysis tool the plot reflects average performance when different alignment-based and alignment-free tools are used for abundance estimation and error bar shows the maximum an minimum variations. Results for each tool combination are shown in Supplementary Figs 31 and 36

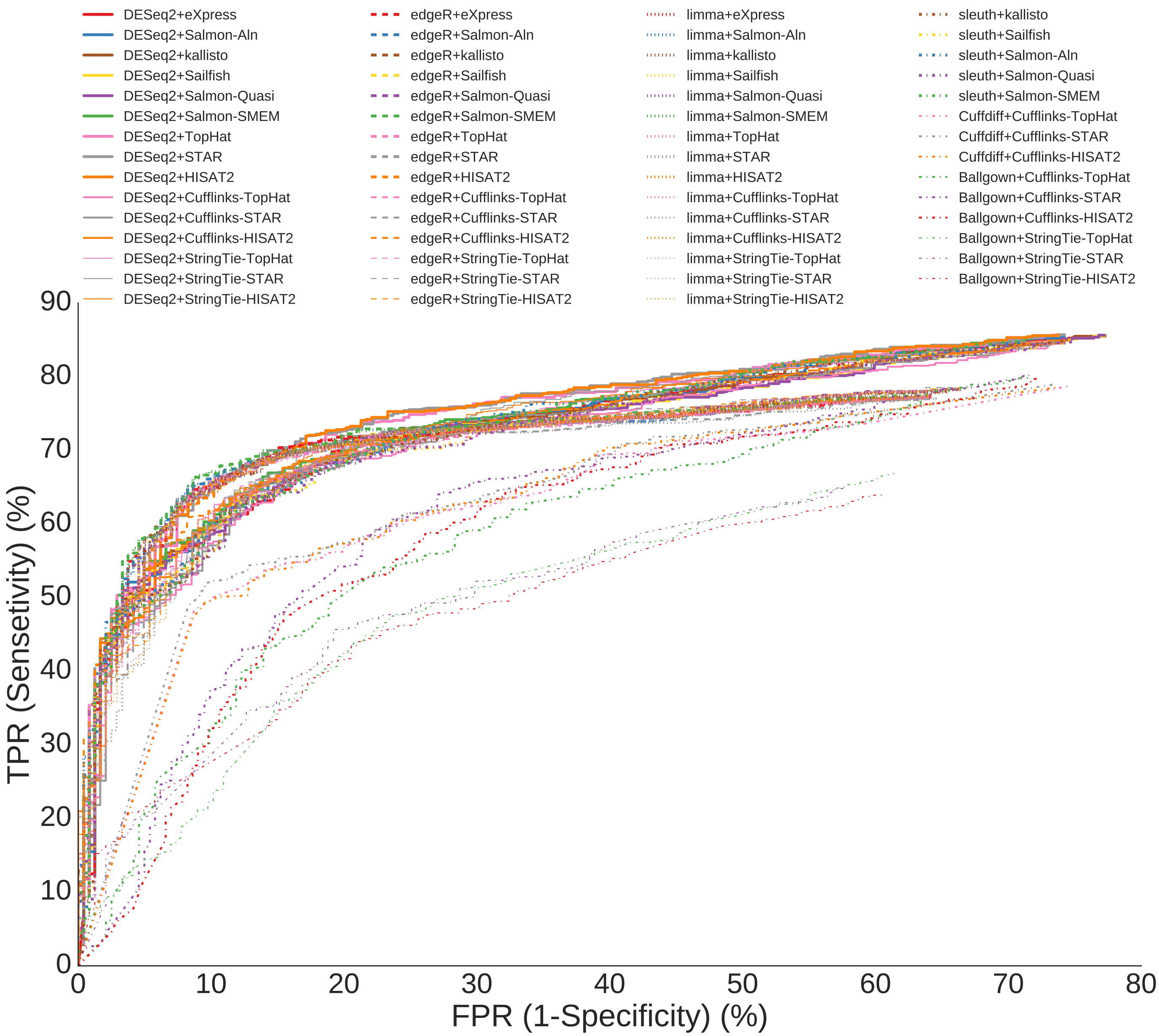

Supplementary Figure 30 | ROC analysis of qRT-PCR measured genes for SEQC-A vs. SEQC-B differential analysis.

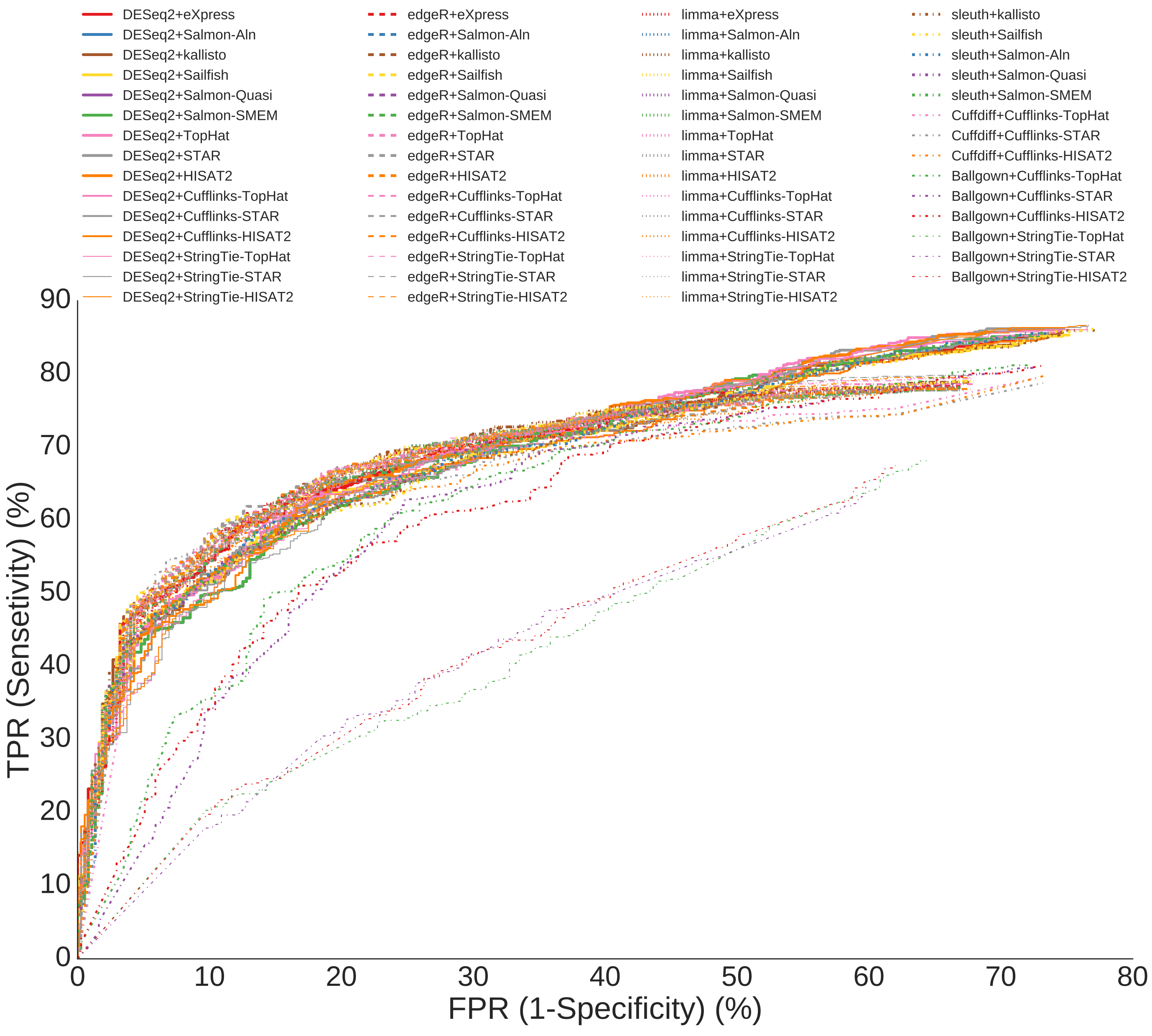

Supplementary Figure 31 | ROC analysis of qRT-PCR measured genes for SEQC-C vs. SEQC-D differential analysis.

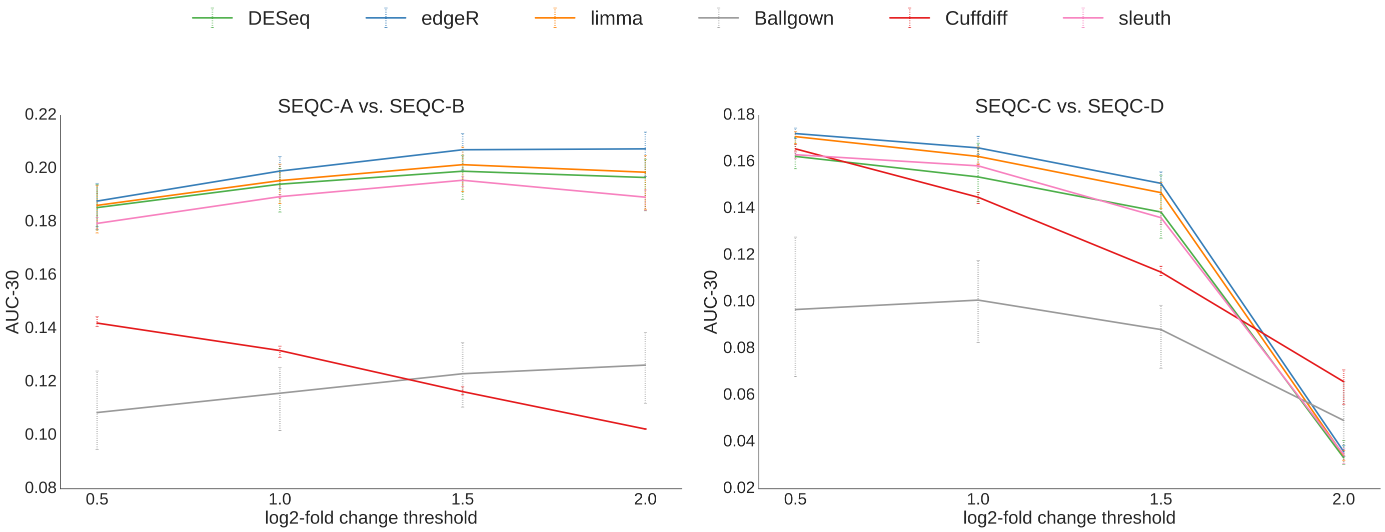

Supplementary Figure 32 | AUC-30 score at increasing cut off threshold for log2-fold change in the qRT-PCR experiment. Higher threshold values result in more confidence in the (fewer) set of differentially expressed genes in PCR experiment. For each differential analysis tool the plot reflects average performance when different alignment-based and alignment-free tools are used for abundance estimation and error bar shows the maximum an minimum variations. Results for each tool combination are shown in Supplementary Figs 33 and 34

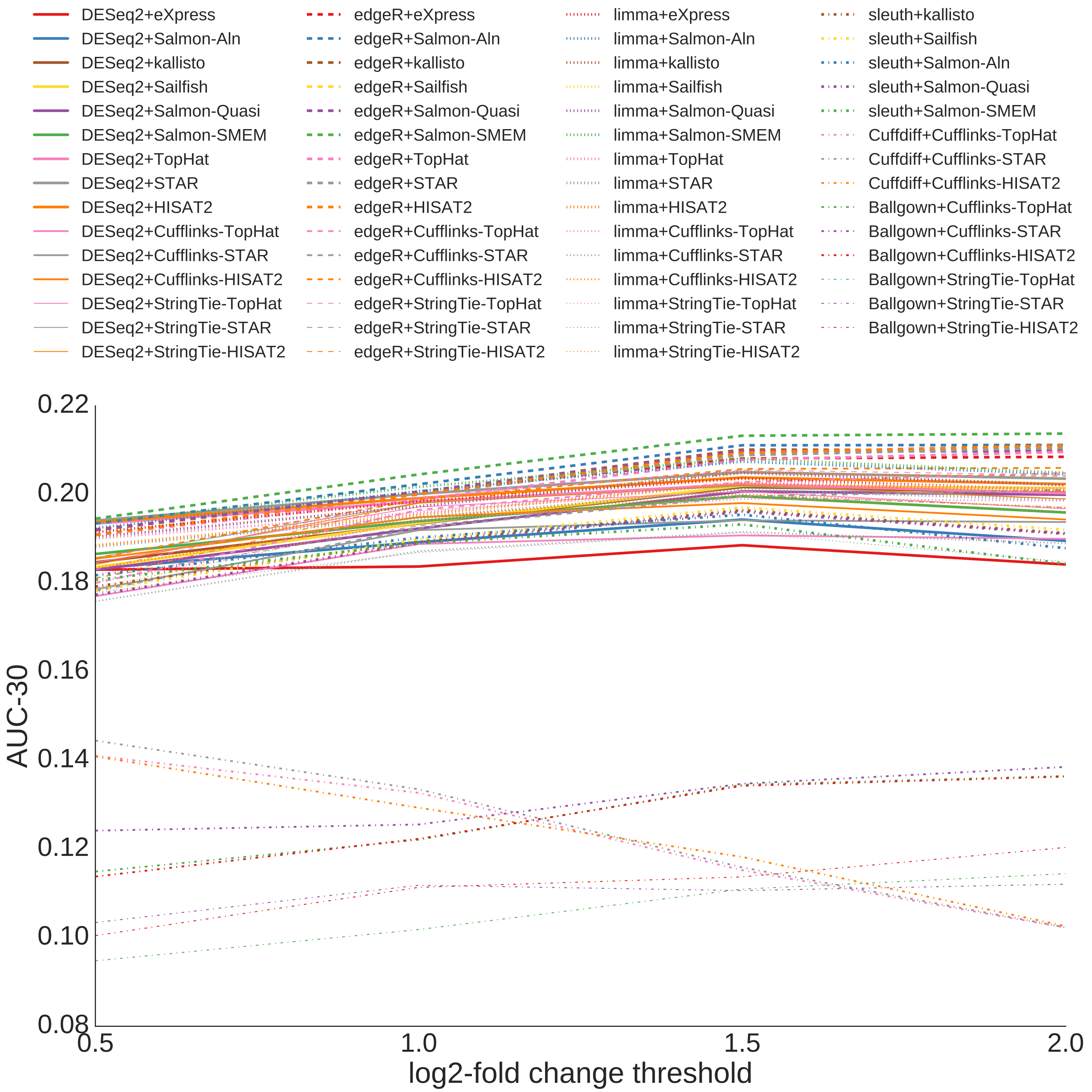

Supplementary Figure 33 | AUC-30 score at increasing cut off threshold for log2-fold change in the qRT-PCR experiment for SEQC-A vs. SEQC-B differential analysis. Higher threshold values result in more confidence in the (fewer) set of differentially expressed genes in PCR experiment.

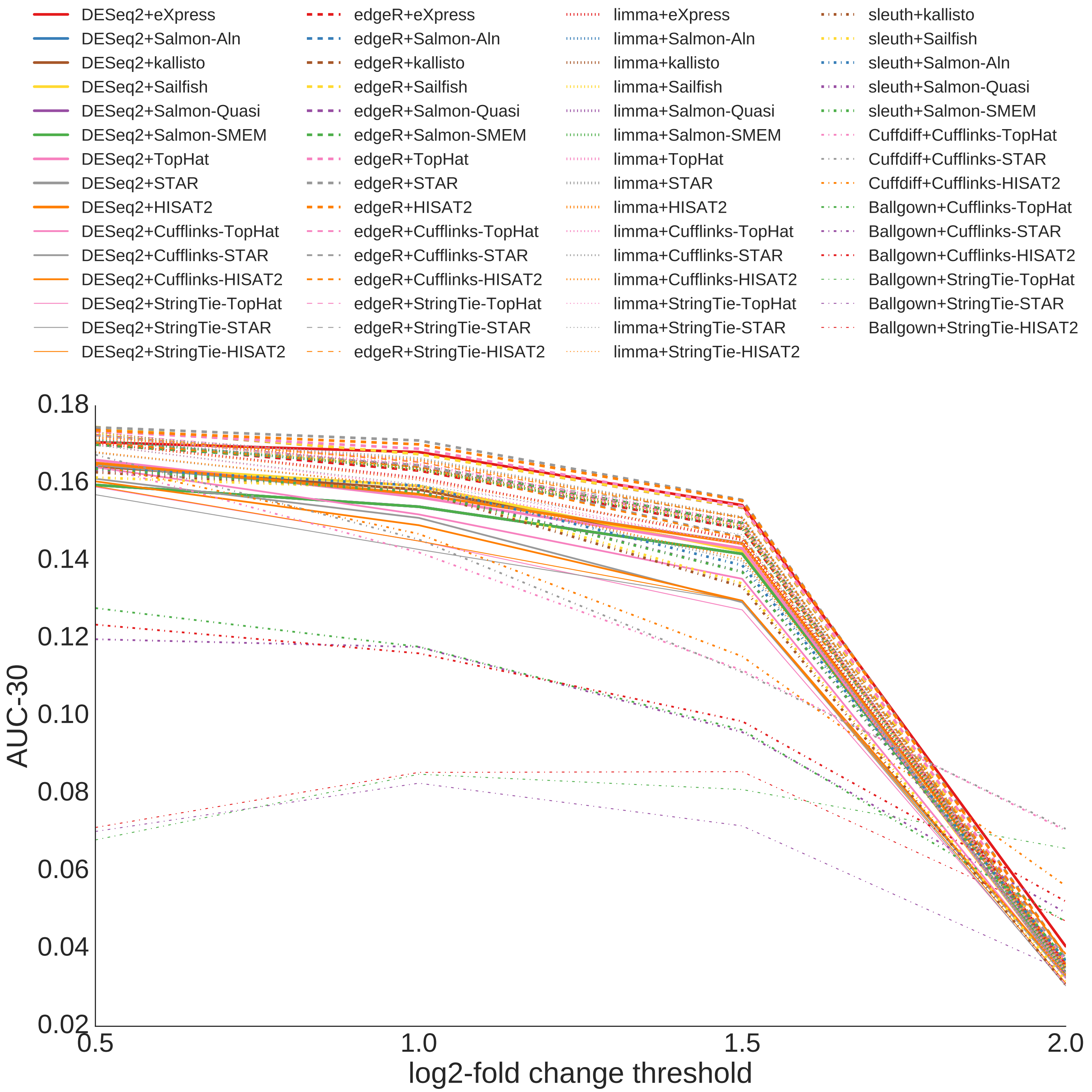

Supplementary Figure 34 | AUC-30 score at increasing cut off threshold for log2-fold change in the qRT-PCR experiment for SEQC-C vs. SEQC-D differential analysis. Higher threshold values result in more confidence in the (fewer) set of differentially expressed genes in PCR experiment.

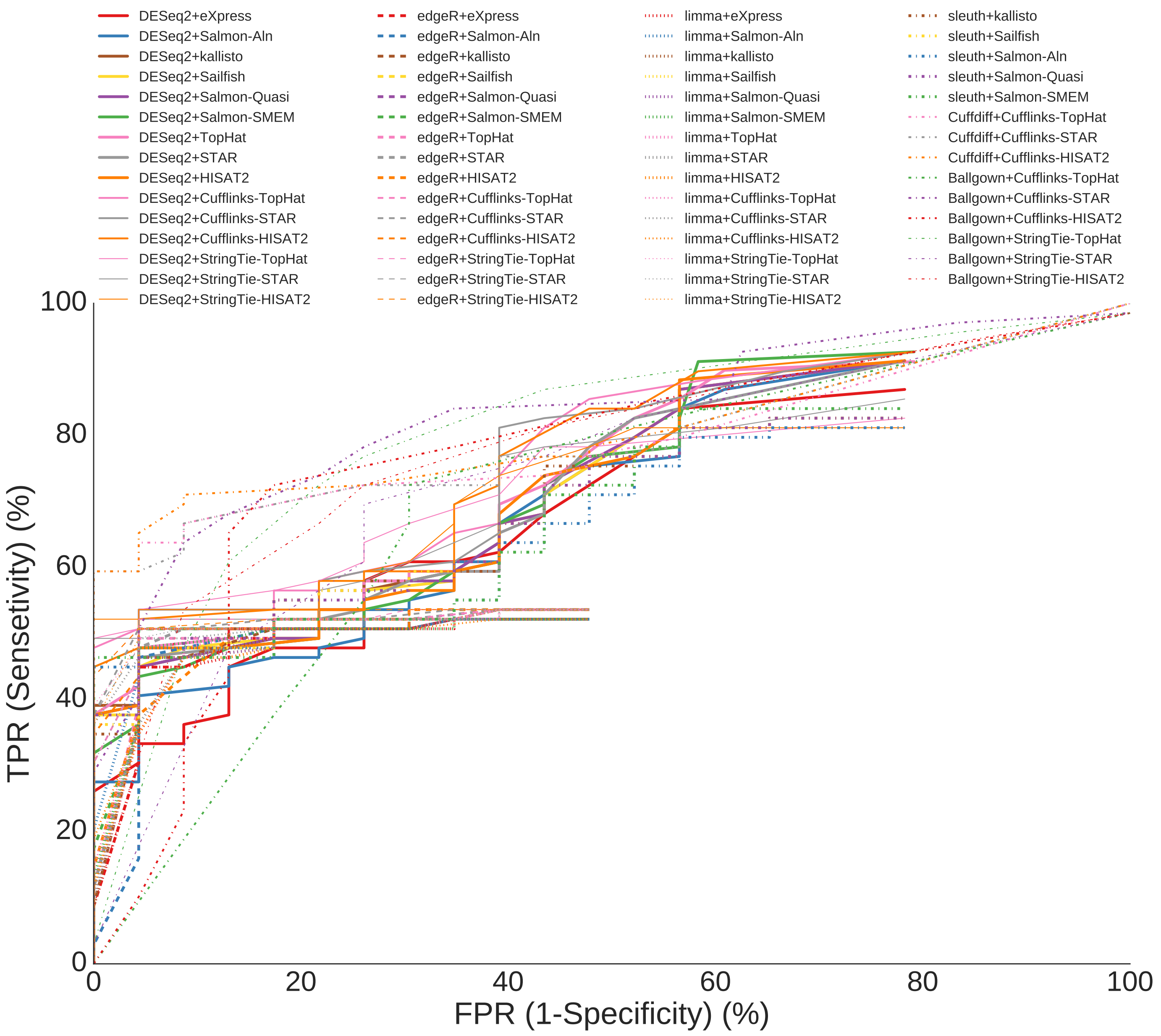

Supplementary Figure 35 | ROC analysis of ERCC genes for SEQC-A vs. SEQC-B differential analysis.

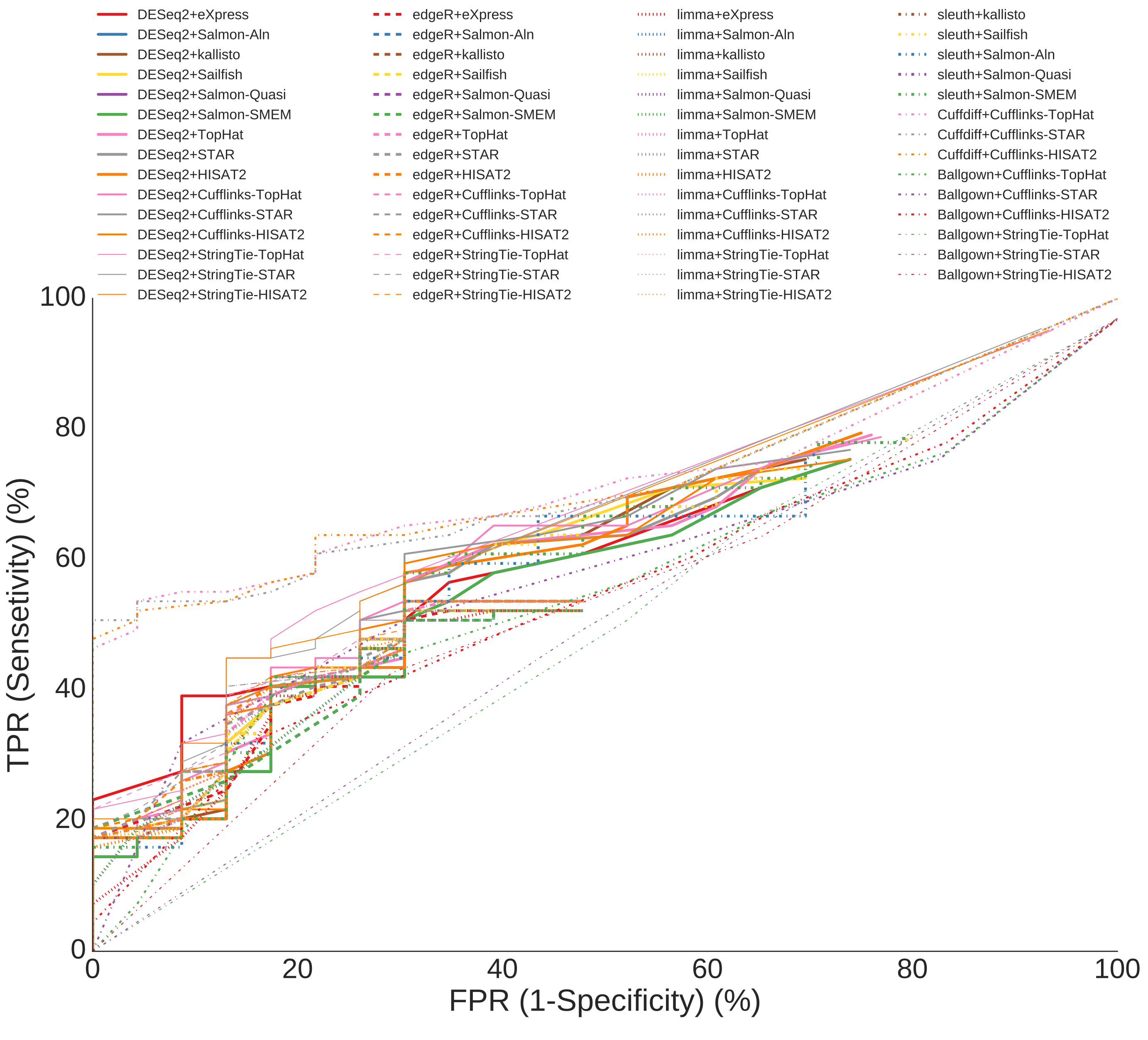

Supplementary Figure 36 | ROC analysis of ERCC measured genes for SEQC-C vs. SEQC-D differential analysis.

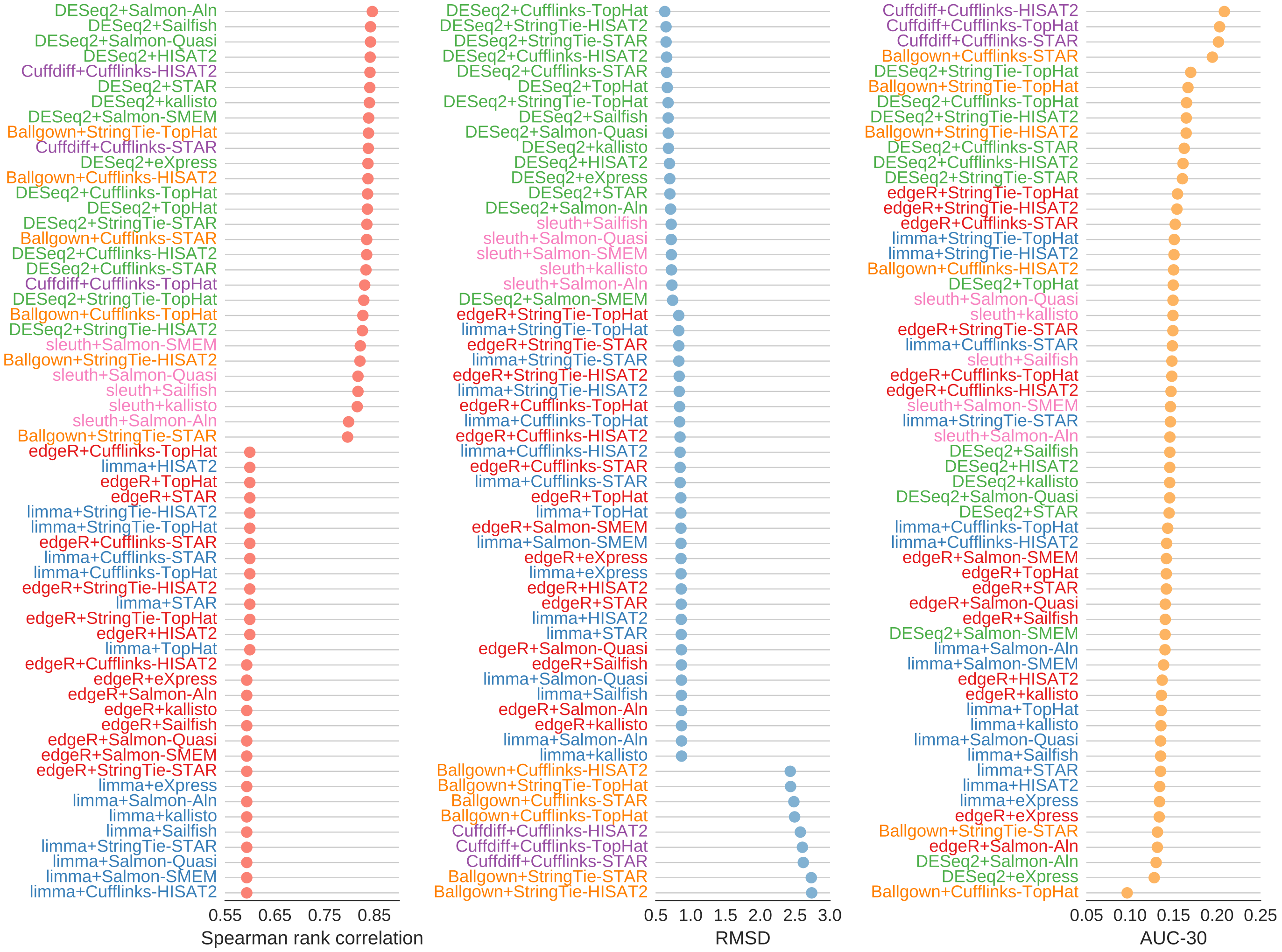

Supplementary Figure 37 | Spearman rank correlation, root-mean-score-deviation (RMSD), and AUC-30 scores for differential analysis of ERCC genes on SEQC-A vs. SEQC-B samples. Spearman rank correlation and RMSD scores are measured between the designed log2-fold change of the ERCC genes and RNA-seq predictions. AUC-30 score represents the area under the ROC curve up to the false positive rate of 30%

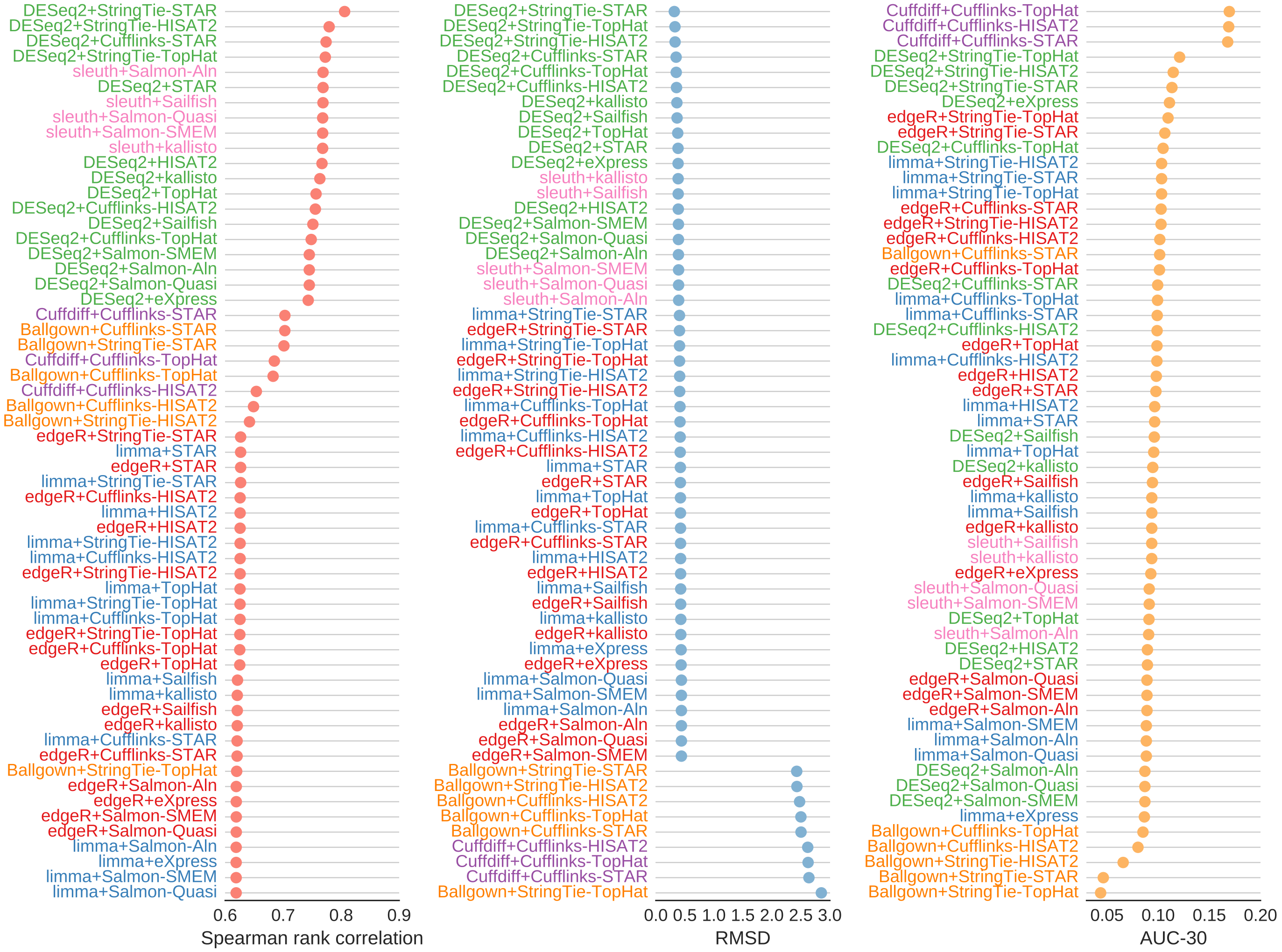

Supplementary Figure 38 | Spearman rank correlation, root-mean-score-deviation (RMSD), and AUC-30 scores for differential analysis of ERCC genes on SEQC-C vs. SEQC-D samples. Spearman rank correlation and RMSD scores are measured between the designed log2-fold change of the ERCC genes and RNA-seq predictions. AUC-30 score represents the area under the ROC curve up to the false positive rate of 30%

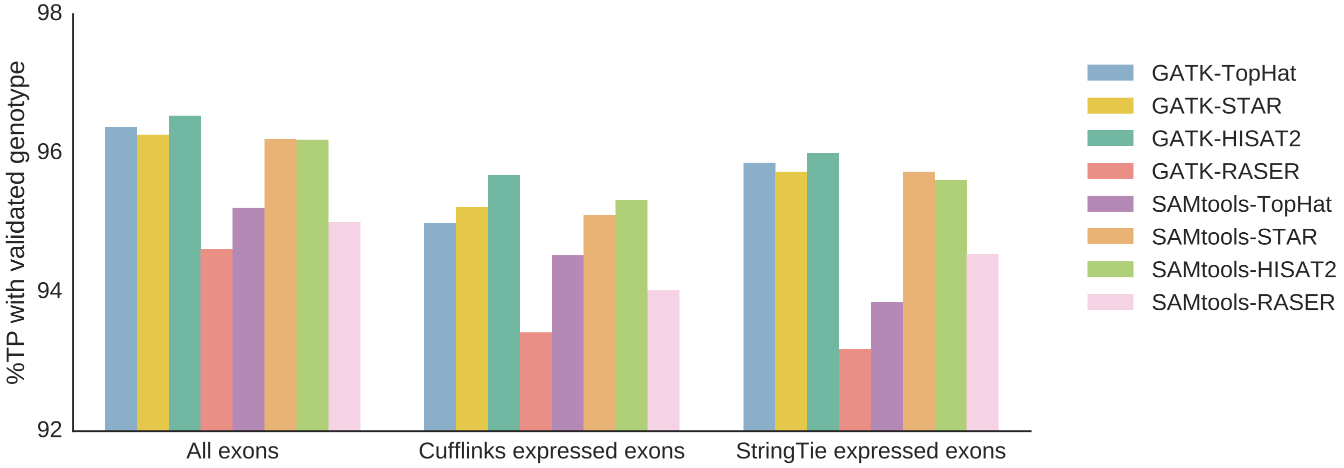

Supplementary Figure 39 | Percentage of TP variants called by different RNA-seq variant calling schemes in the NIST's NA12878 high-confidence regions that have consistent genotype prediction with the reference. Left bar-chart compares different schemes when the accuracy is measured on all variants called in the HC regions that overlap the exons in Ensembl reference annotation. Middle and right bar-charts evaluate the performance when the analysis is performed in the HC regions that overlap (expressed) exons identified respectively using Cufflinks and StringTie transcriptome reconstruction schemes.

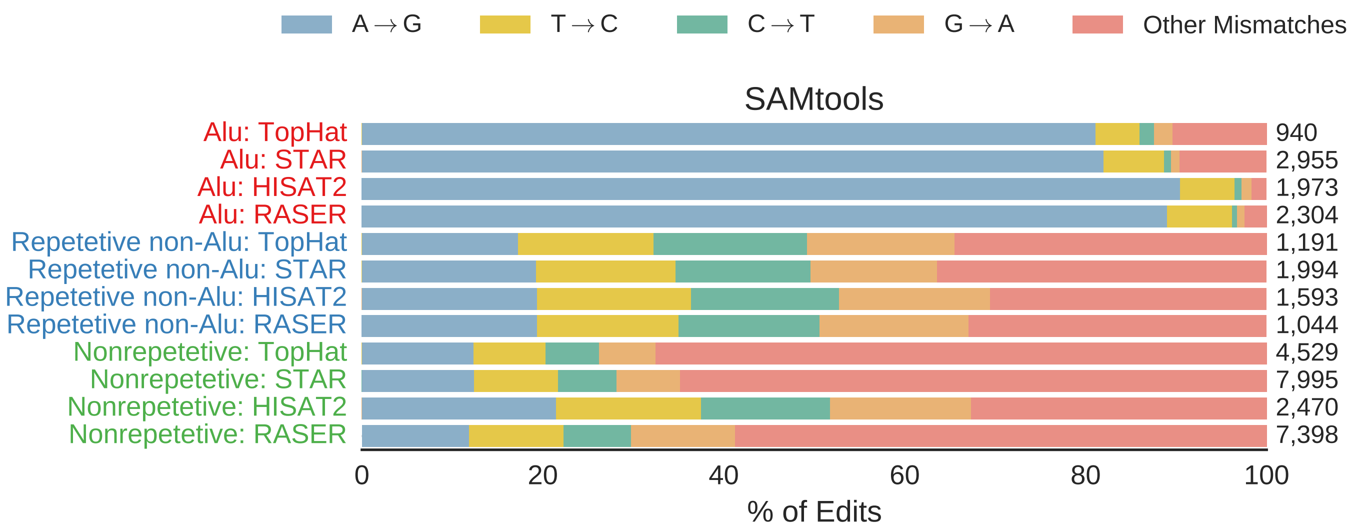

Supplementary Figure 40 | Distribution of the predicted mismatch types by SAMtools based approaches that are missed in NIST's NA12878 high-confidence calls (in expressed exons identified by StringTie.)

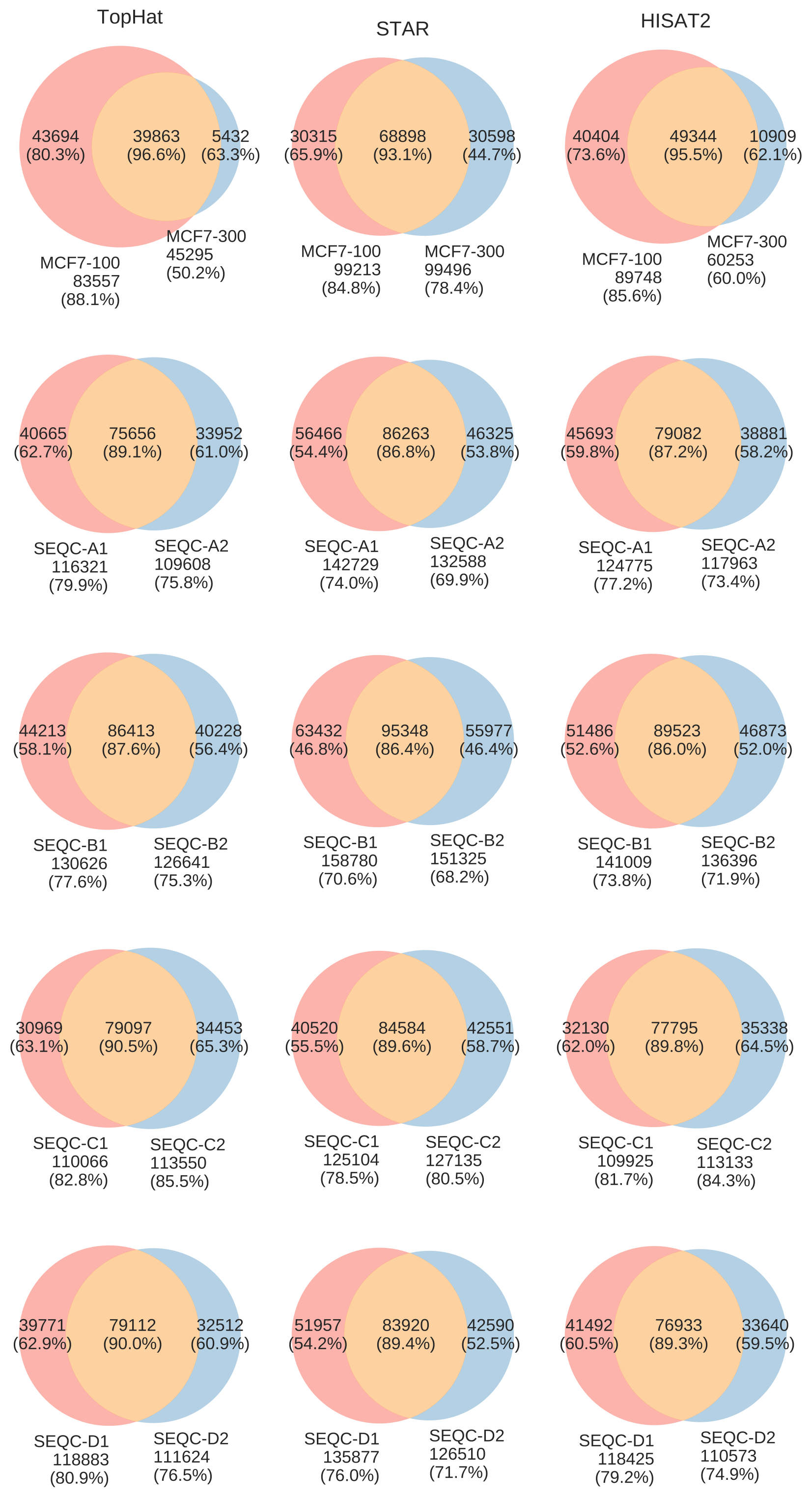

Supplementary Figure 41 | Overlap between variants predicted by GATK across MCF7 short-read samples and SEQC replicates. The sizes of the circles reflect the number of variant called each scheme. For each tool, the number of variant called and the validation rates w.r.t dbSNP database (in parentheses) are shown. Validation rates for each subset of calls are also shown on the Venn diagram.

a

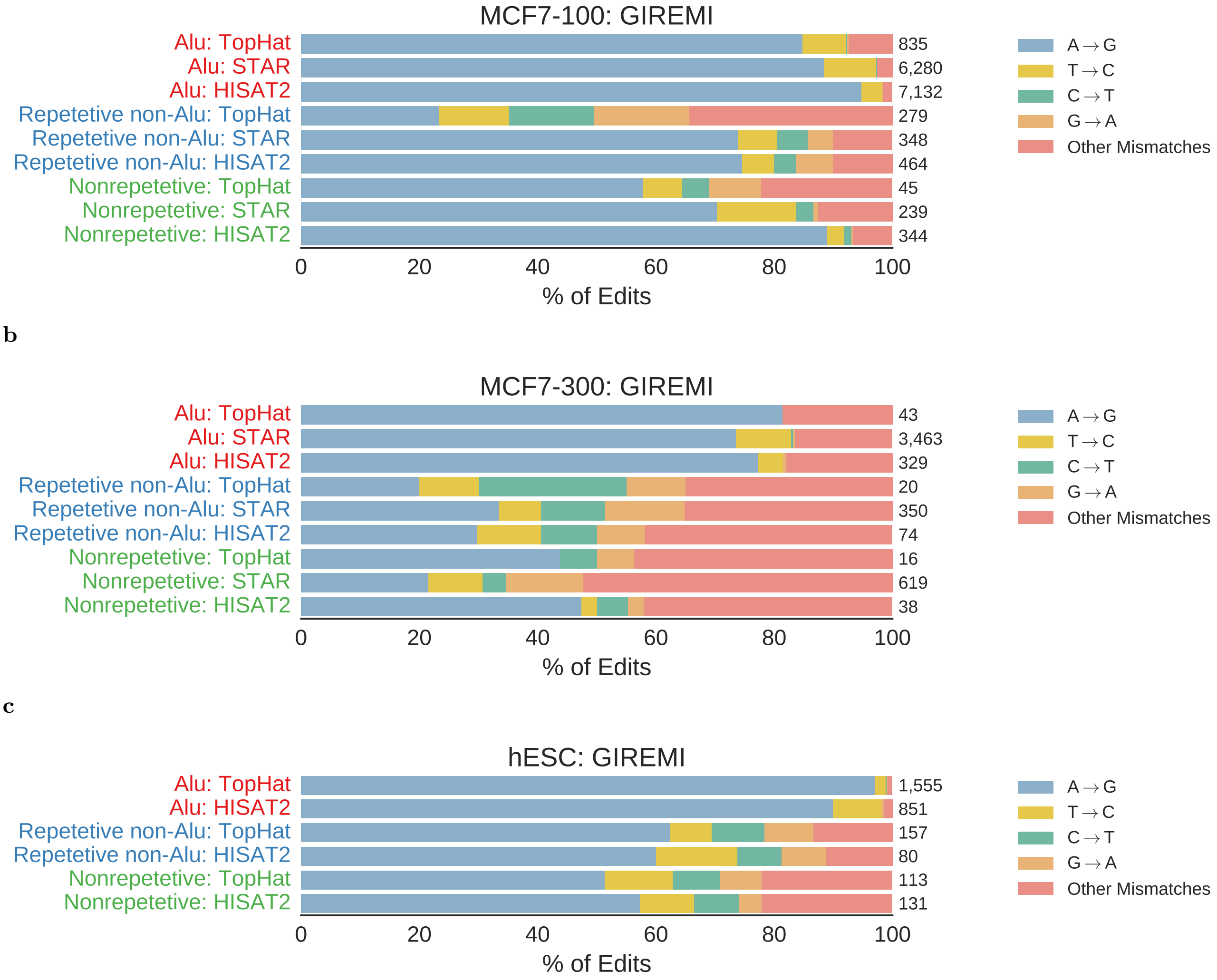

Supplementary Figure 42 | Distribution of the predicted RNA editing types by GIREMI using different alignment approaches on different genomic regions.

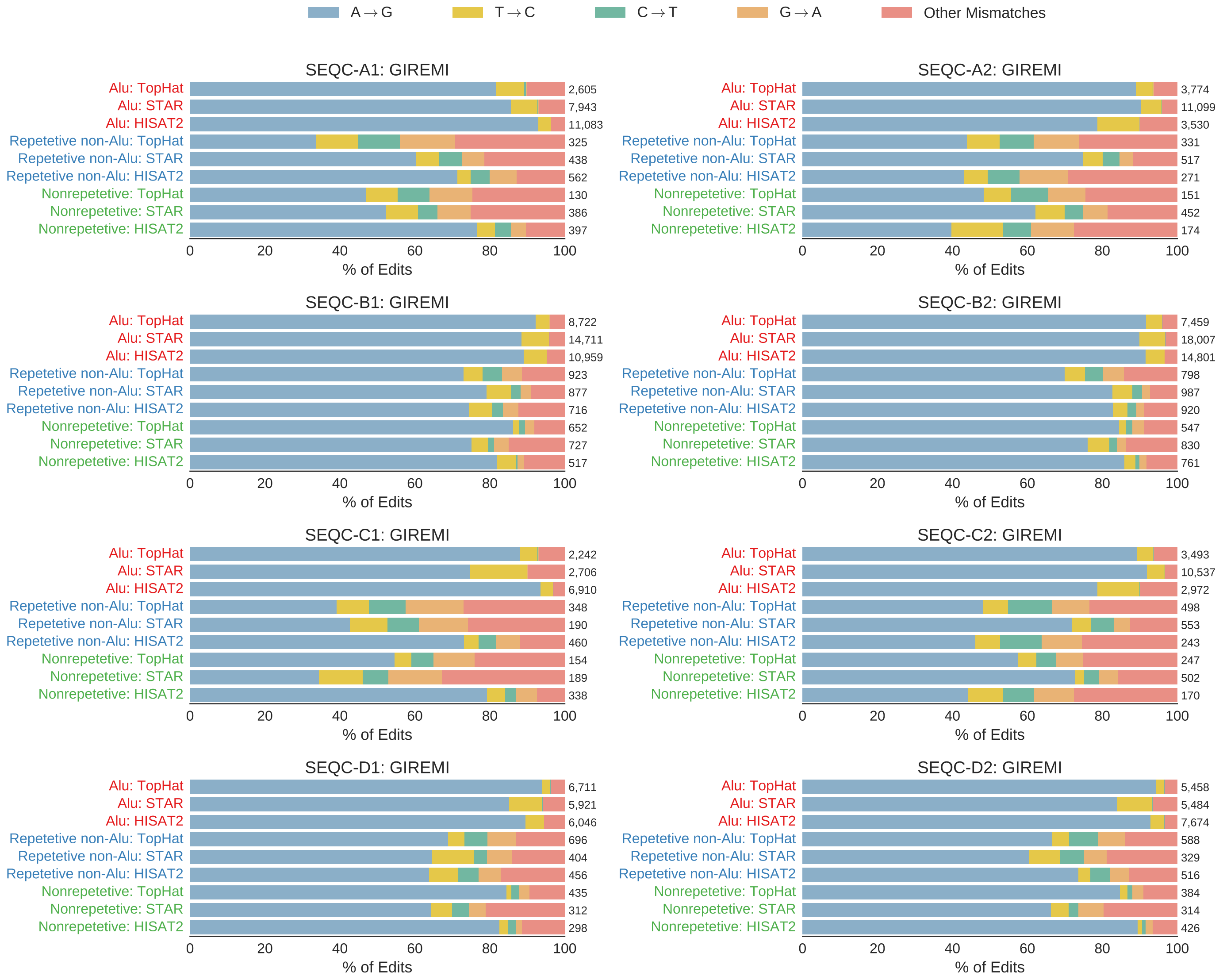

Supplementary Figure 43 | Distribution of the predicted RNA editing types for SEQC samples by GIREMI using different alignment approaches on different genomic regions.

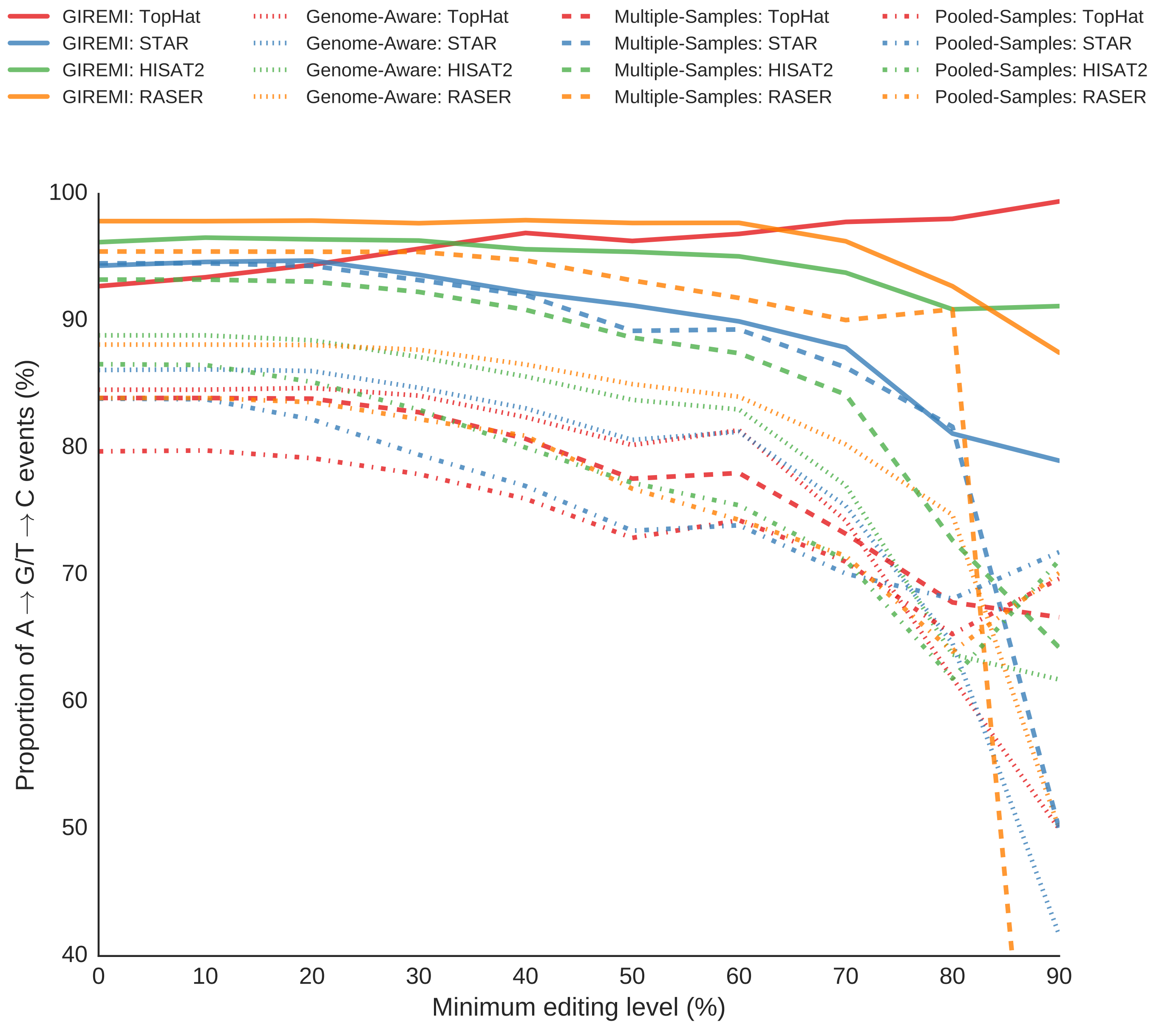

Supplementary Figure 44 | Percentage of A-to-G edits for different minimum editing levels.

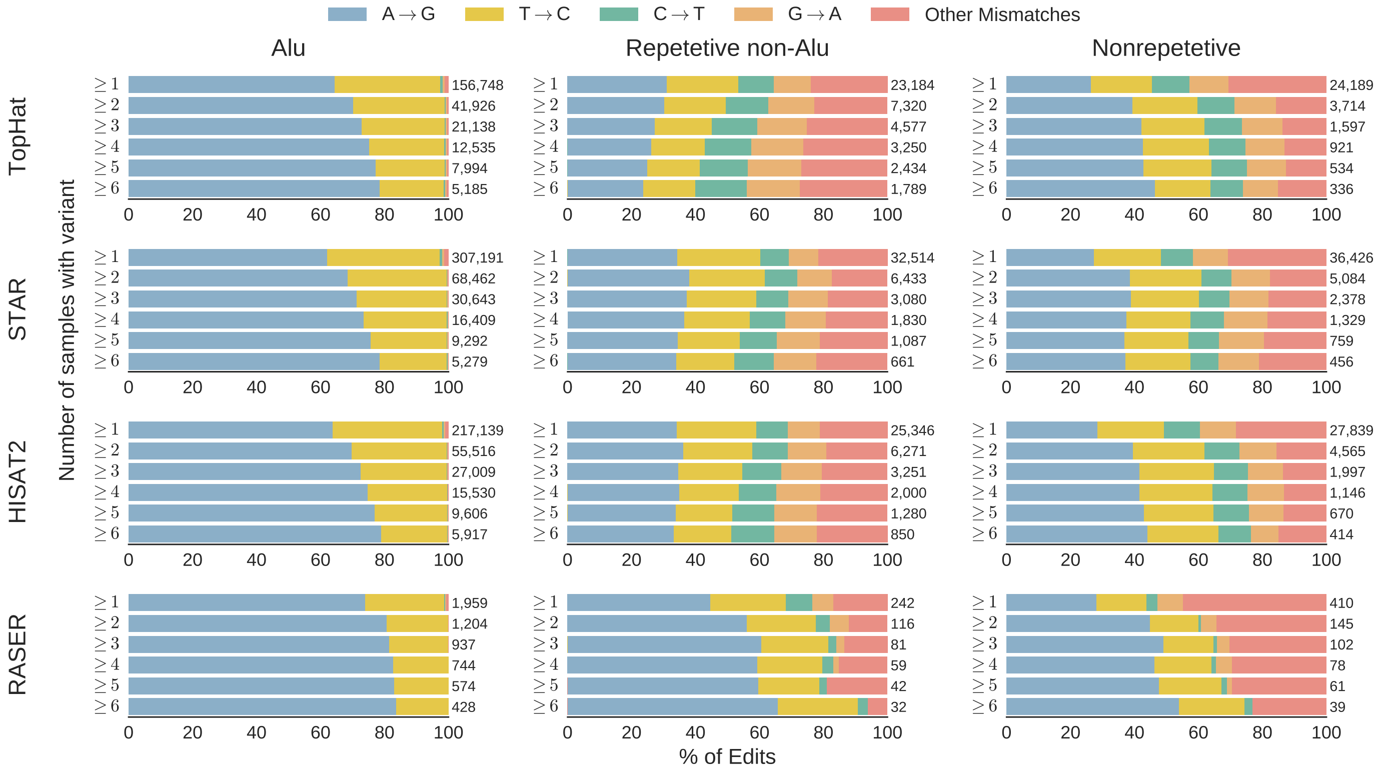

Supplementary Figure 45 | Impact of number of supporting samples on the distribution of the predicted RNA editing types (across all samples) by multiple-samples method using different alignment approaches on different genomic regions.

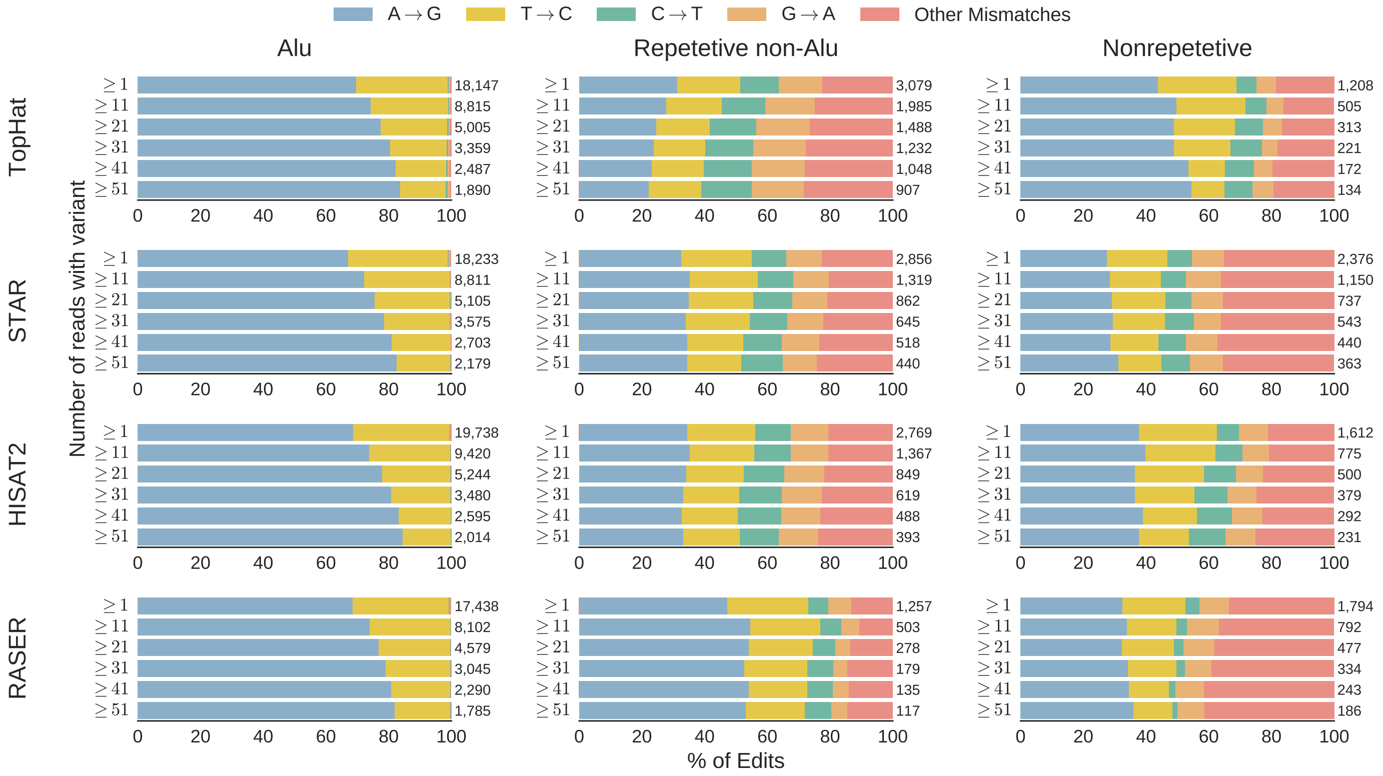

Supplementary Figure 46 | Impact of number of supporting reads on the distribution of the predicted RNA editing types for NA12878 by pooled-sample method using different alignment approaches on different genomic regions.

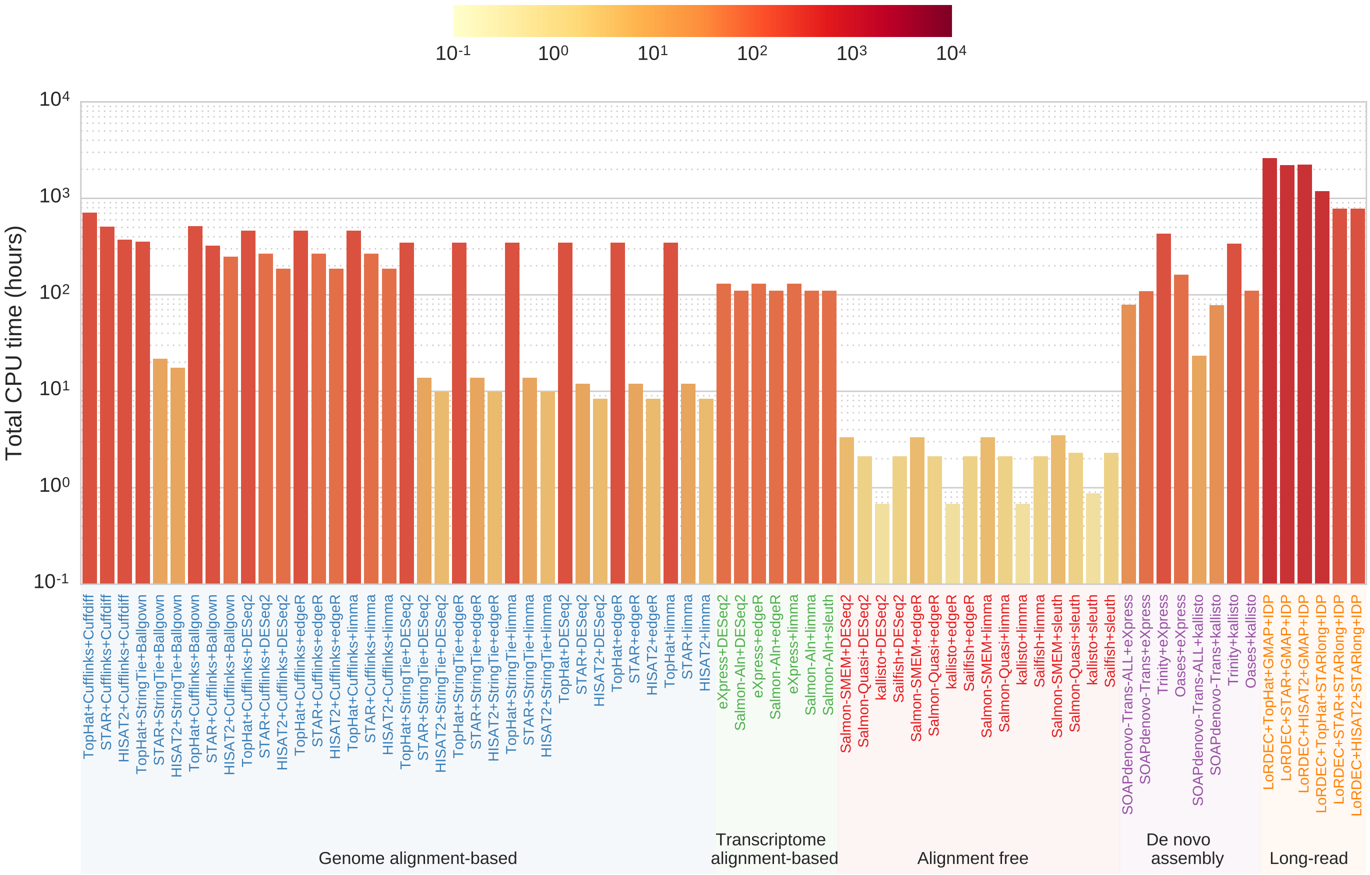

Supplementary Figure 47 | Total CPU time (in hours) for running different RNA-seq approaches.

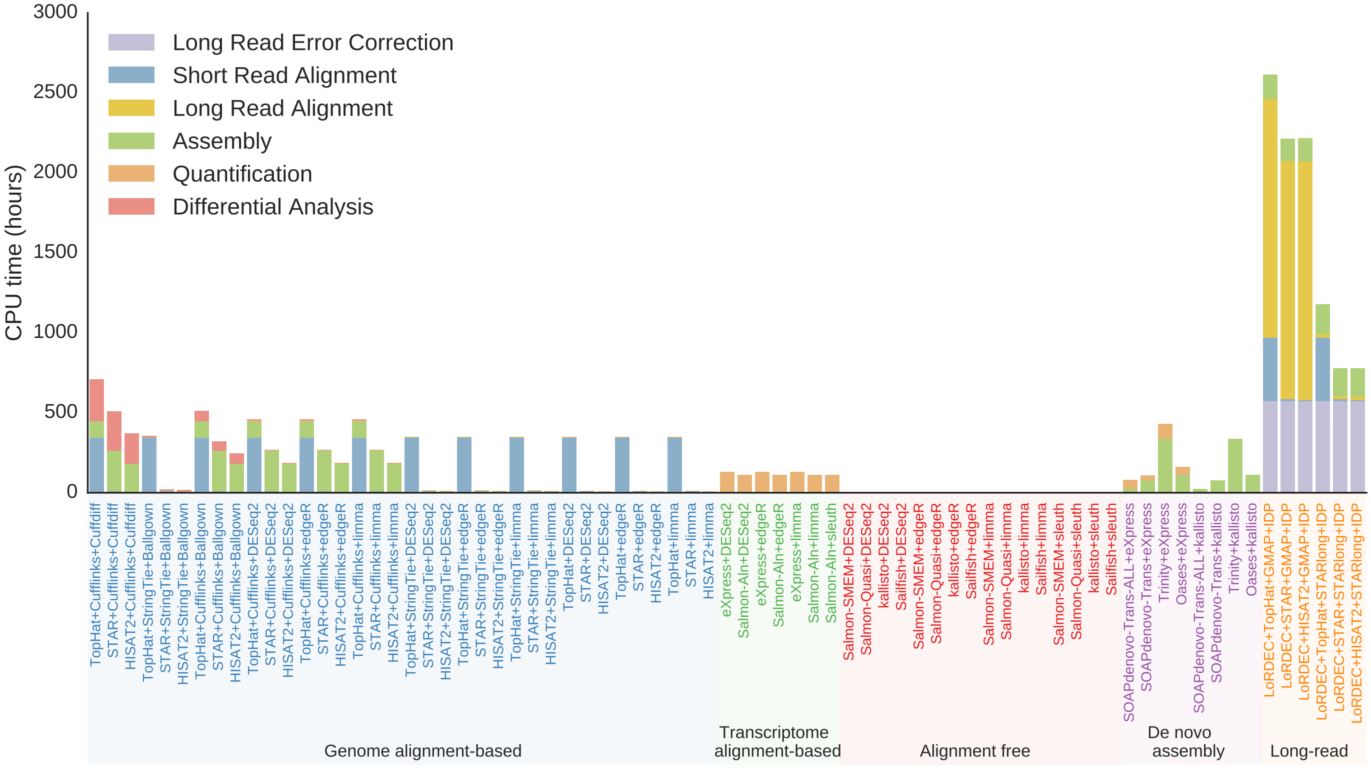

Supplementary Figure 48 | The breakdown of CPU time (in hours) of different RNA-seq analysis steps for running different RNA-seq approaches.

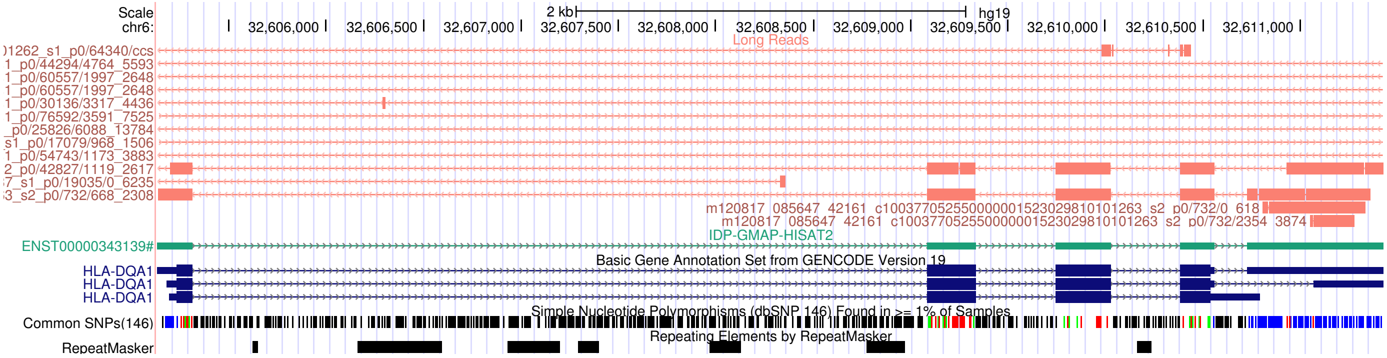

Supplementary Figure 49 | IDP prediction for HLA-DQA1 gene on hESC sample. GMAP aligned long reads, IDP predictions, GENCODE, common SNPs, and RepeatMasker annotations are shown.

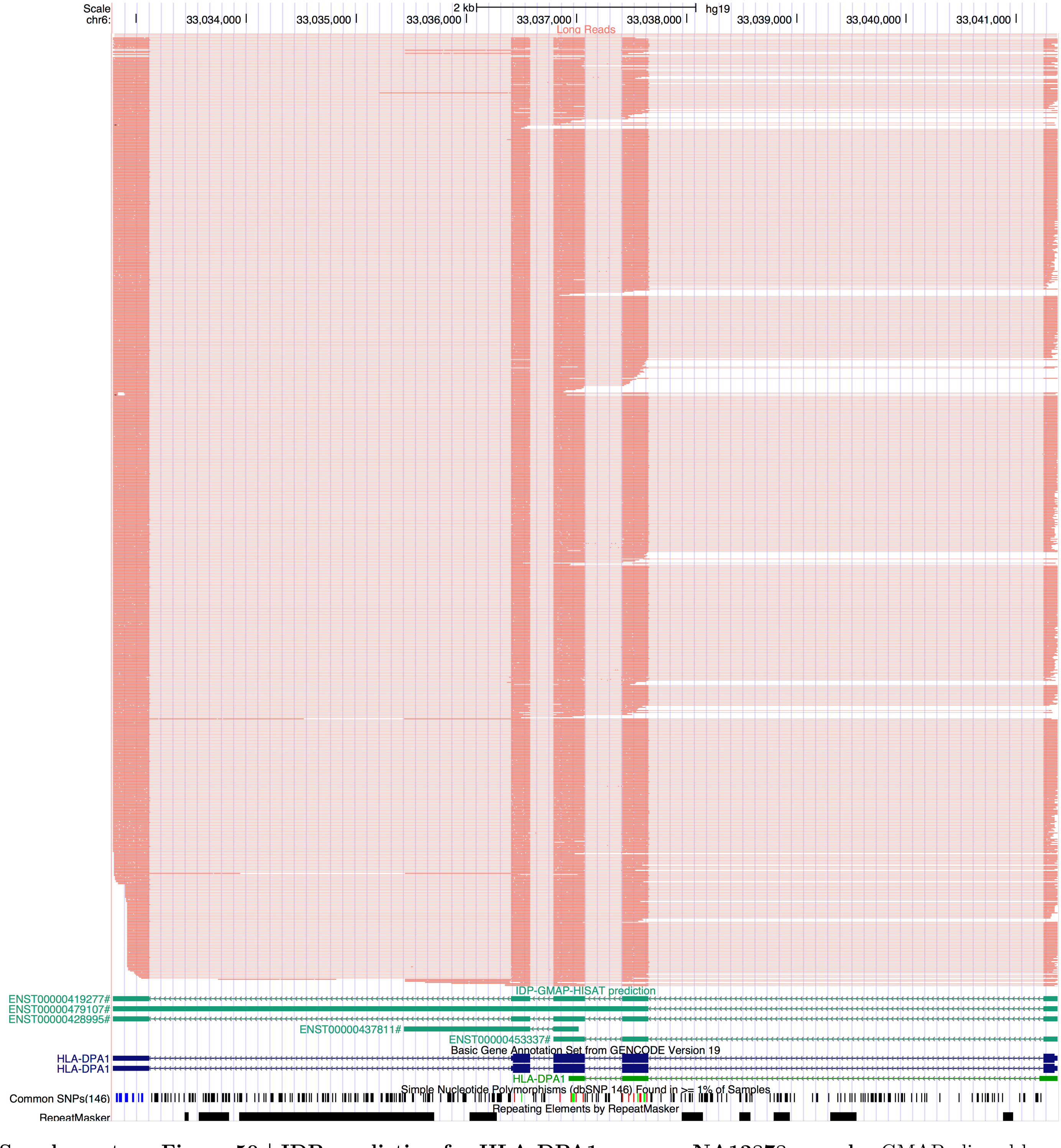

Supplementary Figure 50 | IDP prediction for HLA-DPA1 gene on NA12878 sample. GMAP aligned long reads, IDP predictions, GENCODE, common SNPs, and RepeatMasker annotations are shown.

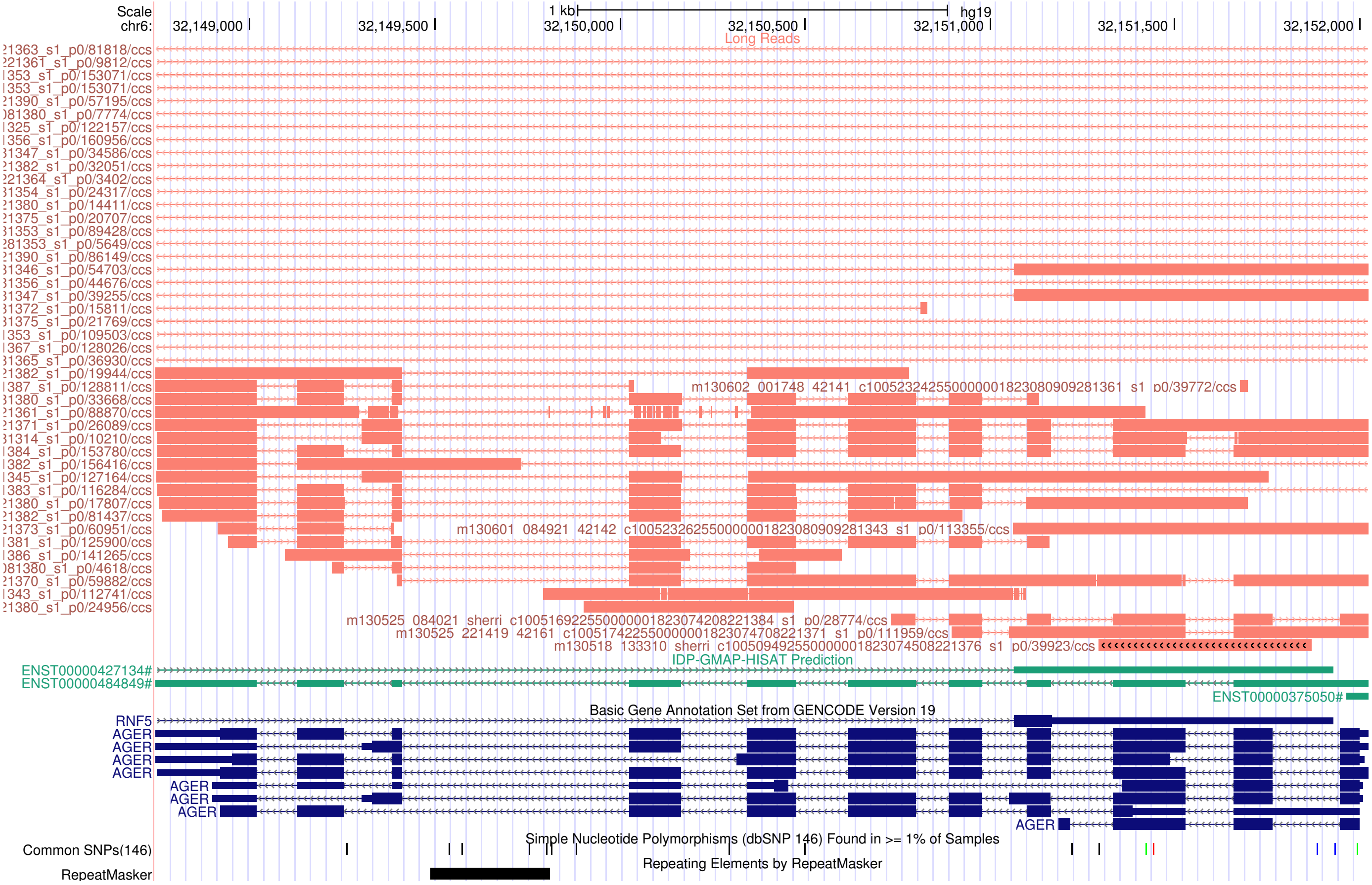

Supplementary Figure 51 | IDP prediction for AGER gene on MCF7 sample. GMAP aligned long reads, IDP predictions, GENCODE, common SNPs, and RepeatMasker annotations are shown.

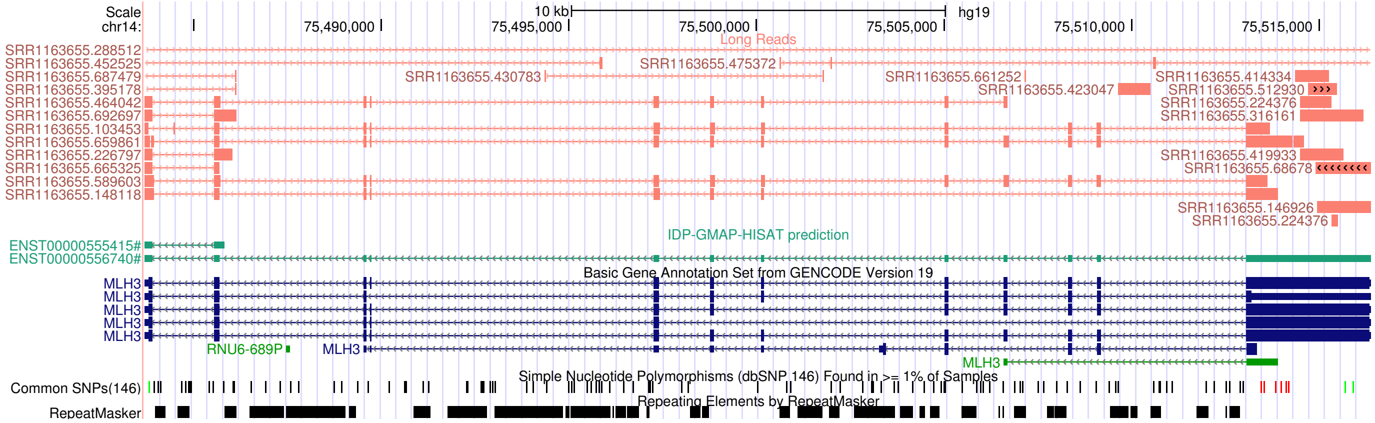

Supplementary Figure 52 | IDP prediction for MLH3 gene on NA12878 sample. GMAP aligned CCS long reads, IDP predictions, GENCODE, common SNPs, and RepeatMasker annotations are shown.

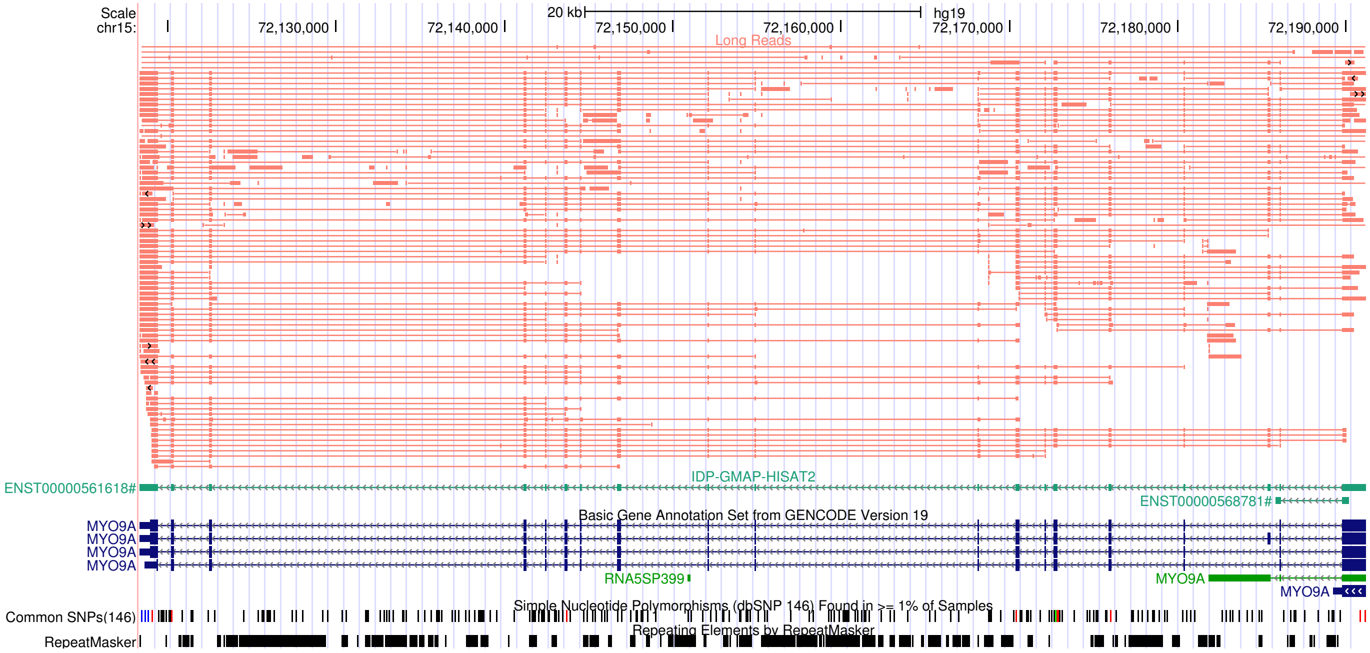

Supplementary Figure 53 | IDP prediction for MYO9A gene on MCF7 sample. GMAP aligned long reads, IDP predictions, GENCODE, common SNPs, and RepeatMasker annotations are shown.

Supplementary Tables

Supplementary Table 1 | Summary of the datasets used in this study.

Sample	Sample type	Platform	Read Type	Read length*	#reads	#bps
NA12878	Normal	Illumina	PE	101-bp	115.4M	23.3G
		PacBio	CCS	1.2 Kbp	715.9K	851.4M
MCF7	Cancer	Illumina	PE	100-bp	63.7M	12.7G
		Illumina	PE	300-bp	42.7M	25.6G
		PacBio	ROI	1.9 Kbp	7.4M	14.1G
hESC	Embryonic stem cell	Illumina	SE	101-bp	205.0M	20.7G
		PacBio	Raw	1.3 Kbp	7.8M	10.3G
SEQC-A1	Human whole body reference RNA + ERCC	Illumina	PE	101-bp	100.4M	20.3G
SEQC-A2		Illumina	PE	101-bp	91.8M	18.5G
SEQC-B1	Human brain reference RNA + ERCC	Illumina	PE	101-bp	111.0M	22.4G
SEQC-B2		Illumina	PE	101-bp	112.4M	22.7G
SEQC-C1	Mixture of samples A and B in 3:1 ratio	Illumina	PE	100-bp	91.5M	18.3G
SEQC-C2		Illumina	PE	100-bp	113.2M	22.6G
SEQC-D1	Mixture of samples A and B in 1:3 ratio	Illumina	PE	100-bp	111.0M	20.0G
SEQC-D2		Illumina	PE	100-bp	100.2M	20.6G

*For long reads, the mean read length is shown.
PE: paired-end, SE: single-end, CCS: circular-consensus sequences, ROI: Reads Of Insert.

Supplementary Table 2 | RNA-seq tools, their versions, and command line options used in the analysis.

Task	Tool	Version	Important command-line options
Alignment	TopHat	2.0.14	–no-coverage-search
	STAR	2.4.2a	–twopassMode Basic –outFilterType BySJout
	HISAT2	2.0.1-beta	–dta (or –dta-cufflinks)
	RASER	0.52	-b 0.03
Short-read Assembly	Cufflinks	2.2.1	–frag-bias-correct
	StringTie	1.2.1	-v -B
De novo Assembly	SOAPdenovo-Trans	1.04	-K 25
	Oases	0.2.09 (Velvetv1.2.10)	(velveth haslength: 25) (velvetg options: -read_trkg yes)
	Trinity	2.1.1	–normalize_reads
	Trimmomatic	0.35	

Continued on next page

Task	Tool	Version	Important command-line options
Long-read analysis tools	LoRDEC	0.6	-k 23 -s 3
	GMAP	12/31/15	-f 1
	STARlong	2.5.1b	Followed the recommended options used in [1] : –outSAMattributes NH HI NM MD –readNameSeparator space –outFilterMultimapScoreRange 1 –outFilterMismatchNmax 2000 –scoreGapNoncan -20 –scoreGapGCAG -4 –scoreGapATAC -8 –scoreDelOpen -1 –scoreDelBase -1 –scoreInsOpen -1 –scoreInsBase -1 –alignEndsType Local –seedSearchStartLmax 50 –seedPerReadNmax 100000 –seedPerWindowNmax 1000 –alignTranscriptsPerReadNmax 100000 –alignTranscriptsPerWindowNmax 10000 –outSAMstrandField intronMotif –outSAMunmapped Within
	IDP	0.1.9 *	
Quantification	eXpress	1.5.1 (bowtie2 v2.2.7)	(bowtie2 options: -a -X 600 –rdg 6,5 –rfg 6,5 –score-min L,-.6,-.4 –no-discordant –no-mixed)
	kallisto	0.42.4	
	Sailfish	0.9.0	
	Salmon-Aln	0.6.1	
	Salmon-SMEM	0.6.1	index: –type fmd<br>quant: -k,19
	Salmon-Quasi	0.6.1	index: –type quasi -k 31
	featureCounts	1.5.0-p1	-p -B -C

Continued on next page

*IDP is implemented with some modifications to support different aligners

Task	Tool	Version	Important command-line options
Differential expression analysis	DESeq2	1.14.1	
	edgeR	3.16.5	
	limma	3.30.7	
	Cuffdiff	2.2.1	–frag-bias-correct –emit-count-tables
	Ballgown	2.6.0	
	Tablemaker	2.1.1	
	sleuth	0.28.1	
Variant Calling	SAMtools	1.2 (bcftools v1.2)	samtools mpileup -C50 -d 100000bcftools filter -s LowQual -e '%QUAL<20 —— DP>10000'
	GATK	v3.5-0-g36282e4 (picard 1.129)	*Picard AddOrReplaceReadGroups:* SO=coordinate *Picard MarkDuplicates:* CREATE_INDEX=true VALIDATION_STRINGENCY=SILENTGATK *SplitNCigarReads:* -rf ReassignOneMappingQuality -RMQF 255 -RMQT 60 -U ALLOW_N_CIGAR_READSGATK *HaplotypeCaller:* -stand_call_conf 20.0 -stand_emit_conf 20.0 -A StrandBiasBySample -A StrandAlleleCountsBySampleGATK *VariantFiltration:* -window 35 -cluster 3 -filterName FS -filter "FS >30.0" -filterName QD -filter "QD <2.0"
RNA Editing	GIREMI	0.2.1	
	Varsim	0.5.1	
RNA Fusion	FusionCatcher	0.99.5a beta	
	JAFFA	1.0.6	
	SOAPfuse	1.27	
	STAR-Fusion	0.7.0	
	TopHat-Fusion	2.0.14	

.

Supplementary Table 3 | CPU time (in hours) for running different short-read alignment tools on studied samples.

**Sample**	**TopHat**	**STAR**	**HISAT2**
NA12878	228.0	5.6	4.5
MCF7-100	211.6	12.0	3.1
MCF7-300	1488	9.2	2.4
hESC	278.4	11.2	4
SEQC-A1	241.5	9.1	5.4
SEQC-A2	220.8	11.0	4.7
SEQC-B1	255.3	12.4	5.2
SEQC-C1	190.0	7.0	2.7
SEQC-C2	276.0	8.8	7.0
SEQC-D1	186.3	7.2	8.8
SEQC-D2	277.0	7.2	5.3
Average	342.2	9.2	4.8

Supplementary Table 4 | CPU time (in hours) for running different transcriptome reconstruction tools.

Assembler	Aligner	Sample												Av.
		**NA12878**	**MCF7-100**	**MCF7-300**	**hESC**	**SEQC-A1**	**SEQC-A2**	**SEQC-B1**	**SEQC-B2**	**SEQC-C1**	**SEQC-C2**	**SEQC-D1**	**SEQC-D2**	
Cufflinks	TopHat	116	260	17	60	95	105	97	110	60	120	93	105	103
	STAR	145	1310	107	432	122	105	150	146	135	100	125	140	251
	HISAT2	223	480	10	177	137	106	145	142	130	133	130	290	175
StringTie	TopHat	5.3	3	2.3	3.9	2.1	1.9	2.5	2.1	2.5	2.5	2.5	2.0	2.7
	STAR	6	4.1	2.7	4.1	2	1.9	2.1	2.3	2.0	2.5	2.9	2.1	2.9
	HISAT2	6.9	3.9	4.1	3	2.1	2	2.5	2.8	2.0	2.8	2.9	2.5	3.1
IDP-GMAP	TopHat	141	136	149	182	-	-	-	-	-	-	-	-	152
	STAR	140	136	132	150	-	-	-	-	-	-	-	-	140
	HISAT2	140	148	185	123	-	-	-	-	-	-	-	-	149
IDP-STARlong	TopHat	146	262	200	140	-	-	-	-	-	-	-	-	187
	STAR	165	211	150	167	-		-	-	-	-	-	-	173
	HISAT2	238	211	112	150	-	-	-	-	-	-	-	-	178

Supplementary Table 5 | CPU time (in hours) and peak memory required (in GB) for running different transcriptome reconstruction tools. For SOAPdenovo-Trans, Trinity, and Oases running time includes the read normalization step.

**Sample**	**SOAPdenovo-Trans-ALL**		**SOAPdenovo-Trans**		**Trinity**		**Oases**	
	**Time (h)**	**Memory (GB)**	**Time (h)**	**Memory (GB)**	**Time (h)**	**Memory (GB)**	**Time (h)**	**Memory (GB)**
NA12878	26.1	27	63	17	219	22	81	16
MCF7-100	21	50	48	29	372	30	93	29
MCF7-300	21	50	51	29	261	26	51	14
hESC	-	-	147	99	495	210	213	90
Average	22.7	42.3	77.3	43.5	336.8	72.0	109.5	37.3

Supplementary Table 6 | Performance of different long-read error correction schemes.

	**MCF-7**				**hESC**		
**Short Read Used**		**100-bp**		**300-bp**		**101-bp**	
**Method**	Original Reads	LSC	LoRDEC	LoRDEC	Original Reads	LSC	LoRDEC
**Elapsed CPU time (in hours)**	-	43200	513	667	-	58920	536
**#reads_aligned**	9.49M	9.66M	9.80M	9.78M	8.87M	5.11M	8.97M
**#reads mapq>0**	8.91M	9.52M	9.32M	9.30M	7.65M	5.01M	8.00M
**Mean edit distanc**	8.20	5.80	4.60	4.65	11.50	6.50	5.20
**% edit distance**	9.90	4.40	3.30	3.37	11.40	5.90	4.60
**% Gain**	-	54.40	65.20	64.20	-	36.10	63.70
**% Sensitivity**	-	62.80	70.30	70.90	-	42.50	70.80

Supplementary Table 7 | Number of mapped reads by different long-read alignment techniques.

Sample	GMAP			STARlong		
	**Uniquely mapped**	**Multi-mapped**	**Unmapped**	**Uniquely mapped**	**Multi-mapped**	**Unmapped**
NA12878	441,276	272,524	2,102	569,264	2,745	143,893
MCF-7 (corrected with 300-bp short reads)	2,286,589	4,593,842	497,765	4,546,615	17,462	2,814,119
MCF-7 (corrected with 100-bp short reads)	2,293,071	4,599,601	485,524	4,593,350	18,339	2,766,507
hESC	2,614,085	3,862,786	1,339,833	3,932,904	303,592	3,580,208

Supplementary Table 8 | CPU time (in hours) of different long-read alignment techniques.

Sample	GMAP	STARlong
NA12878	56.0	7.2
MCF-7 (corrected with 300-bp short reads)	2027.0	30.8
MCF-7 (corrected with 100-bp short reads)	2068.5	29.16
hESC	1816.0	19.7
Average	1491.8	21.7

Supplementary Table 9 | CPU time (in hours) for running different abundance estimation schemes.

Sample	eXpress	Salmon-Aln	kallisto	Sailfish	Salmon-Quasi	Salmon-SMEM
NA12878	83	85	0.7	2.0	2.5	2.5
MCF7-100	140	140	0.5	1	1	2.4
MCF7-300	268	33	0.9	3.5	2	4
hESC	50	33	0.7	2	2	4
SEQC-A1	74	75	0.7	1.5	2	5
SEQC-A2	97	98	0.8	3	2	2.6
SEQC-B1	67	67	0.9	2	4	4
SEQC-B2	67	67	0.8	2	2	3.3
SEQC-C1	250	251	0.5	2	2	3.3
SEQC-C2	283	285	0.5	2	3	3.3
SEQC-D1	112	114	0.5	2	1.5	2.5
SEQC-D2	69	70	0.5	2	1.5	2.5
Average	130	109.9	0.67	2.1	2.1	3.3

Supplementary Table 10 | CPU time (in hours) for running different differential expression schemes (average running times are reported for SEQC samples A-B and C-D differential analysis.)

Sample	DESeq2	edgeR	limma	Cuffdiff	Ballgown	sleuth
TopHat	5.1	5.1	5.1	-	-	-
STAR	2.7	2.7	2.7	-	-	-
HISAT2	3.5	3.5	3.5	-	-	-
TopHat+Cufflinks	15.0	15.0	15.0	264	67	-
STAR+Cufflinks	6.3	6.3	6.3	249	61	-
HISAT2+Cufflinks	6.4	6.4	6.4	192	67	-
TopHat+StringTie	3.1	3.1	3.1	-	8.6	-
STAR+StringTie	1.6	1.6	1.6	-	9.5	-
HISAT2+StringTie	1.9	1.9	1.9	-	9.5	-
eXpress	0.01	0.01	0.01	-	-	-
Salmon-Aln	0.01	0.01	0.01	-	-	-
kallisto	0.01	0.01	0.01	-	-	0.2
Sailfish	0.01	0.01	0.01	-	-	0.2
Salmon-SMEM	0.01	0.01	0.01	-	-	0.2
Salmon-Quasi	0.01	0.01	0.01	-	-	0.2

Supplementary Table 11 | CPU time (in hours) for running different variant calling schemes on NA12878 sample.

Variant Caller	Aligner			
	TopHat	STAR	HISAT2	RASER
GATK	20.5	29	29	22
SAMtools	9	26.5	29.75	22.5

Supplementary Table 12 | CPU time (in hours) for running different RNA Editing schemes.

RNA Editing Tool	Aligner			
	**TopHat**	**STAR**	**HISAT2**	**RASER**
GIREMI	0.5	0.5	1	0.2
genome-aware	0.05	0.05	0.03	0.05
multiple-samples	0.7	0.7	0.6	0.7
pooled-samples*	0.3	0.4	0.3	0.3

*For pooled-samples method, the variant-calling should be conducted on pooled alignment from all samples that can take 1000-3700 CPU hours.

Supplementary Table 13 | CPU time (in hours) for running different RNA fusion detection schemes.

Sample	FusionCatcher	JAFFA	STAR-Fusion	SOPAfuse	TopHat-Fusion
MCF7-100	88	25	2	36	144
MCF7-300	368	57	-	-	-

Supplementary Table 14 | Ensembl IDs of top 10 overexpressed genes in different samples relative to NA12878 predicted by different quantification schemes.

Sample	Transcript Quantification Approach		
	**Cufflinks-TopHat**	**StringTie-HISAT2**	**Salmon-SMEM**
MCF7-100	ENSG00000252929	ENSG00000160182	ENSG00000160182
	ENSG00000251718	ENSG00000265150	ENSG00000160180
	ENSG00000156508	ENSG00000106541	ENSG00000089356
	ENSG00000252835	ENSG00000235123	ENSG00000266422
	ENSG00000200687	ENSG00000199916	ENSG00000106541
	ENSG00000171345	ENSG00000252678	ENSG00000235123
	ENSG00000207449	ENSG00000160180	ENSG00000199916
	ENSG00000170540	ENSG00000171345	ENSG00000252678
	ENSG00000263597	ENSG00000225410	ENSG00000171345
	ENSG00000252481	ENSG00000265735	ENSG00000202364
MCF7-300	ENSG00000160182	ENSG00000160182	ENSG00000160182
	ENSG00000156508	ENSG00000160180	ENSG00000160180
	ENSG00000112306	ENSG00000106541	ENSG00000266422
	ENSG00000196747	ENSG00000235123	ENSG00000106541
	ENSG00000182611	ENSG00000252678	ENSG00000235123
	ENSG00000089356	ENSG00000075223	ENSG00000089356
	ENSG00000184270	ENSG00000171345	ENSG00000171345
	ENSG00000170540	ENSG00000003989	ENSG00000167644
	ENSG00000264549	ENSG00000196136	ENSG00000259001
	ENSG00000158373	ENSG00000170421	ENSG00000196136
hESC	ENSG00000202532	ENSG00000241186	ENSG00000241186
	ENSG00000112306	ENSG00000254934	ENSG00000254934
	ENSG00000166441	ENSG00000166426	ENSG00000230798
	ENSG00000169020	ENSG00000145423	ENSG00000266422
	ENSG00000116251	ENSG00000092068	ENSG00000166426
	ENSG00000241468	ENSG00000184697	ENSG00000145423
	ENSG00000170540	ENSG00000131914	ENSG00000202364
	ENSG00000206696	ENSG00000069482	ENSG00000184697
	ENSG00000075624	ENSG00000152661	ENSG00000131914
	ENSG00000170906	ENSG00000265992	ENSG00000130182

Supplementary Table 15 | Function of genes up-regulated in hESC sample.

Gene name	Function
TDGF1	TDGF1 encodes an epidermal growth factor-related protein that plays an essential role in embryonic development and tumor growth [2]
CRABP1	CRABP1 plays an important role in retinoic acid–mediated differentiation and proliferation processes [3] and is expressed in specific and distinct spatial and temporal patterns in many tissues of the developing embryo [4].
SFRP2	SFRP2 modulates Wnt signalling which in turn regulates ventral midbrain and dopamine neuron development [5].
GJA1	GJA1 is a component of gap junctions, which regulates cell death, proliferation, and differentiation and is largely involved in embryonic development [6].
GAL	GAL, galanin and GMAP prepropeptide, is also known to represent a distinguishing molecular feature of embryonic stem cell lines [7]
LIN28A	LIN28A encodes an RNA-binding protein that regulates genes involved in developmental timing and self-renewal in embryonic stem cells [8]

Supplementary Table 16 | Relation of the up-regulated genes in MCF7 to breast cancer.

Gene name	References suggesting relation to breast cancer
TFF1	[9]
AGR2	[10]
TFF3	[11]
SERPINA3	[12]
SLC7A2	[13]
DSCAM-AS1	[14]
SEMA3C	[15]
KRT19	[16]
KRT8	[17]

Supplementary References

[1] https://github.com/PacificBiosciences/cDNA_primer/wiki/Bioinfx-study.

[2] Dono, R. *et al.* Isolation and characterization of the CRIPTO autosomal gene and its X-linked related sequence. *American journal of human genetics* **49**, 555 (1991).

[3] FARAONIO, R., GALDIERI, M. & COLANTUONI, V. Cellular retinoic-acid-binding-protein and retinol-binding-protein mRNA expression in the cells of the rat seminiferous tubules and their regulation by retinoids. *European Journal of Biochemistry* **211**, 835–842 (1993).

[4] Chen, A. C., Yu, K., Lane, M. A. & Gudas, L. J. Homozygous deletion of the CRABPI gene in AB1 embryonic stem cells results in increased CRABPII gene expression and decreased intracellular retinoic acid concentration. *Archives of biochemistry and biophysics* **411**, 159–173 (2003).

[5] Kele, J. *et al.* SFRP1 and SFRP2 dose-dependently regulate midbrain dopamine neuron development in vivo and in embryonic stem cells. *Stem cells* **30**, 865–875 (2012).

[6] Cheng, J.-C., Chang, H.-M., Fang, L., Sun, Y.-P. & Leung, P. C. TGF-β1 up-regulates connexin43 expression: A potential mechanism for human trophoblast cell differentiation. *Journal of cellular physiology* **230**, 1558–1566 (2015).

[7] Tarasov, K. V. *et al.* Galanin and galanin receptors in embryonic stem cells: accidental or essential? *Neuropeptides* **36**, 239–245 (2002).

[8] Xu, B., Zhang, K. & Huang, Y. Lin28 modulates cell growth and associates with a subset of cell cycle regulator mRNAs in mouse embryonic stem cells. *Rna* **15**, 357–361 (2009).

[9] Siu, L.-S. *et al.* TFF1 is membrane-associated in breast carcinoma cell line MCF-7. *Peptides* **25**, 745–753 (2004).

[10] Wright, T. M. *et al.* Delineation of a FOXA1/ERα/AGR2 regulatory loop that is dysregulated in endocrine therapy–resistant breast cancer. *Molecular Cancer Research* **12**, 1829–1839 (2014).

[11] Lau, W.-H. *et al.* Trefoil Factor-3 (TFF3) stimulates de novo angiogenesis in mammary carcinoma both directly and indirectly via IL-8/CXCR2. *PloS one* **10**, e0141947 (2015).

[12] Wierer, M. *et al.* PLK1 signaling in breast cancer cells cooperates with estrogen receptor-dependent gene transcription. *Cell reports* **3**, 2021–2032 (2013).

[13] Tozlu, S. *et al.* Identification of novel genes that co-cluster with estrogen receptor alpha in breast tumor biopsy specimens, using a large-scale real-time reverse transcription-PCR approach. *Endocrine-related cancer* **13**, 1109–1120 (2006).

[14] Miano, V. *et al.* Luminal long non-coding RNAs regulated by estrogen receptor alpha in a ligand-independent manner show functional roles in breast cancer. *Oncotarget* **7**, 3201 (2016).

[15] Malik, M. F. A., Satherley, L. K., Davies, E. L., Ye, L. & Jiang, W. G. Expression of semaphorin 3C in breast cancer and its impact on adhesion and invasion of breast cancer cells. *Anticancer research* **36**, 1281–1286 (2016).

[16] Saha, S. *et al.* KRT19 directly interacts with β-catenin/RAC1 complex to regulate NUMB-dependent NOTCH signaling pathway and breast cancer properties. *Oncogene* (2016).

[17] Vantangoli, M. M., Madnick, S. J., Huse, S. M., Weston, P. & Boekelheide, K. MCF-7 human breast cancer cells form differentiated microtissues in scaffold-free hydrogels. *PloS one* **10**, e0135426 (2015).
